# Prognostic value of hormone receptor expression in patients with HR+/HER2− advanced BC treated with first-line ET+CDK4/6i: results from the multicentre observational, real-world PALMARES-2 study

**DOI:** 10.1016/j.eclinm.2026.104242

**Published:** 2026-09-22

**Authors:** Francesca Ligorio, Leonardo Provenzano, Roberta Caputo, Paolo Vigneri, Mario Giuliano, Giuseppe Curigliano, Angela Toss, Andrea Botticelli, Rebecca Pedersini, Saverio Cinieri, Matteo Lambertini, Gianpiero Rizzo, Barbara Tagliaferri, Marianna Sirico, Monica Giordano, Lorenzo Gerratana, Icro Meattini, Marta Piras, Alessandra Fabi, Alberto Zambelli, Francesco Pantano, Alessandra Gennari, Nicla La Verde, Donata Sartori, Ornella Garrone, Michelino De Laurentiis, Claudia Calderaio, Giuseppe Buono, Maria Luisa d'Aulisio Garigliota, Luisa Cirillo, Valentina Guarneri, Michele Bottosso, Alice Menichetti, Gaia Griguolo, Giovanna Armani, Anthea Iacobucci, Giulia Coppola, Federica Martorana, Chiara Conti, Maria Vita Sanò, Grazia Arpino, Vincenza Cantile, Pier Paolo Berton Giachetti, Antonio Marra, Elisabetta Munzone, Ornella Ponzoni, Silvia Zaniboni, Monica Verrico, Deborah Cosentini, Riccardo Lobefaro, Edoardo Chiappe, Chiara Barletta, Irene Lanzetta, Daniela Montanari, Domiziana Alaimo, Martina Tessitori, Luca Visani, Benedetta Tinterri, Giulia Viale, Ida Paris, Alessandra Guarino, Giancarlo Pruneri, Maria Vittoria Dieci, Claudio Vernieri, PALMARES-2 group, Flavia Jacobs, Giuseppe Fotia, Giacomo Mazzoli, Beatrice Fratini, Caterina Sposetti, Matteo De Monte, Alessandra Russo, Filippo de Braud, Valentina Bianchessi, Alice Abate, Giovanna Spinosa, Enrico Magri, Annarita Verrazzo, Sara Parola, Claudia Martinelli, Daniela Cianniello, Michela Piezzo, Giusy Landa, Pietro Napolitano, Laura Trovò, Carlo Alberto Giorgi, Grazia Vernaci, Sabrina Nucera, Carlo Carnaghi, Giuliana Pavone, Paolo Colina, Alessandra Longobardi, Vittoria Molinaro, Jalissa Katrini, Laura Boldrini, Ambra Carnevale Schianca, Julian Etessami, Federica Domati, Emma Zattarin, Flavia Siciliano, Maria Vittoria Bonomo, Laura Orlando, Annamaria Quaranta, Sara Demurtas, Federica Puce, Francesca Rifaldi, Simone Figini, Lorenzo Livi, Gaia Troiani, Giovanna Masci, Angela Grieco, Luisa Carbognin, Giampaolo Bianchini, Francesca Patanè, Roberta Scafetta, Raffaella Troiano, Carmen Branni, Giulia Isingrini, Maria Silvia Cona, Carmen Giusy Rea, Daniele Generali, Andrea Vingiani, Antonino Belfiore, Paolo Baili, Ilaria Cavallo, Gianfranco Scaperrotta, Catherine Depretto, Giovanni Irmici, Elisa D'Ascoli, Alice Bonanomi, Cristina Ferraris, Gabriele Martelli, Carmen De Santis

**Affiliations:** a IFOM ETS, AIRC Institute of Molecular Oncology, Milan, Italy; b Department of Oncology and Hematology, Fondazione IRCCS Istituto Nazionale Tumori, Milan, Italy; c Department of Breast and Thoracic Oncology, Istituto Nazionale Tumori IRCCS “Fondazione G. Pascale”, Naples, Italy; d Department Clinical and Experimental Medicine, University of Catania, Catania, Italy; e U.O. Oncologia Universitaria, Humanitas Istituto Clinico Catanese, Catania, Italy; f Department of Clinical Medicine and Surgery, Università Degli Studi di Napoli Federico II, Naples, Italy; g Division of Early Drug Development for Innovative Therapy, IRCCS European Institute of Oncology, Milan, Italy; h Department of Oncology and Hematology-Oncology, University of Milan, Milan, Italy; i Department of Oncology and Hematology, Azienda Ospedaliero-Universitaria di Modena, Modena, Italy; j Department of Medical and Surgical Sciences, University of Modena and Reggio Emilia, Modena, Italy; k Department of Experimental Medicine, Sapienza University of Rome, Rome, Italy; l Department of Radiological, Oncological and Pathological Science, Sapienza University of Rome, Rome, Italy; m Medical Oncology Department, ASST Spedali Civili of Brescia, Brescia, Italy; n U.O.C. Oncologia Medica, Presidio Ospedaliero “Antonio Perrino”, Brindisi, Italy; o Department of Medical Oncology, U.O. Clinica di Oncologia Medica, IRCCS Azienda Ospedaliera Metropolitana, Genova, Italy; p Department of Internal Medicine and Medical Specialties (DiMI), School of Medicine, University of Genova, Genova, Italy; q Department of Oncology, Comprehensive Cancer Center, IRCCS Policlinico San Matteo Foundation, Pavia, Italy; r Medical Oncology Unit, ICS Maugeri IRCCS, Pavia, Italy; s Medical Oncology, Breast & GYN Unit Department of Clinical and Experimental Oncology and Hematology IRCCS Istituto Romagnolo per lo Studio Dei Tumori (IRST) “Dino Amadori”, Meldola, Italy; t SC Oncologia, ASST-Lariana, Como, Italy; u Department of Medicine (DMED), University of Udine, Udine, Italy; v Breast Medical Oncology, CRO Aviano National Cancer Institute, Aviano, Italy; w Radiation Oncology Unit, Breast Unit, Azienda Ospedaliero Universitaria Careggi, Florence, Italy; x Department of Experimental and Clinical Biomedical Sciences “M. Serio”, University of Florence, Florence, Italy; y Division of Medical Senology, IRCCS European Institute of Oncology, Milan, Italy; z UOSD Medicina di Precisione in Senologia, Fondazione Policlinico Universitario Agostino Gemelli IRCCS, Rome, Italy; aa Dipartimento di Medicina e Chirurgia, Università Degli Studi di Milano-Bicocca, Milan, Italy; ab Oncology Department, Papa Giovanni XXIII Hospital, Bergamo, Italy; ac Medical Oncology, Fondazione Policlinico Universitario Campus Bio-Medico, Rome, Italy; ad Department of Medicine and Surgery, Università Campus Bio-Medico di Roma, Rome, Italy; ae Division of Medical Oncology, Maggiore University Hospital, Novara, Italy; af Department of Translational Medicine, University of Piemonte Orientale, Novara, Italy; ag Department of Oncology, Polo Ospedaliero Luigi Sacco, ASST Fatebenefratelli Sacco, Milan, Italy; ah Oncology Unit, AULSS3 Veneziana, Mirano, Italy; ai Medical Oncology Fondazione IRCCS Ca’ Granda Ospedale Maggiore Policlinico Milano, Milan, Italy; aj Oncology 2, Veneto Institute of Oncology IOV-IRCCS, Padua, Italy; ak Department of Surgery, Oncology and Gastroenterology, University of Padua, Padua, Italy; al Division of Medical Senology, IRCCS European Institute of Oncology, Milan, Italy; am Department of Internal Medicine and Medical Therapy, University of Pavia, Pavia, Italy; an IRCCS Humanitas Research Hospital, Rozzano, Italy; ao Department of Medical Oncology, IRCCS San Raffaele Hospital, Milan, Italy; ap Department of Oncology, Vita-Salute San Raffaele University, Milan, Italy; aq Division of Gynecologic Oncology, Department of Woman and Child Health and Public Health, Fondazione Policlinico Universitario Agostino Gemelli IRCCS, Rome, Italy; ar Department of Advanced Diagnostics, Fondazione IRCCS Istituto Nazionale Dei Tumori, Milan, Italy

**Keywords:** HR+/HER2− breast cancer, CDK4/6i, Oestrogen receptor, Progesterone receptor, Real-world study

## Abstract

**Background:**

Endocrine therapy (ET) plus Cyclin Dependent Kinase 4/6 inhibitors (CDK4/6i) is the recommended first-line treatment for patients with Hormone Receptor-positive, Human Epidermal growth factor Receptor 2-negative advanced Breast Cancer (HR+/HER2− aBC). The prognostic value of the spectrum of Oestrogen Receptor (ERα) and Progesterone receptor (PgR) expression remains poorly investigated in this setting.

**Methods:**

We used updated data from the largest annotated dataset from the multicentre, real-world, Italian study PALMARES-2 (NCT06805812) to evaluate the association between ERα expression and real-world Progression-Free Survival (rwPFS), or time-to-chemotherapy (rwTTC), in patients with HR+/HER2− aBC treated with first-line ET plus CDK4/6/i. ERα expression was classified as low-intermediate (LI: 1–49%), high (H: 50–89%) or very-high (VH: 90–100%). We also assessed the prognostic value of combining PgR (<20% vs. ≥20%) and ERα expression. Multivariable Cox regression models were used to adjust associations for clinically relevant covariates.

**Findings:**

Among 4115 patients with available ERα assessment, 157 (3.8%) had ER-LI, 967 (23.5%) had ER-H and 2991 (72.7%) had ER-VH tumours. Median rwPFS and rwTTC increased progressively with increasing ERα expression (ER-LI: 10.6 and 14.3 months; ER-H: 20.5 and 29.9 months; ER-VH: 28.5 and 40.7 months, respectively; p < 0.001). PgR expression provided additional prognostic stratification, with patients with ER-VH/PgR-high tumours showing the longest median rwPFS (32.4 months, 95% CI 30.2–34.9), while those with ER-LI/PgR-low tumours had the shortest (8.3 months; 95% CI 6.9–12).

**Interpretation:**

Effectiveness of first-line ET plus CDK4/6i in patients with HR+/HER2− aBC worsens progressively with decreasing ERα expression, and PgR expression adds further prognostic discrimination, with low ERα and PgR expression identifying a subgroup of patients with particularly poor outcomes. Our findings, if confirmed by future studies, can help clinicians in personalising treatment choices and follow-up strategies in this clinical context.

**Funding:**

PALMARES-2 is an academic investigator-initiated study partially supported by 10.13039/100004325AstraZeneca/Daiichi Sankyo.

**Research in context**

**Evidence before this study**

According to the current ASCO/CAP guidelines, Hormone Receptor-positive, Human Epidermal growth factor Receptor 2-negative advanced Breast Cancer (HR+/HER2− aBC) identifies a large and heterogeneous group of patients with tumour cell nuclei staining positive for Oestrogen Receptor alpha (ERα) and/or Progesterone Receptor (PgR) in a proportion between 1% and 100%. The Cyclin-Dependent Kinase 4/6 inhibitors (CDK4/6i) palbociclib, ribociclib and abemaciclib combined with endocrine therapy (ET) have become the standard first-line treatment for almost all patients with HR+/HER2− aBC regardless of the ranges of HR expression.

HR positivity is an acknowledged prognostic and predictive factor of benefit from endocrine-based therapies in patients with HR+/HER2− aBC. However, the impact of specific ranges of ERα/PgR expression on long-term clinical benefit from ET + CDK4/6i is unknown, as pivotal randomised controlled trials did not stratify outcomes by ERα or PgR expression levels.

We searched the PubMed database from inception to April 1st, 2026, for research studies that included any combination of the terms: “CDK4/6 inhibitors”, “palbociclib”, “ribociclib”, “abemaciclib”, “hormone-receptor positive, HER2-negative breast cancer (HR+/HER2− BC)”, “Estrogen receptor”, “Progesterone receptor”, “Hormone receptor”, “efficacy”, “real-world” with no further restrictions (including no language restriction). Our search did not identify any published, large-scale, real-world study comparing the effectiveness of any or individual CDK6/4i according to ERα expression.

**Added value of this study**

PALMARES-2 (NCT06805812) is the largest real-world study evaluating the effectiveness of first-line ET + CDK4/6i in patients with HR+/HER2− aBC across 26 Italian Institutions. In this prespecified sub-analysis, we investigated the impact of ERα and PgR expression, as measured in the most recently available tumour specimen, on real-world Progression-Free Survival (rwPFS) and real-world time-to-chemotherapy (rwTTC) in 4115 patients.

We identified distinct HR expression levels associated with substantially different clinical outcomes during first-line ET + CDK4/6i. Patients with ER-high/very-high (ERα: 50–100%) AND PgR-high (PgR: 20–100%) tumours experienced excellent rwPFS and rwTTC, supporting ET + CDK4/6i as the preferred first-line treatment in this context; these patients may also be candidates for less intensive clinical and radiological monitoring. Patients with ER-high/very-high (ERα: 50–100%) PgR-low (PgR: 0–20%) or ER-low–intermediate (ERα: 1–49%) PgR-high (PgR: 20–100%) tumours still derived benefit from ET + CDK4/6i, but they may require closer follow-up, particularly in the presence of other adverse prognostic factors. By contrast, patients with ER-low–intermediate (ERα: 1–49%) PgR-low (PgR: 0–20%) tumours showed dismal prognosis, suggesting that alternative first-line strategies, such as chemotherapy, antibody–drug conjugates (e.g., trastuzumab-deruxtecan), or investigational approaches may be preferred treatment options.

Importantly, the prognostic value of ERα and PgR emerged only when these markers were assessed in tumour samples obtained immediately before first-line treatment initiation, but not in archival surgical tumour samples. This finding underscores the biological evolution of HR+/HER2− aBC under prior endocrine therapy and supports routine tumour re-biopsy at metastatic diagnosis to ensure accurate and up-to-date hormone-receptor profiling.

**Implications of all the available evidence**

Together with existing evidence, our results show that quantitative ERα and PgR expression is an important prognostic determinant in patients with HR+/HER2− aBC treated with first-line ET + CDK4/6i. Incorporating these biomarkers into clinical practice could refine biological stratification, guide treatment selection, and tailor the intensity of disease monitoring.

The finding that only recent metastatic biopsies, but not archival specimens, are prognostically informative reinforces the clinical value of tumour re-biopsy at metastatic diagnosis to reassess tumour biology, exclude phenotype switching, and obtain reliable and updated estimates of ERα and PgR expression.

## Introduction

More than half a century ago, Oestrogen Receptor alpha (ERa) expression emerged as a crucial prognostic and predictive biomarker in breast cancer,^1^ and it still represents the most important predictor of clinical benefit from endocrine-based treatments.^2^^,^^3^ According to the recommendations of the ASCO CAP 2020 guidelines,^4^ invasive breast cancers are classified as ERα-positive if at least 1% of cancer nuclei stain positive for ERα at immunohistochemistry (IHC) analysis. Recent studies and meta-analyses showed that breast malignancies expressing low ERα levels (1–10%) are biologically, immunologically and clinically similar to triple-negative breast carcinomas (TNBC).^5^, ^6^, ^7^, ^8^, ^9^, ^10^, ^11^, ^12^ Consistently, patients with ERα-low aBC are treated similarly to patients with advanced TNBC. By contrast, the prognostic and predictive significance of varying ERα expression levels across the full spectrum of ERα-positive breast malignancies (ERα: 1–100%) remains poorly characterised.

The combination of Cyclin-Dependent Kinase 4/6 inhibitors (CDK4/6i) palbociclib, ribociclib or abemaciclib with endocrine therapy (ET) represents the standard-of-care, first-line treatment option for patients with HR+/HER2− aBC regardless of ERα expression levels in tumour cells.^13^, ^14^, ^15^, ^16^, ^17^, ^18^, ^19^, ^20^, ^21^, ^22^, ^23^, ^24^, ^25^, ^26^, ^27^, ^28^ Here, we exploited the largest, real-world, annotated data collection from patients with HR+/HER2− aBC treated with first-line CDK4/6i in combination with ET and enrolled in the multicentre, Italian study PALMARES-2 (NCT06805812) to investigate the prognostic value of quantitative ERα expression in this context.^29^ We also investigated the combined prognostic value of ERα and (Progesterone Receptor) PgR expression, and we explored changes in the expression of both hormone receptors (HR) during the course of the disease and their association with first-line treatment effectiveness. Finally, we compared clinical outcomes of patients treated with palbociclib, ribociclib or abemaciclib in the whole study cohort, as well as in different ERα expression groups.

## Methods

### Study design and participants

PALMARES-2 (NCT06805812) is a retrospective-prospective, observational, real-world study enrolling consecutive patients with HR+/HER2− aBC who initiated first line treatment with ET plus CDK4/6i after January 1st, 2016 (see Supplementary Methods for details). The full list of features collected in the PALMARES-2 study is detailed in Table S1. This pre-planned analysis (Fig. 1), which was conducted after the second study data cut-off (January 2025), aimed at assessing the association between quantitative ERα expression (see “Procedures” for details), and clinical outcomes.

**Fig. 1:**
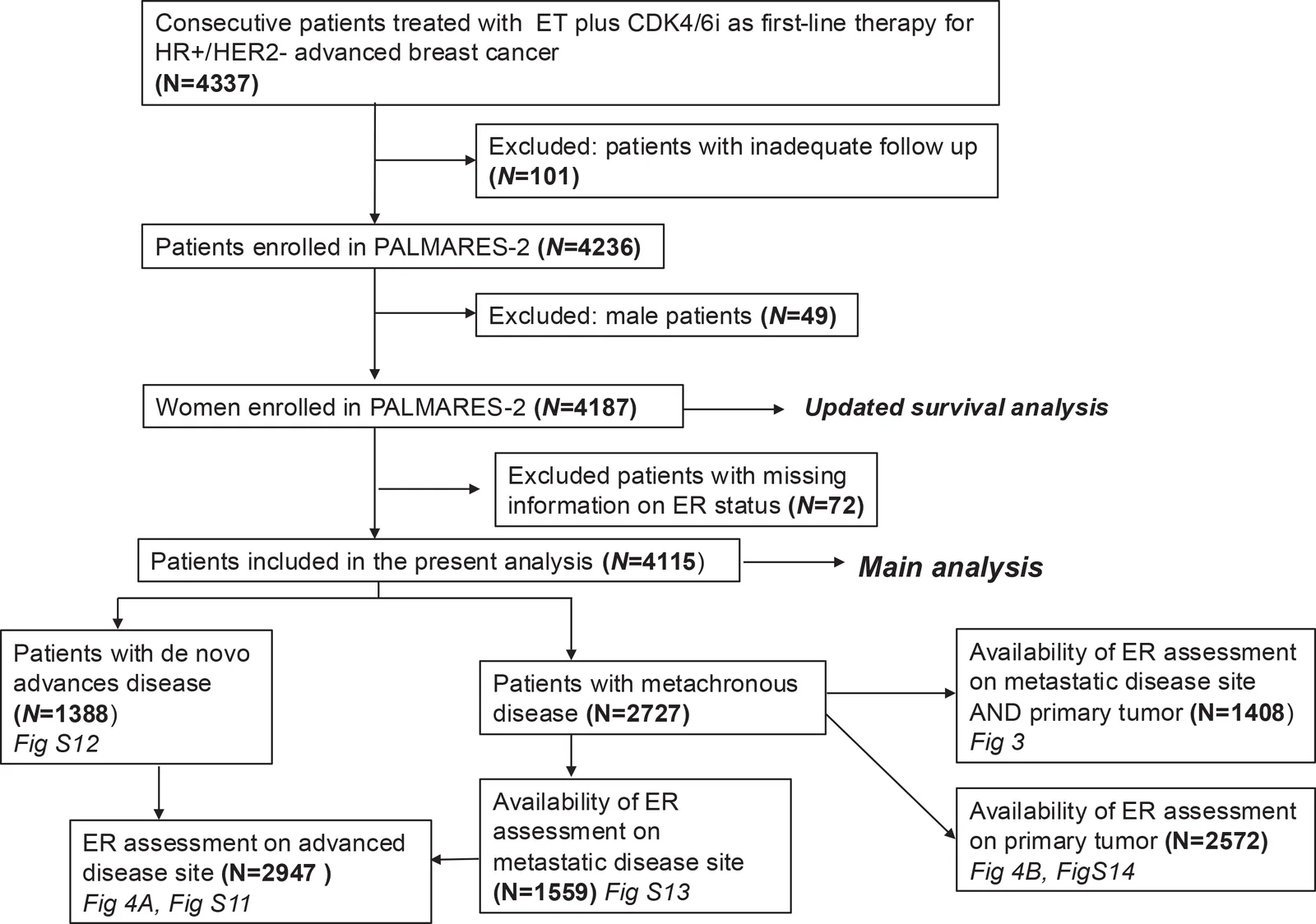
**Study flow-chart**.

### PALMARES-2 eligibility criteria

Eligible patients were pre- or post-menopausal women or men aged ≥18 years, with a diagnosis of advanced breast cancer (aBC), defined as unresectable locally advanced or metastatic disease, and with HR+/HER2−aBC, defined as at least 1% ER and/or PgR positivity at immunohistochemistry (IHC); HER2 negativity was defined on the basis of an IHC score of 0, 1+, or 2+ with absence of gene amplification at *in situ* hybridisation (ISH) analysis. Patients were required to have received palbociclib, ribociclib, or abemaciclib in combination with endocrine therapy as first-line treatment for HR+/HER2− aBC. Patients were excluded if they had less than 3 months of follow-up from CDK4/6i initiation to the date of data cut-off, if they were treated with a CDK4/6i as monotherapy, or if they were treated with a CDK4/6i as adjuvant treatment for localised disease.

To be included in the analysis evaluating the association between ERα expression and outcomes, patients were additionally required to have the availability of quantitative ERα expression data in tumour pathology reports (see “Procedures” for details); patients with only qualitative ERα assessment were excluded from this sub-analysis.

### Study objectives and clinical outcomes

The primary objective of this pre-planned analysis of the PALMARES-2 study was to compare real-world Progression-Free Survival (rwPFS) during first-line ET plus CDK4/6i across predefined ERα-expression groups (very-high (ER-VH, ≥90%), high (ER-H, 50–89%), and low-intermediate (ER-LI, 1–49%)) based on ERα quantification by IHC in the most recently available tumour biopsy specimen.

Secondary objectives were: i) to compare real-world time-to-chemotherapy (rwTTC) in different ERα expression groups; ii) to describe changes in ERα expression in primary archival tumours vs. matched metastatic tumour specimens; iii) to investigate the association between ERα expression, as assessed at different timepoints during the disease course, and rwPFS/rwTTC; iv) to compare rwPFS and rwTTC in patients with low vs. high PgR expression in tumour cells; v) to assess changes in PgR expression during the course of the disease, and their association with treatment effectiveness; vi) to evaluate rwPFS, rwTTC in different combined ERα/PgR expression groups; vii) to compare rwPFS and rwTTC of palbociclib, ribociclib and abemaciclib in the whole study cohort, to confirm previously published data from the first study data cut-off,^29^ as well as across different ERα expression groups. As exploratory objective, we evaluated Overall Survival (OS) in different ERα- and combined ERα/PgR expression groups.

rwPFS was defined as the time between first-line ET + CDK4/6i initiation and the detection of disease progression, as evaluated according to radiological, clinical, or biochemical criteria by treating physicians, or patient death, whichever occurred first. rwTTC was defined as the time between first-line treatment start and the initiation of the first line of chemotherapy for metastatic disease or patient death, whichever occurred first. OS was defined as the time between first-line treatment start and patient death for any cause.

### Procedures

The PALMARES-2 study involves the collection of clinical real-world data (RWD), medical images and tumour samples from consecutive patients enrolled in the study. For this reason, no additional procedures other than those performed as for clinical practice are required for the study purposes. Data are collected locally by each participating centre, either from electronic or from paper medical records, and then reported on the study electronic Case Report Form (eCRF) platform.

Quantitative ERα and PgR expression was retrospectively retrieved from tumour pathology reports, and HR status was defined according to the ASCO/CAP guidelines.^4^ In detail, HR + definition was based on the presence of at least 1% of tumour nuclei staining positive for ERα or PgR at IHC analysis.^4^ HER2 negativity was defined in the presence of an IHC score of 0, 1+, or 2+ in the absence of *ERBB2* gene amplification at *in situ* hybridisation (ISH) analysis. In the main analysis of this study, ERα and PgR expression was defined based on immunohistochemistry (IHC) assessment performed in tumour samples collected at the time of advanced disease diagnosis, if available; otherwise, ERα/PgR status was assessed in the archival primary tumour specimen. Different sensitivity analyses were performed: a) in the subgroup of patients with available quantitative ERα expression, as assessed in tumour tissues collected at advanced disease diagnosis, which could be a diagnostic biopsy of metastatic site, or a breast tissue specimen in patients diagnosed with *de novo* advanced disease; b) considering ERα expression assessed in the archival primary tumour specimen.

### Definition of ERα and PgR expression groups

To investigate the prognostic value of ERα expression in the perspective of clinical usability, patients were assigned to three different cohorts on the basis of intratumour ERα expression: ER-VH, ≥90%, ER-H, 50–89%, ER-LI, 1–49%. The 90% cut-off corresponds to the median and the mode ERα expression value in our cohort (Figure S1), while the 50% cutoff has been previously used to define tumours with “intermediate” ERα expression.^11^ Since only 15 (0.4%) patients with ER-low tumours (quantitative ERα expression in the 1–9% range) were included in this analysis, and since these patients had similar clinical characteristics when compared to patients with tumours showing ERα expression in the 10–49% range, we included these patients in the same ER-LI group (Table S2, Figure S2).

PgR expression was dichotomised with a 20% cut-off of, which is a well-established prognostic value in early-stage HR+/HER2− breast cancer,^30^ and it is widely used as an IHC-based surrogate to distinguish luminal A from luminal B subtypes.

### Ethics

The PALMARES-2 study was conducted in accordance with the Declaration of Helsinki. Ethics approval was obtained from the Ethics Committee of the Study Sponsor, Fondazione IRCCS Istituto Nazionale dei Tumori di Milan (INT 101/23) and subsequently adopted by all the other participating centres. For all participants who were alive at the time of study conduction, a dedicated written consent was obtained. For patients included retrospectively in the study, and who were not alive at the time of study initiation, the Ethics Committee authorised the collection and analysis of data from patients who were not alive when the study was conducted and who already authorised the use of data collected as per clinical practice for research purposes.

The study was registered with the ClinicalTrials.gov code NCT06805812.

### Statistical analysis

Descriptive statistics was used to summarise patient characteristics. Medians and interquartile ranges (IQRs) were reported for continuous variables, while categorical variables were summarised as percentages of available data. Non-normality of continuous variables was confirmed by Shapiro–Wilk testing and visual inspection, supporting the use of non-parametric descriptors and tests. To study differences, in terms of clinical features, between different groups, the Kruskal–Wallis and the Chi-square tests were used for continuous variables and categorical variables, respectively. Follow-up times were estimated through the reverse Kaplan–Meier method. The Kaplan–Meier method was used to estimate rwPFS, rwTTC and OS probabilities in the overall population and/or across ERα and/or PgR expression groups. Univariate Cox proportional hazards models were used to estimate hazard ratios (HRs) and 95% confidence intervals (CIs) for rwPFS and rwTTC according to ERα expression category (ERα <50%, ERα 50–89%, ERα ≥90%). To formally assess the association between increasing ER expression and outcomes, ER expression was also modelled as an ordinal variable (ERα <50%, ERα 50–89%, ERα ≥90%, coded as 1, 2, and 3, respectively) in univariate Cox proportional hazards models for rwPFS and rwTTC, and the p-value for the ordinal covariate was reported as the p value for trend (p-trend). Multivariable analysis was performed through Cox regression modelling to estimate the effects of clinically relevant covariates (i.e., age, menopausal status, ECOG Performance Status (PS), tumour histology, ERα, PgR, HER2 and Ki67 tumour expression, endocrine sensitivity/resistance^31^/*de novo* metastatic disease, and the presence of liver, lung or bone metastasis) on survival outcomes. To account for differences in follow-up time across treatment groups, as well as for temporal changes in clinical practice, that could be exacerbated by the continuous inclusion of patients over time, we included a binary stratification factor based on first-line treatment initiation (<2022 vs. ≥ 2022). For the comparative effectiveness analysis of CDK4/6i, we additionally performed a sensitivity analysis stratifying Cox models by year of prescription as a continuous variable, to more finely capture calendar-time trends relevant to comparing agents introduced sequentially into practice. To evaluate whether the addition of PgR to ERα improved model discrimination and prognostic classification, we compared nested univariable and multivariable Cox models with and without PgR using the likelihood-ratio test, Harrell's C-index (with formal comparison per Kang et al.^32^), and category-free net reclassification improvement (NRI) and integrated discrimination improvement (IDI) at 12 months, using the survIDINRI R package. To explore the association between ERα expression as a continuous variable and clinical outcomes, we performed a sliding window analysis (STEPP framework^33^) estimating 12-month rwPFS and rwTTC probability across overlapping subpopulations defined by continuous ERα expression (window size ∼2%, 50% overlap). Additionally, continuous ERα expression was modelled using a restricted cubic spline (4 knots) within a multivariable Cox model adjusted for the same covariates of the primary analysis.

In addition to multivariable Cox regression models, we used the Inverse Probability of Treatment Weighting (IPTW) method to balance clinically relevant variables in the overall patient cohort, as well as in different ERα subgroups, for treatment effectiveness comparisons (abemaciclib vs. ribociclib vs. palbociclib). In detail, using the R package ipw, we estimated a multinomial logistic regression propensity score model for treatment group membership (palbociclib, ribociclib, or abemaciclib), conditional on the same patient- and tumour-related covariates included in the multivariable Cox regression models (age, menopausal status, ECOG PS, tumour histology, ER, PgR, HER2 and Ki67 expression, endocrine sensitivity/resistance or *de novo* metastatic disease, and presence of liver, lung, or bone metastases). Stabilised inverse probability weights were then derived from this model and applied to generate a weighted pseudo-population.

All covariates included in the present analysis have missing values in less or equal than 5% of patients; they were imputed using the median or the mode value (for numerical or categorical variables, respectively). We also performed sensitivity analyses, such as a complete case analysis and a multiple imputation analysis employing the mice R package (m = 20 imputed datasets; predictive mean matching for continuous variables, logistic regression for binary variables, and polytomous/ordinal regression for categorical variables) to assess the robustness of our results to the missing data handling approach. All statistical tests were two-tailed, with a p value (P) < 0.05 considered as statistically significant. All analyses were performed using R software version 4.4.1 (R Project for Statistical Computing; RRID SCR_001905) and RStudio (RRID SCR_000432) version 2024.09.0 + 375.

### Role of the funding source

PALMARES-2 is an academic investigator-initiated study partially supported by AstraZeneca/Daiichi Sankyo. The funders had no involvement in study design, data collection, data analyses, data interpretation, or the writing of the report.

## Results

### Study population and updated CDK4/6i effectiveness comparisons

At the second PALMARES-2 study data cut-off (January, 2025), 4187 women with HR+/HER2− aBC were enrolled (Fig. 1, Table S3). The most frequently used CDK4/6 inhibitor was ribociclib (n = 1651; 39%), followed by palbociclib (n = 1613; 39%) and abemaciclib (n = 923; 22%). Median age was 62 years (IQR 53–71). Most patients had an ECOG PS of 0 (n = 3270; 78%), Non-Special Type (NST) histology (n = 3110; 74%), and were postmenopausal (n = 3444; 82%). Two-thirds of patients (n = 2826; 67%) had endocrine-sensitive tumours, and 34% (n = 1403) of patients presented with *de novo* advanced disease. Median ERα, PgR, and Ki67 expression were 90% (IQR 85–95), 40% (IQR 5–80), and 20% (IQR 15–30), respectively. Bone was the most common site of metastasis (n = 2918; 70%), with bone-only disease in 25% (n = 1041) of patients; liver and lung metastases were present in 21% (n = 887) and 25% (n = 1057) of patients, respectively. At a median follow up of 40.3 months (IQR: 22.1–60.1), we detected 2522 rwPFS events and 2064 rwTTC events, with median rwPFS and rwTTC of 25.6 months (95% CI: 24.4–26.9) and 37.0 months (95% CI: 35.4–39.2), respectively (Figure S3A and B). Median follow-up was 55.7 months (IQR: 37.1–72.3), 33.9 months (IQR: 18.4–53.4) and 29.0 months (IQR: 17.0–44.6) in the palbociclib, ribociclib and abemaciclib cohorts, respectively. After adjustment for clinically relevant covariates, ribociclib and abemaciclib were associated with better rwPFS and rwTTC when compared to palbociclib (after IPTW balance (Figure S4; Table S4, Figure S5A and B): abemaciclib vs. palbociclib: rwPFS: HR = 0.86 (0.77−0.97), p = 0.014, rwTTC: HR = 0.85 (0.75−0.97), p = 0.017; Ribociclib vs. palbociclib: rwPFS: HR = 0.88 (0.8−0.97); p = 0.0089; rwTTC: HR = 0.85 (0.76−0.94); p = 0.002); in multivariable cox regression models (Figure S5C–F): abemaciclib vs. palbociclib: rwPFS: aHR = 0.83 (0.74−0.93), p < 0.001, rwTTC: aHR = 0.85 (0.75−0.96), p = 0.010; ribociclib vs. palbociclib: rwPFS: aHR = 0.87 (0.79−0.96); p = 0.004; rwTTC: HR = 0.84 (0.76−0.93); p = 0.001)). These results confirm and strengthen previous findings from a significantly smaller PALMARES-2 patient cohort.^29^^,^^34^ We did not observe significant rwPFS or rwTTC differences between abemaciclib and ribociclib (Figure S5A–F).

For the present analysis, which aims at evaluating the prognostic role of quantitative ERα expression in patients with HR+/HER2− aBC treated with first-line ET plus CDK4/6/i, we included only the 4115 (98.3%) patients with at least one tumour specimen with available quantitative ERα assessment (Fig. 1). When ERα expression was evaluated as a continuous variable, higher ERα expression levels in tumour cells were associated with better rwPFS and rwTTC (Figure S5C–F), an association further supported by sliding window and restricted cubic spline analyses (Figure S6).

### Patient characteristics according to ERα expression category

Of 4115 patients who were evaluated in this analysis, 157 (3.8%), 967 (23.5%) and 2991 (72.7%) patients had ER-LI, ER-H or ER-VH tumours, respectively. Patient and tumour characteristics were unbalanced across ERα expression groups (Table 1). In detail, patients with ER-LI tumours were younger (median age: 58 vs. 60 vs. 63 of patients with ER-LI vs. ER-H vs. ER-VH tumours, respectively; p < 0.001), while patients with ER-VH tumours were more commonly post-menopausal (84% (n = 2524) vs. 76% (n = 736) vs. 77% (n = 121) in patients with ER-VH vs. ER-H vs. ER-LI tumours, respectively; p < 0.001) and less likely to have an ECOG PS of 0 (77% (n = 2298) vs. 82% (n = 789) vs. 81% (n = 127) in patients with ER-VH vs. ER-H vs. ER-LI tumours, respectively; p = 0.018) (Table 1). Higher ERα expression correlated with higher PgR expression (median expression 50%, 30%, 2% in ER-VH, ER-H and ER-LI, respectively; p < 0.001), higher frequency of HER2-low tumours (57% (n = 1709) vs. 51% (n = 493) vs. 48% (n = 76); p < 0.001), higher percentage of endocrine-sensitive disease (69% (n = 2066) vs. 65% (n = 625) vs. 52% (n = 81); p < 0.001) and the presence of *de novo* metastatic disease at diagnosis (36% (n = 1066) vs. 31% (n = 297) vs. 16% (n = 25); p < 0.001) (Table 1).

**Table 1:** Patient characteristics according to ERα expression category, as assessed in the most recent available tumour sample.

Characteristic	Overall N = 4115	ERα ≥90% N = 2991	ERα 50–89% N = 967	ERα 1–49% N = 157	p-value
CDK4/6i drug					0.019
Palbociclib	1566 (38%)	1130 (38%)	391 (40%)	45 (29%)	
Abemaciclib	918 (22%)	665 (22%)	204 (21%)	49 (31%)	
Ribociclib	1631 (40%)	1196 (40%)	372 (38%)	63 (40%)	
Age (years; median, IQR)	62 (53, 71)	63 (54, 72)	60 (50, 70)	58 (51, 68)	<0.001^a^
PS_ECOG					0.018
0	3214 (78%)	2298 (77%)	789 (82%)	127 (81%)	
1	767 (19%)	588 (20%)	151 (16%)	28 (18%)	
≥2	134 (3.3%)	105 (3.5%)	27 (2.8%)	2 (1.3%)	
Histology					0.093
NST	3073 (75%)	2236 (75%)	724 (75%)	113 (72%)	
ILC	770 (19%)	543 (18%)	189 (20%)	38 (24%)	
Other	272 (6.6%)	212 (7.1%)	54 (5.6%)	6 (3.8%)	
PgR expression (%, median, IQR)	40 (5, 80)	50 (5, 90)	30 (1, 70)	2 (0, 20)	<0.001^a^
Ki67 expression (%, median, IQR)	20 (15, 30)	20 (15, 30)	20 (15, 30)	20 (15, 35)	0.2^a^
HER2 expression					<0.001
0	1837 (45%)	1282 (43%)	474 (49%)	81 (52%)	
low	2278 (55%)	1709 (57%)	493 (51%)	76 (48%)	
De novo aBC	1388 (34%)	1066 (36%)	297 (31%)	25 (16%)	<0.001
Menopausal status					<0.001
Postmenopausal	3381 (82%)	2524 (84%)	736 (76%)	121 (77%)	
Premenopausal	734 (18%)	467 (16%)	231 (24%)	36 (23%)	
Liver metasteses	878 (21%)	644 (22%)	189 (20%)	45 (29%)	0.031
Lung metasteses	1040 (25%)	789 (26%)	212 (22%)	39 (25%)	0.021
Bone metastases	2860 (70%)	2076 (69%)	682 (71%)	102 (65%)	0.4
Bone-only disease	1013 (25%)	704 (24%)	272 (28%)	37 (24%)	0.015
Endocrine resistance					<0.001
Sensitive	2772 (67%)	2066 (69%)	625 (65%)	81 (52%)	
Resistant	1343 (33%)	925 (31%)	342 (35%)	76 (48%)	
Endocrine treatment					<0.001
Aromatase inhibitors	2830 (69%)	2113 (71%)	634 (66%)	83 (53%)	
Fulvestrant	1285 (31%)	878 (29%)	333 (34%)	74 (47%)	
Luminal B-like^b^	2343 (57%)	1710 (57%)	535 (55%)	98 (62%)	0.2

Data are presented as n (%) except where otherwise noted. The p value of the chi squared test is indicated in the right column of the table, except where otherwise noted. The p value of the test is indicated in bold numbers when statistically significant. p-values were calculated excluding unknown values.

Abbreviations: CDK4/6i, cyclin-dependent kinase 4/6 inhibitors; ECOG PS, Eastern Cooperative Oncology Group Performance Status; ER, Oestrogen Receptor; HER2, Human Epidermal growth factor Receptor 2; ILC, Invasive Lobular Carcinoma; IQR, InterQuartile Range; NST, Non-Special Type; PgR, Progesterone Receptor.

a Kruskal Wallis test.

b Defined as PgR expression <1% and/or Ki67 > 20%.

The distribution of metastatic sites also differed according to ERα expression; patients with ER-VH were more likely to have lung metastases and less likely to have bone-only metastases when compared to patients with ER-H disease (26% (n = 789) vs. 22% (n = 212), respectively, p = 0.021 and 24% (n = 704) vs. 28% (n = 272), respectively, p = 0.015); on the other hand, patients with ER-LI tumours were more likely to have liver metastases when compared to patients with ER-H tumours (29% (n = 45) vs. 20% (n = 189), p = 0.031) (Table 1).

### Association between tumour ERα expression and patient outcomes

We compared clinical outcomes in ER-LI, ER-H and ER-VH subgroups. Median rwPFS was longer in patients with ER-H vs. ER-LI tumours (20.5 vs. 10.6 months, respectively; adjusted Hazard Ratio (aHR): 0.73, p = 0.002), and even longer in patients with ER-VH vs. ER-LI tumours (28.5 vs. 10.6 months, respectively; aHR: 0.54, p < 0.001) (Fig. 2) (p-trend <0.001). These findings were confirmed in multivariable models adjusted for clinically relevant variables (Fig. 2B) and remained robust under alternative missing-data handling approaches, including complete case analysis and multiple imputation (Figure S7A and B).

**Fig. 2:**
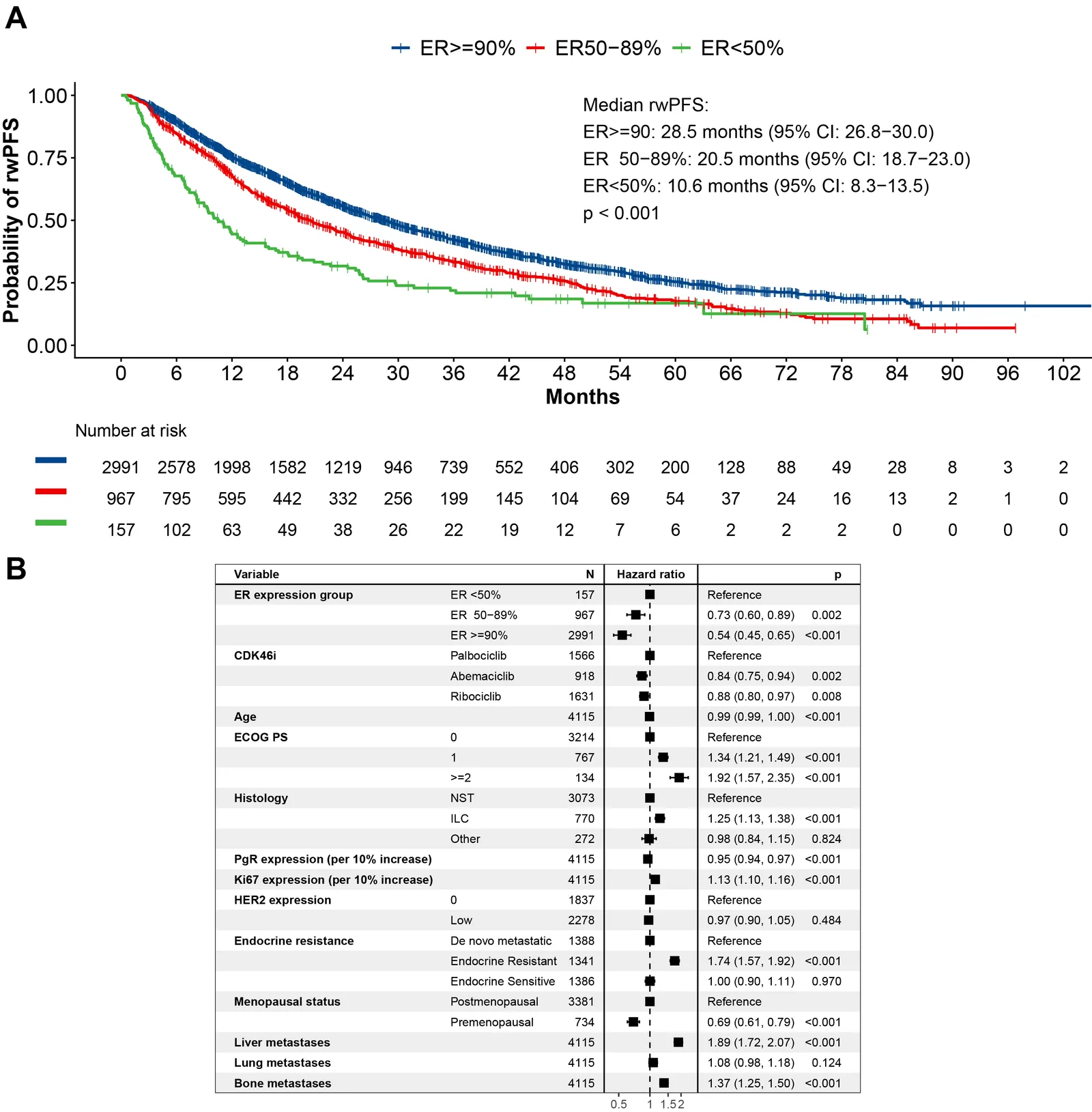
**Real-world progression free survival according to intratumour ERα expression.** Kaplan Meier curves for rwPFS in different ERα groups (A) and Forest plot illustrating the results of multivariable Cox regression model (B) in the whole PALMARES-2 patient cohort. *rwPFS, real-world progression-free survival; CDK4/6i, cyclin-dependent kinase 4/6 inhibitors; ECOG PS, Eastern Cooperative Oncology Group Performance Status; ERα, Oestrogen Receptor alpha; HER2, Human Epidermal growth factor Receptor 2; ILC, Invasive Lobular Carcinoma; NST, Non-Special Type; PgR, Progesterone Receptor. P values in panel A refer to the log-rank test; P values in panel B refer to the Wald test from the multivariable Cox regression model.*

Similar results were observed in the sub-cohorts of patients with endocrine-sensitive (Figure S8A and B) or endocrine-resistant (Figure S8C and D) tumours. Patients with ER-VH tumours also had significantly better rwTTC when compared to patients with ER-H and ER-LI tumours at both univariate (Figure S9A) (p-trend <0.001) and multivariable analysis (Figure S9B; Figure S7C and D). Finally, we performed an exploratory OS analysis, which showed a clear separation between the ER-VH subgroup (median OS: 70.8 months, 95% CI 66.8–76.5) and the other two categories, which largely overlapped (ER-H: 58.9 months, 95% CI 51.5–63.0; ER-LI: 58.5 months, 95% CI 42.6–NR; log-rank p < 0.001) likely as a result of small sample size and short follow-up time (Figure S10).

### ERα assessment on primary vs. metastatic tissue and its association with CDK4/6i effectiveness

In our cohort, matched primary and metastatic tumour specimens (available for n = 1408 patients) collected from patients who developed metachronous metastases showed significantly different ERα-category distributions (p < 0.001), with 49.8% (n = 701) of tumours changing ERα category at advanced disease diagnosis (Fig. 3).

**Fig. 3:**
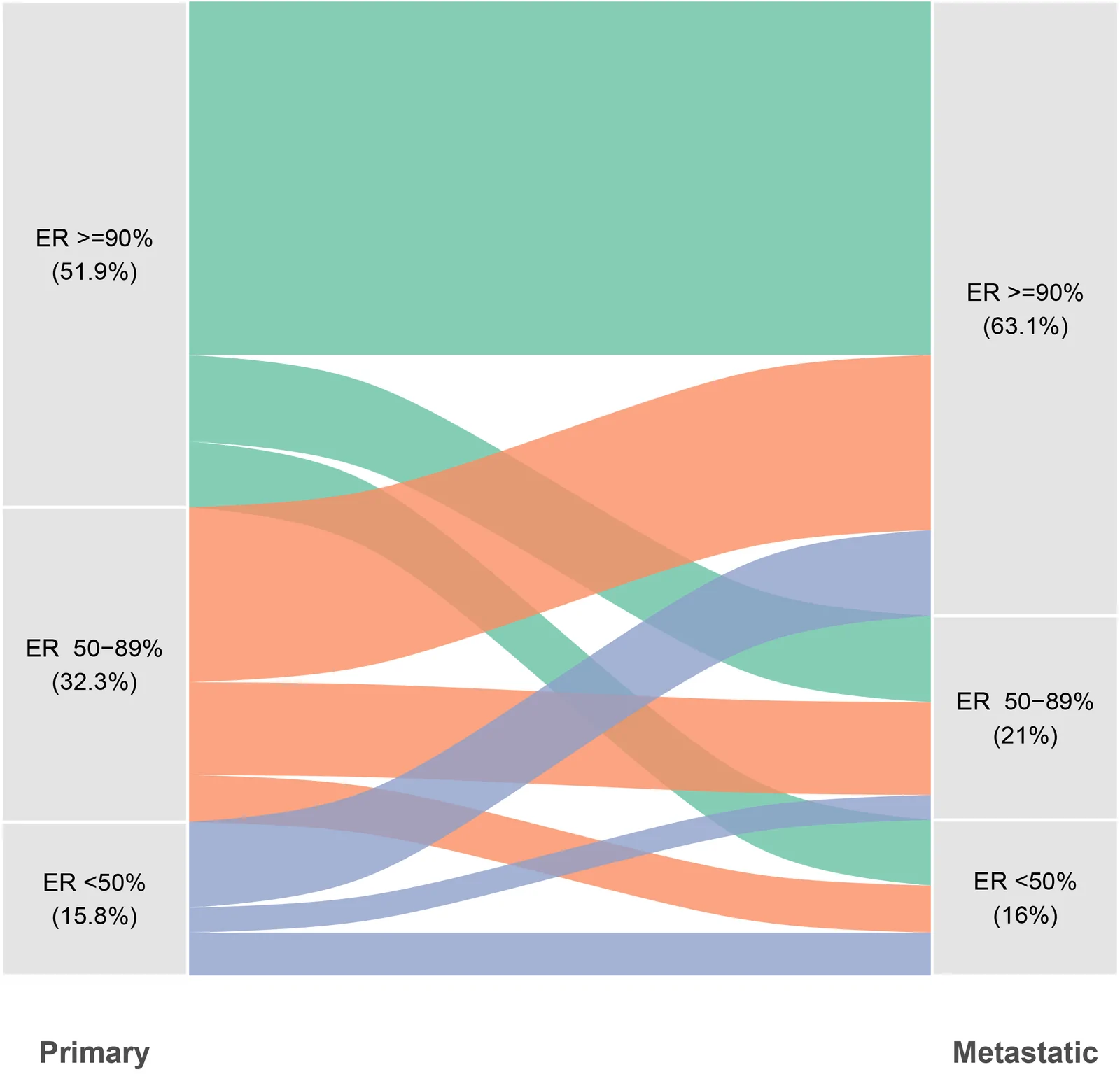
**Changes in ERα expression in primary vs. relapsed tumours in patients with metachronous tumours.** Alluvial plot showing ERα status (ER-VH, ER-H, ER-LI) assessed in the primary tumour (left) and in matched metastastic tumour specimen collected before first CDK4/6i plus ET treatment initiation (right) in the subgroup of 1408 patients in which both assessments had been performed.

Based on these results, we investigated the prognostic value of ERα expression levels, as assessed in the most recently available tumour specimen. To this aim, we performed a sensitivity analysis in 2947 patients for whom ERα expression was available in tumour samples collected at the diagnosis of advanced disease, i.e., within 3 months from initiation of first-line ET plus CDK4/6i (Table S5). In this sub-cohort, higher ERα expression was associated with better rwPFS (median rwPFS in ER-VH 31.4 months vs. 20.8 months in ER-H vs. 10.0 months in ER-LI tumours, p < 0.001) and rwTTC (median rwTTC 46.3 vs. 30.9 vs. 13.7 months in ER-VH, ER-H and ER-LI tumours, respectively p < 0.001), in line with the results of the primary analysis in the whole study cohort (Fig. 4A; Figure S11).

**Fig. 4:**
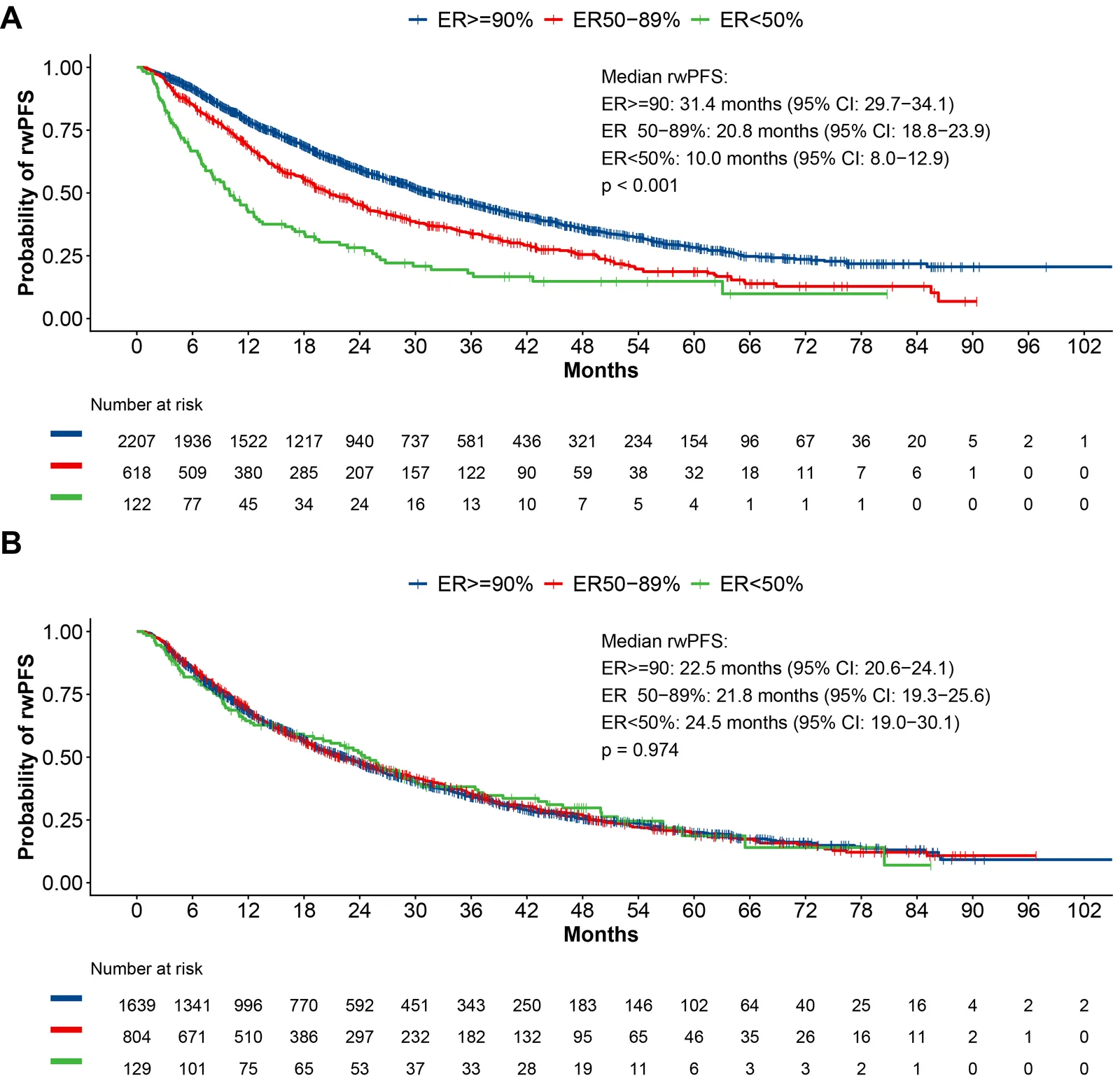
**Impact of ERα expression on real-world progression free survival according to the timing of ERα assessment.** Kaplan Meier curves for rwPFS according to ERα status (A) in the cohort of patients with ERα expression status assessed at the time of advanced disease diagnosis and (B) in patients with metachronous aBC and with ERα expression status assessed in the primary tumour specimen. *aBC, advanced breast cancer; ERα, oestrogen receptor alpha; rwPFS, real-world progression-free survival.* P values of the log-rank test are reported.

Patients with available ERα expression evaluation at the time of first-line ET + CDK4/6i initiation can have *de novo* advanced disease or metachronous metastases. Table S6 summarises disease sites where diagnostic biopsies were performed. Interestingly, ERα expression showed similar prognostic role in patients diagnosed with *de novo* advanced disease (Figure S12) and in those with metachronous metastases (Figure S13). By contrast, among patients with metachronous metastases, ERα status assessed in archival primary tumour specimens was not associated with rwPFS or rwTTC (Fig. 4B, Figure S14). In line with these results, an exploratory multivariable model showed a statistically significant interaction term between ERα expression and timing of ERα assessment in affecting rwPFS (recent biopsy vs. archival tumour specimen) (p for interaction <0.001).

Together, these data indicate that ERα expression is a reliable prognostic factor when assessed in the most recently available tumour specimen, which more reliably reflects tumour biology at ET + CDK4/6i initiation and, consequently, tumour sensitivity to this treatment.

### Association between combined ERα and PgR evaluation and CDK4/6i effectiveness

When evaluated as a continuous variable, higher PgR expression was associated with better rwPFS (Figure S5C) and rwTTC (Figure S5D).

Then, we assessed the prognostic value of dichotomised PgR expression according to a 20% cut-off. Patients with high tumour PgR expression (≥20%) had significantly better rwPFS and rwTTC when compared to patients with low PgR expression (<20%) (median rwPFS 30.7 vs. 19.2 months, respectively; HR: 0.65, 95% CI 0.60, 0.71 p < 0.001; median rwTTC 43.5 vs. 28.2 months, HR 0.63, 95% CI 0.58–0.69, p < 0.001) (Figure S15A and C). Multivariable analysis confirmed these findings (rwPFS: aHR 0.77, CI 0.71–0.84, p < 0.001 (Figure S15B); rwTTC: aHR 0.77, CI 0.71–0.85, CI, p < 0.001 (Figure S15D)).

Similar to ERα expression, PgR expression also showed substantial discordance between matched primary and metastatic tumour specimens, with a category shift observed in 41.2% (n = 574) of patients, mostly from PgR-high to PgR-low biology (35%, n = 487) (Figure S16). The prognostic value of PgR was more pronounced when PgR expression was evaluated in tumour specimens collected at the time of metastatic disease diagnosis (rwPFS 36.1 vs. 20.2 months in PgR-high vs. PgR-low tumours, p < 0.001) (Figure S17A–F). PgR expression evaluated on archival primary tumour specimens from patients who subsequently developed metachonous metastases retained an association with first-line ET + CDK4/6i effectiveness, even though the difference was numerically smaller (median rwPFS: 24.4 months in PgR-high vs. 18.3 months in PgR-low tumours, p < 0.001) (Figure S17G and H).

Finally, we investigated whether combining ERα and PgR expression adds prognostic value to the one of single biomarkers. The addition of PgR to a cox model for rwPFS including ERα significantly improved model fit (likelihood-ratio χ^2^ = 97.0, df = 3, p < 0.001) and improved discrimination, with Harrell's C-index increasing from 0.540 to 0.569 (p = 0.006) in univariate analysis. In the multivariable model, the addition of PgR similarly improved model fit (likelihood-ratio χ^2^ = 43.6, df = 3, p < 0.001) and modestly improved discrimination, with Harrell's C-index increasing from 0.665 to 0.670 (p = 0.003) and a category-free NRI of 19.2% (95% CI 11.6–25.2%, p < 0.001) and IDI of 0.008 (95% CI 0.004–0.014, p < 0.001) at 12 months. The clinical characteristics of patient cohorts identified according to ERα and PgR expression are detailed in Table S7. Across all ERα categories, high PgR expression was associated with better rwPFS and rwTTC (log rank p < 0.001, p-trend <0.001) (Fig. 5, Figure S18A). Patients with ER-VH and PgR-high tumours had the longest rwPFS and rwTTC (median rwPFS 32.4 months, 95% CI 30.2–34.9; median rwTTC: 46.4 months 42.6–50.0), while the 113 patients with ER-LI and PgR-low tumours showed significantly worse rwPFS and rwTTC outcomes (with 91 rwPFS and 78 rwTTC events, we observed a median rwPFS of 8.3 months, 95% CI 6.9–12.0; a median rwTTC: 13.1 months, 95% CI 9.14–16.4; a rwPFS rate at 3 months of 84.1%; 95% CI 0.78–0.91; a rwPFS rate at 4 months of 73.3%, 95% 0.66–0.82) (Fig. 5). Patients with intermediate ERα and/or PgR expression showed intermediate clinical outcomes (Fig. 5). As for OS, we observed a similar pattern, with patients with ER-VH and PgR-high tumours showing the longest OS (median OS: 74.4 months; 95% CI: 70.8−81.9) and patients with ER-LI and PgR-low tumours showing a significantly shorter survival (median OS 50.5 months; 95% CI: 36.7−NR); however, these findings should be interpreted with caution given data immaturity and the limited sample size of the smaller subgroups (Figure S18B).

**Fig. 5:**
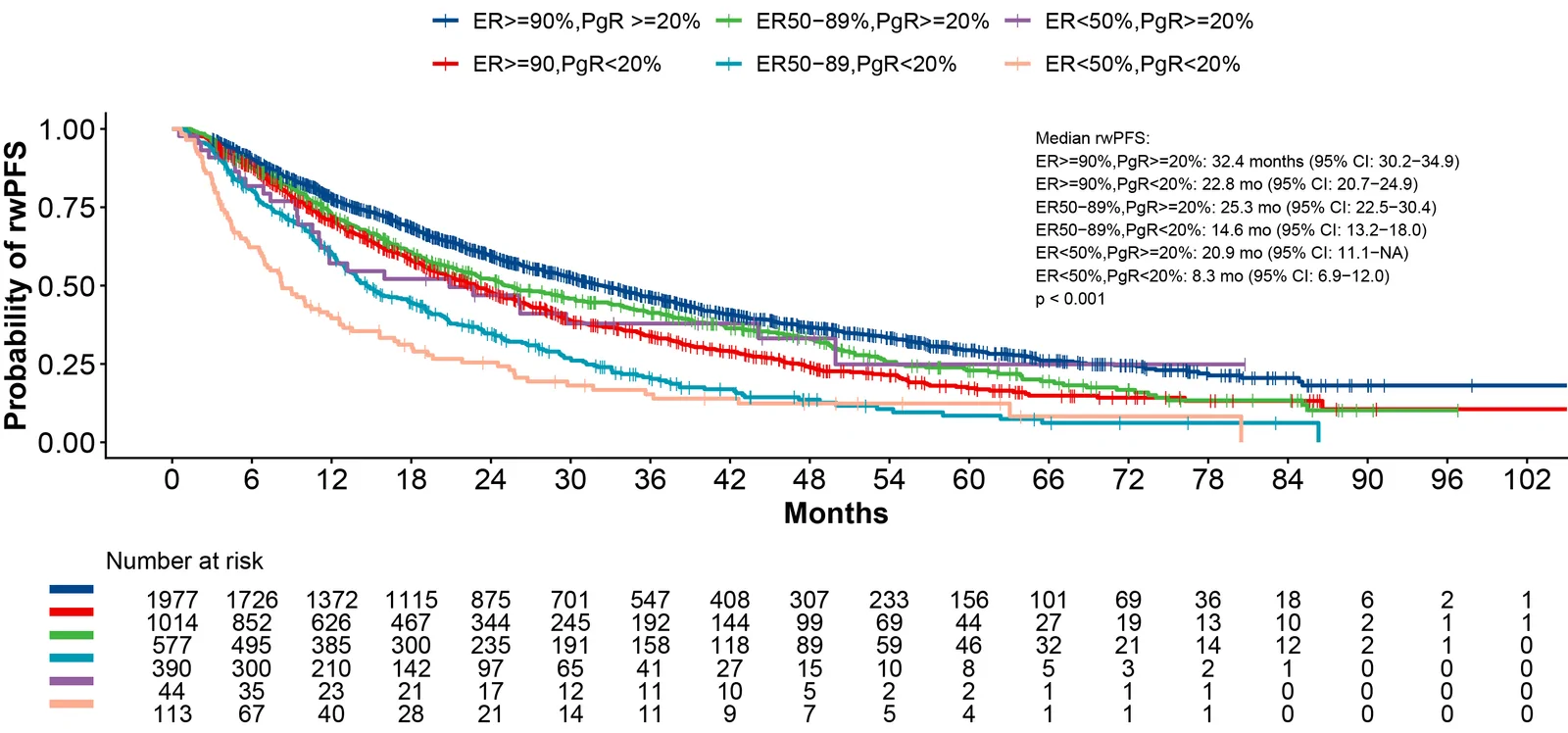
**Real-world progression free survival according to ERα and PgR expression.** Kaplan Meier curves for rwPFS according to ERα and PgR expression in the whole patient cohort. *rwPFS, real-world progression-free survival; ERα, Oestrogen Receptor alpha; PgR, Progesterone Receptor.* P value of the log-rank test is reported.

### Effectiveness comparison of palbociclib, ribociclib and abemaciclib according to ERα expression

In all ERα expression cohorts, patients treated with palbociclib had longer median follow-up (ER-LI: 48.4 months; ER-H: 57.3 months; ER-VH: 55.4 months) than patients treated with ribociclib (ER-LI: 39.6 months; ER-H: 32.8 months; ER-VH: 34.2 months) or abemaciclib (ER-LI: 34.3 months; ER-H: 31.5 months; ER-VH: 28.6 months). Since many clinical features were unbalanced among treatment groups (Table S8), we performed multivariable Cox regression models to compare the effectiveness of the three CDK4/6i.

In the ER-LI cohort, we found no significant rwPFS or rwTTC differences between palbociclib, ribociclib or abemaciclib (rwPFS: abemaciclib vs. palbociclib aHR = 0.76, 95% CI 0.43–1.33, p = 0.33; ribociclib vs. palbociclib aHR = 0.72, 95% CI 0.43–1.19, p = 0.20; abemaciclib vs. ribociclib aHR 1.05, 95% CI 0.62–1.79, p = 0.85; rwTTC: abemaciclib vs. palbociclib aHR = 1.03, 95% CI 0.57–1.85, p = 0.93; ribociclib vs. palbociclib aHR = 0.59, 95% CI 0.33–1.04, p = 0.07; abemaciclib vs. ribociclib aHR 1.74, 95% CI 0.99–3.05, p = 0.052) (Figure S19A and B). Among patients with ER-H tumours, abemaciclib was associated with longer rwPFS but not rwTTC when compared to palbociclib (rwPFS: aHR = 0.75, 95% CI 0.59–0.94, p = 0.01; rwTTC: aHR = 0.82, 95% CI 0.64−1.05, p = 0.12) and ribociclib (rwPFS: 0.77, 95% 0.61–0.98, p = 0.03; rwTTC: aHR 0.92, 0.72–1.19 p = 0.54). No significant differences in rwPFS or rwTTC were observed between ribociclib and palbociclib (rwPFS: aHR = 0.96, 95% CI 0.8−1.16, p = 0.70; rwTTC aHR = 0.89 95% CI 0.73−1.09 p = 0.27) (Figure S19C and D). In the ER-VH cohort, both abemaciclib and ribociclib showed better rwPFS and rwTTC when compared to palbociclib, with no significant differences between abemaciclib and ribociclib (rwPFS: abemaciclib vs. palbociclib aHR = 0.86, 95% CI 0.75−0.99; p = 0.03; ribociclib vs. palbociclib aHR = 0.86 95% CI 0.77−0.97, p = 0.01; abemaciclib vs. ribociclib aHR 1.00, 95% CI 0.87–1.15 p = 0.99; rwTTC: abemaciclib vs. palbociclib aHR = 0.85, 95% CI 0.73−0.99, p = 0.04; ribociclib vs. palbociclib aHR = 0.86, 95% CI 0.76−0.98, p = 0.02; abemaciclib vs. ribociclib aHR 0.99, 95% CI 0.85–1.16, p = 0.92) (Figure S19E and F).

Additionally, we performed a comparative analysis of treatment effectiveness across palbociclib, ribociclib, and abemaciclib after IPTW balance within ERα expression subgroups. However, following assessment of covariate balance after weighting, we found that adequate balance (SMD <0.1) was not achieved in the ER-LI subgroup, which comprises a limited number of patients (Figure S20; Tables S9–S11). After IPTW balancing, comparisons of rwPFS and rwTTC between palbociclib, ribociclib, and abemaciclib in the ER-H and ER-VH subgroups (Figure S21) were in line with the results obtained with the multivariable Cox model.

## Discussion

In the largest real-world study specifically designed to collect extensive data from patients with HR+/HER2− aBC treated with multiple lines of therapy, we show that ERα and PgR expression in the most recently available tumour specimen is strongly prognostic in patients treated with first-line ET + CDK4/6i.

Previous studies revealed the prognostic role of quantitative ERα or PgR assessment early-stage or advanced HR+/HER2− BC,^2^^,^^35^, ^36^, ^37^ including small retrospective cohorts of patients treated with ET + CDK4/6i.^38^, ^39^, ^40^, ^41^ Our study extends these findings by showing that ERα and PgR intratumour levels have independent prognostic significance in a large cohort of patients with HR+/HER2− aBC treated with first-line ET + CDK4/6i.

As regards ERα expression, we identified three groups of patients with remarkably different rwPFS and rwTTC depending on different ERα expression levels (1–49%, 50–89%, 90–100%). Although arbitrary, these ERα expression thresholds were chosen to facilitate their clinical applicability: the 90% cut-off reflects both the median and the most frequently observed ERα expression value in our cohort (Figure S1), while the 50% cut-off has been previously used to define tumours with intermediate ERα expression.^11^ Only a few patients (∼0.3%, n = 15) with ER-low tumours (ERα1–9%) were enrolled in the PALMARES-2 study (vs. 3–8% of unselected HR + BC cohorts^35^^,^^42^), which reflects Italian treatment practice favouring cytotoxic-based therapy over ET + CDK4/6i in this clinical context. As for PgR expression, the widely accepted 20% threshold surrogating luminal A vs. B subtypes^30^^,^^43^ distinguished tumours associated with significantly different outcomes, confirming the prognostic value of PgR expression in patients receiving first-line ET + CDK4/6i.

Interestingly, combining ERα and PgR expression remarkably improves patient outcome prediction, with important potential clinical implications: a) the excellent outcomes of patients with ER-H/VH and PgR-high tumours support first-line ET + CDK4/6i as the preferential treatment choice regardless of metastatic tumour sites, disease burden and patient ECOG PS; b) in patients with ER-H/VH, PgR-low or ER-LI, PgR-high tumours, ET + CDK4/6i remains the preferred first-line treatment choice, although closer clinical and radiological monitoring could be hypothesised to be of value, especially in the presence of adverse prognostic factors; c) finally, patients with ER-LI, PgR-low tumours showed very poor outcomes during first-line ET + CDK4/6i, suggesting that this subgroup could be prioritised for experimental treatments in the context of clinical trials, or to novel triple treatments, such as ET + CDK4/6i + inavolisib^44^ combination in case of *PIK3CA-*mutated tumours. In the Destiny-Breast04 and Destiny-Breast06 trials,^45^^,^^46^ trastuzumab-deruxtecan (T-DXd) resulted in median PFS of more than 10 months in patients with HER2-low and HER2-low/ultralow disease when used after at least two-three lines of systemic therapy. Given T-DXd efficacy in later therapy lines and the attrition associated with each treatment change,^47^^,^^48^ we believe that T-DXd warrants prospective investigation against first-line ET + CDK4/6i in the context of clinical trials in patients with ER-LI/PgR-low HR+/HER2-low/ultralow aBC.

A clinically relevant finding of this study is that the prognostic value of ERα expression was observed only when ERα was assessed in tumour samples collected immediately before ET + CDK4/6i initiation, but not in archival primary tumour specimens, likely reflecting biological evolution under the selective pressure of adjuvant ET.^35^^,^^49^^,^^50^ These data reinforce the importance of rebiopsing tumours at the diagnosis of metastatic disease, not only to confirm tumour biology (i.e., to exclude a switch from HR+/HER2− BC to TNBC or HER2+ BC), but also to obtain more precise and updated information on quantitative ERα and PgR expression.

In this study, we confirmed our previous findings^29^ that ribociclib and abemaciclib are more effective than palbociclib (Figure S5), now in a more than double cohort of patients with HR+/HER2− aBC, further strengthening the robustness of this real-world comparative-effectiveness signal. As with any non-randomised comparison, residual confounding cannot be fully excluded; however, this concern is mitigated by the large sample size, extensive covariate adjustment, and consistency with our previously published findings. These results are clinically relevant in the context of the ongoing debate on optimal CDK4/6i selection in routine practice and of discordant real-world evidence. The recently published P-VERIFY study^51^^,^^52^ reported similar effectiveness of palbociclib, ribociclib, and abemaciclib in more than 9000 patients with HR+/HER2− aBC.; however, when compared to PALMARES-2, P-VERIFY is limited by greater imbalance across treatment cohorts, shorter median follow-up, and more importantly, by limited availability of key covariates, including quantitative ERα/PgR expression, tumour histology, endocrine resistance, and liver metastases, for multivariable adjustment, which may partly explain the discrepant findings.

We also observed that ET + CDK4/6i effectiveness varies according to ERα expression: in ER-LI tumours, the three inhibitors showed comparable effectiveness; abemaciclib appeared more effective in ER-H tumours; abemaciclib and ribociclib outperformed palbociclib in ER-VH tumours. Given the exploratory nature of this subgroup analysis, these findings should be considered hypothesis-generating; however, if confirmed by future studies, these results may help clinicians select the most appropriate CDK4/6i after balancing efficacy, safety, drug–drug interactions, and patient preferences.

Main strengths of this study include: a) a large, representative real-world population; b) comprehensive clinical and biological data annotation, which enables robust adjustment for confounding factors; c) focus on easily assessable prognostic and predictive biomarkers. Notably, this real-world study demonstrates that combined evaluation of ERα and PgR evaluation identifies subgroups of patients with HR+/HER2− aBC characterised by markedly different outcomes under first-line ET + CDK4/6i. Main study limitations include (i) the retrospective design; (ii) arbitrary ERα thresholds; (iii) the lack of central ERα/PgR pathology review; (iv) the absence of NGS data, which precluded assessment of genomic alterations (e.g., ESR1, PIK3CA, BRCA1/2 mutations) as potential outcome modifiers; (v) the immaturity of OS data, particularly in smaller ER-LI subgroups, warranting cautious interpretation of these findings; (vi) race and ethnicity data were not systematically collected. Finally, comparisons across CDK4/6 inhibitors should be regarded as exploratory, especially in ER-LI subgroup, given the limited sample size.

In conclusion, this study was designed to determine whether ERα and PgR expression are independently associated with first-line ET + CDK4/6i effectiveness in a large real-world cohort of patients with HR+/HER2− aBC. We found that ERα expression, especially when assessed in a recent tumour biopsy and jointly evaluated with PgR, is a strong, independent biomarker of first line ET + CDK4/6i effectiveness in patients with HR+/HER2− aBC, able to indentify groups of patients with markedly different prognosis. If confirmed in prospective studies, this prognostic stratification could inform clinicians in adapting the frequency of clinical and radiological disease assessments on the basis of patient risk, and suggests the possibility of preferentially directing patients with particularly dismal prognosis, such as those with ER-LI PgR-low tumours, toward more aggressive or experimental therapies. Further studies are warranted to test whether treatment strategies tailored to quantitative hormone receptor expression improve outcomes in this population.

## Contributors

FL and CV conceived and designed the study. All authors enrolled patients and collected clinical data at their respective institutions. FL and LP curated and analysed the data. FL, MVD, and CV wrote the original draft of the manuscript. All other authors (LP, RC, PV, MGiu, GCu, AT, AB, RP, SC, ML, GR, BTa, MS, MGio, LG, IM, MP, AF, AZ, FP, AGe, NL, DS, OG, MD, CCa, GB, MLD, LC, VG, MB, AMe, GG, GArm, AI, GCo, FM, CCo, MVS, GArp, VC, PPB, AMa, EM, OP, SZ, MV, DC, RL, EC, CB, IL, DM, DA, MT, LV, BTi, GV, IP, AGu, GP) critically reviewed and edited the manuscript for important intellectual content. All authors contributed to data interpretation, and approved the final version of the manuscript for submission. FL, LP, and CV had full access to and verified the underlying raw data reported in this study; they are members of the academic study team and are not employed by AstraZeneca/Daiichi Sankyo. The PALMARES-2 group contributed patients and clinical data to this study but did not otherwise participate in study design, data analysis, or manuscript preparation.

## Data sharing statement

Aggregate and derived data supporting the findings, along with the analytic code, are provided within the manuscript and its Supplementary Materials. Owing to consent restrictions, individual-level participant data cannot be made publicly available. Deidentified individual-level data may be shared with qualified investigators for analyses that do not conflict with ongoing projects, upon request to the corresponding author (CV) and completion of a data-sharing agreement.

## Declaration of interests

FL: Speaker for Novartis, Pfizer, Eli-Lilly, Accademia di Medicina; advisory board for Novartis. LP: Invited speaker for Pfizer, Novartis, MSD. RC: honoraria from Novartis, Eli Lilly, Gilead, Pfizer, AstraZeneca, Daiichi Sankyo, Menarini, MSD, Pierre Fabre, Seagen, EllevaPharma, Pensiero Scientifico Editore, Gentili, Think2it, Roche; travel/accommodation support from Novartis, Gilead, AstraZeneca, Accord; participation on advisory boards for Novartis, Pfizer, Menarini, Gilead, AstraZeneca, Daiichi Sankyo, MSD, Eli Lilly, Roche. PV: Advisory role and honoraria from AstraZeneca, Daiichi-Sankyo, Eli-Lilly, Gilead, Incyte, Istituto Gentili, Novartis, Pfizer, Roche, Seagen, Teva; travel/accommodation support from Daiichi-Sankyo, Eli-Lilly, Novartis; research funding (to the institution) from Novartis and AstraZeneca. MGiu: consulting/advisory role and honoraria from AstraZeneca, Daiichi Sankyo, Eisai, Exact Sciences, Gilead, Gentili, Lilly, Menarini Stemline, MSD, Novartis, Pfizer, Roche, Seagen; travel/accommodation/expenses from AstraZeneca, Gilead, Lilly, Novartis, Pfizer; stock or stock options in ADC Therapeutics, Mersana, KPTI, Cardiff Oncology, Aldeyra Therapeutics, Bioatla, Clearside Biomedical. GCu: consulting fees from BMS, Roche, Pfizer, Novartis, Lilly, AstraZeneca, Daiichi Sankyo, Merck, Seagen, Ellipsis, Gilead, Menarini; grant from Merck; honoraria from Lilly, Pfizer, Relay, Gilead, Novartis, Menarini, Exact Science; travel/accommodation support from Daiichi Sankyo, Menarini. AT: consulting/advisory role and honoraria from Eli Lilly, Pfizer, Novartis, MSD, AstraZeneca, Gilead, Daiichi Sankyo, Menarini Stemline; travel grants from Novartis, AstraZeneca, Gilead, Daiichi Sankyo, Menarini Stemline. SC: Personal fees from Eli Lilly, Bayer, Novartis, Roche, Pfizer, and Menarini Stemline. ML: consulting/advisory role for Roche, Lilly, Novartis, AstraZeneca, Pfizer, Gilead, MSD, Pierre Fabre, Menarini, Nordic Pharma, Bayer, Ipsen, Daiichi Sankyo; speaker honoraria from Roche, Lilly, Novartis, Pfizer, AstraZeneca, Takeda, Ipsen, Sandoz, Libbs, Daiichi Sankyo, Gilead, Menarini; travel grants from Gilead, Roche, Daiichi Sankyo; research funding (institution) from Gilead; other financial interests as JCO Consultant Editor, Chair ESO Certificate of Competence in Breast Cancer. BTa: honoraria from Pfizer, Novartis, Eli Lilly, Gentili, Roche, Daiichi Sankyo. LG: Advisory/consultancy fee from AstraZeneca, Daiichi Sankyo, Eli Lilly, GlaxoSmithKline, Incyte, Novartis, Pfizer, MSD, Menarini Stemline, Abbvie; research funding from Menarini Silicon Biosystems. IM: consulting fees from Eli Lilly, Novartis, Pfizer, AstraZeneca, Daiichi Sankyo, Gilead, MSD, TEMA Sinergie, Exact Sciences, Menarini Stemline; participation as IDMC chair for the FAST-Forward Boost trial and IDMC member for the PARABLE trial (UK); unpaid Board of Directors role at ESTRO. MP: Travel support from Pfizer and Novartis. AZ: consulting fees and honoraria from Roche, Novartis, Lilly, Pfizer, AstraZeneca, Daiichi Sankyo, Gilead, Menarini Stemline, Exact Sciences, MSD, BeOne; participation on advisory boards for the same entities; travel/accommodation support from Roche, Novartis, Lilly, BeOne, Daiichi Sankyo, AstraZeneca, Ipsen. FP: Advisory boards for Pfizer, Novartis, Eli Lilly, Daiichi Sankyo, AstraZeneca; consultancy for Eli Lilly, Novartis, Pfizer, AstraZeneca, Daiichi Sankyo; honoraria as speaker from Novartis, Eli Lilly, Pfizer, AstraZeneca, Menarini Stemline, MSD, Accademia Nazionale di Medicina. NL: Grant from Eisai; speaker bureau from GSK; travel expenses from Gentili, Celgene, Pfizer; advisory role from Novartis, Celgene; advisory role/travel expenses from Pfizer; advisory board from MSD, Roche, Novartis, AstraZeneca, Daiichi Sankyo. OG: honoraria from Pfizer, AstraZeneca, Daiichi-Sankyo, Exact Sciences, Gilead, Gentili, MSD, Lilly; travel support from Novartis, Daiichi-Sankyo, MSD, Gilead, Ipsen. MD: Consulting/advisory and travel reimbursement from Roche, Novartis, Takeda, Lilly, Pierre Fabre, AstraZeneca, MSD, Seagen, Gilead, Daiichi Sankyo, Tomalab, Genetic, Pfizer, Menarini, Sophos, Istituto Gentili, Sanofi, Ipsen, GSK, Exact Science. GB: honoraria from Novartis, Eli Lilly, Pfizer, AstraZeneca, Daiichi Sankyo, Roche, Exact Sciences, Genetic Spa, Gilead, MSD, Pierre Fabre, Wave Pharma; travel/accommodation support from Roche, Gilead, AstraZeneca, Daiichi Sankyo, Novartis, Eli Lilly; participation on advisory boards for AstraZeneca, Daiichi Sankyo, Novartis, Gilead. VG: Honoraria for lectures/presentations/speaker bureaus/educational events from Eli Lilly, Daiichi Sankyo, GSK, Gilead, Novartis, Exact Sciences, Roche, AstraZeneca, Menarini Stemline, Zentiva, AbbVie, Pfizer; support for meeting/travel from Roche, AstraZeneca, Menarini Stemline; advisory boards for Eli Lilly, Daiichi Sankyo, Exact Sciences, Gilead, AstraZeneca, MSD, Novartis, Pfizer, Roche, Menarini Stemline; institutional patent interest related to HER2DX. MB: honoraria from Gilead, Novartis, AstraZeneca, Pfizer, Eli Lilly, Daiichi Sankyo, MSD; travel/accommodation support from Daiichi Sankyo, Gilead, Eli Lilly. GG: research grant to the institution from Fondazione Roche; advisory board fees from Pfizer, Eli Lilly; honoraria as invited speaker from Eli Lilly, Novartis; travel/accommodation support from Gilead, Eli Lilly, Pfizer, Novartis, Daiichi Sankyo, AstraZeneca. GArm: Honoraria as a speaker Pfizer, Lilly, Novartis. FM: fees for lectures/consultancy from Astra-Zeneca, Lilly, Novartis, Pfizer, Daiichi-Sankyo; grants to the Institution from Gilead; travel grants from Gilead, Lilly, Novartis. GArp: Honoraria from Roche, Pfizer, AstraZeneca, Novartis, Celgene, Eli Lilly, Amgen, Eisai. AMa: grants from Gilead Sciences; consulting fees from Menarini/Stemline, Roche, Lilly, AstraZeneca, Daiichi-Sankyo, Pfizer, StratifAI; travel/accommodation support from Menarini/Stemline, AstraZeneca, Lilly, Pfizer, GlaxoSmithKline, Daiichi-Sankyo. EM: honoraria from Lilly, Novartis, Ipsen, AstraZeneca, Gilead; travel/accommodation support from Roche, Pfizer, Lilly, Novartis, Gilead, AstraZeneca, Pierre Fabre, MSD Oncology; participation on advisory boards/DSMB for Pfizer, Exact Science, MSD Oncology, Daiichi Sankyo, AstraZeneca, Seagen, Ipsen. OP: travel support from Novartis. SZ: travel/accommodation support from Eli Lilly, Novartis. DC: participation on advisory boards for Novartis, Gentili. RL: consultant or advisor for Novartis, AstraZeneca, Daichii Sankyo; speaker honoraria from Novartis, Gentili; travel/accommodation expenses from Ipsen, Pfizer, Novartis, Menarini, Eli Lilly. CB: honoraria for presentation of a clinical case from Novartis (webinar, April 2026). LV: Personal fees from Daiichi Sankyo, AstraZeneca, Novartis, Eli Lilly, Menarini Stemline, Exact Sciences. GV: Advisory board for Pfizer, Novartis; speaker for Daiichi Sankyo, Eli Lilly; support for meeting from Eli Lilly, Pfizer, AstraZeneca. GP: Personal fees from Foundation One, Illumina, Lilly. MVD: research grants to the institution from Roche and AstraZeneca; consulting fees from AstraZeneca, MSD, Pfizer, Roche; honoraria as invited speaker from AstraZeneca, Daiichi Sankyo, Eli Lilly, Exact Sciences, Menarini-Stemline, Novartis, Roche; travel/accommodation support from Daiichi Sankyo, Eli Lilly, Gilead, Roche; co-inventor on HER2DX patent applications; participation on advisory boards for AstraZeneca, Daiichi Sankyo, Eli Lilly, Gilead, MSD, Novartis. CV: Advisory boards for Pfizer, Novartis, Eli Lilly, Daiichi Sankyo, AstraZeneca, Menarini Stemline, Roche, Gilead; consultancy for Eli Lilly, Novartis; honoraria as speaker from Novartis, Eli Lilly, Pfizer, AstraZeneca, Menarini Stemline, MSD, Istituto Gentili; research grants to the institution from Roche and Daiichi/AstraZeneca. All remaining authors have declared no conflicts of interest.
