# Supplementary material for: Prognostic value of hormone receptor expression in patients with HR+/HER2− advanced BC treated with first-line ET+CDK4/6i: results from the multicentre observational, real-world PALMARES-2 study
Source: eClinicalMedicine. 2026 Sep 22;100:104242. doi: 10.1016/j.eclinm.2026.104242 (PMC13659900; doi:10.1016/j.eclinm.2026.104242)
Supplement: Supplementary file 1 [file mmc1.pdf]

SUPPLEMENTARY DATA

SUPPLEMENTARY METHODS
Participating centres
Procedures
Baseline features collected in the PALMARES-2 dataset used in the present analysis

SUPPLEMENTARY FIGURES
Supplementary Figure S1. ERα expression in the whole PALMARES-2 study cohort.
Supplementary Figure S2. rwPFS in patients with ERα-low vs. ERα-intermediate tumours.
Supplementary Figure S3. rwPFS and rwTTC in the whole PALMARES-2 study cohort.
Supplementary Figure S4. Love Plots showing covariate balance plot before and after IPTW balance of treatment groups (palbociclib, ribociclib, abemaciclib).
Supplementary Figure S5. rwPFS and rwTTC comparisons of palbociclib, ribociclib and abemaciclib.
Supplementary Figure S6. Association between continuous ERα expression and rwPFS and rwTTC.
Supplementary Figure S7. Sensitivity analyses employing alternative missing-data handling.
Supplementary Figure S8. Association between ERα expression and rwPFS according to tumour endocrine sensitivity or resistance.
Supplementary Figure S9. Association between ERα expression and rwTTC.
Supplementary Figure S10. Overall survival according to ERα expression category.
Supplementary Figure S11. Association between ER*α* expression at advanced disease diagnosis and rwTTC
Supplementary Figure S12. Prognostic value of ER*α* expression in patients with de novo metastatic disease.
Supplementary Figure S13. Prognostic value of ER*α* expression in relapsed tumour specimens from patients with metachronous metastases.
Supplementary Figure S14. Prognostic value of ER*α* expression in primary tumour specimens from patients with metachronous metastases.
Supplementary Figure S15. Real-world Progression Free Survival according to PgR expression.
Supplementary Figure S16. Changes in PgR expression in primary vs. relapses tumours
Supplementary Figure S17. Association between PgR expression and real-world Progression Free Survival according to the timing of assessment.
Supplementary Figure S18. Real-world Time To Chemotherapy and Overall Survival according to ERα and PgR expression.
Supplementary Figure S19. Adjusted rwPFS and rwTTC comparisons between palbociclib, ribociclib and abemaciclib in each ERa category.
Supplementary Figure S20. Love Plots showing covariate balance plot before and after IPTW balance of treatment groups (palbociclib, ribociclib, abemaciclib) in patients with (A) ER-LI, (B) ER-H, (C) ER-VH tumors.
Supplementary Figure S21. rwPFS and rwTTC comparisons between palbociclib, ribociclib and abemaciclib after IPTW in ER-H and ER-VH subgroups.

SUPPLEMENTARY TABLES
Supplementary Table S1. Baseline features collected within the PALMARES-2 study.
Supplementary Table S2. Characteristics of patients with tumours with ER-low (1-9%) expression vs those of patients with tumours with ER-intermediate expression (10-50%)
Supplementary Table S3. Clinical characteristics of women included at the PALMARES-2 second data cutoff
Supplementary Table S4. Unweighted and IPTW-weighted patient and tumor characteristics by CDK4/6 inhibitor group (palbociclib, ribociclib, abemaciclib), with corresponding SMDs before and after weighting.
Supplementary Table S5. Characteristic of patients with ERα expression status assessed at advanced disease diagnosis
Supplementary Table S6. Sites of biopsies (excluding primary tumour) performed at advanced disease diagnosis
Supplementary Table S7. Patient characteristics according to ER*α* and PgR expression category
Supplementary Table S8. Patient characteristics according to CDK4/6i treatment in each ER*α* expression group
Supplementary Table S9. Unweighted and IPTW-weighted patient and tumor characteristics by CDK4/6 inhibitor group, with corresponding SMDs before and after weighting in patients with ER-LI tumors
Supplementary Table S10. Unweighted and IPTW-weighted patient and tumor characteristics by CDK4/6 inhibitor group, with corresponding SMDs before and after weighting in patients with ER-H tumors
Supplementary Table 11. Unweighted and IPTW-weighted patient and tumor characteristics by CDK4/6 inhibitor group, with corresponding SMDs before and after weighting in patients with ER-VH tumors

SUPPLEMENTARY METHODS

Participating centres

PALMARES-2 (NCT06805812) is a retrospective-prospective, observational, real-world study enrolling consecutive patients with HR+/HER2- aBC who initiated first line treatment with endocrine therapy (ET) plus CDK4/6 inhibitors (CDK4/6i) after January 1st, 2016. The first PALMARES-2 data cut-off was performed on 31 January 2024, with 18 participating Italian centers, and included n=1982 patients[1]. The current, second cut-off reflects growth from both continued accrual at the original participating institutions and the addition of new institutions to the PALMARES-2 network and to date, 26 Italian institutions are participating in the study, spanning Northern, Central, and Southern Italy, and including both public and private, as well as university and non-university institutions, thus supporting the representativeness of the cohort with respect to real-world clinical practice in Italy.

Baseline features collected in the PALMARES-2 dataset used in the present analysis

All the following variables included in the present analysis were retrieved at the time of first-line ET+CDK4/6i initiation or before.

- Patient-related variables:
  1. Age.
  2. Performance Status: defined by Eastern Cooperative Oncology Group (ECOG) scale.
  3. Menopausal status: defined by the presence of amenorrhea for at least 12 months before ET plus CDK4/6i initiation, eventually confirmed through the quantification of blood estradiol and FSH concentration, if clinically indicated.
- Tumour-related variables:
  4. Endocrine sensitivity: detection of inoperable/metastatic tumour relapse occurring more than 12 months after the completion of adjuvant ET, or detection of *de novo* inoperable/metastatic disease at the time of BC diagnosis. Endocrine resistance: detection of inoperable/metastatic tumour relapse during the course of adjuvant ET, or within 12 months from the completion of adjuvant ET[2].
  5. De novo metastatic disease: defined by the occurrence of inoperable/metastatic disease without prior history of early HR+/HER2- BC.
  6. Histology: as defined by WHO classification for breast tumours and described on local pathologist report.

7. ERα expression in the most recent tumour specimen: percentage of tumour cells expressing ERα, as evaluated through IHC analysis in a tumour biopsy collected at the time of advanced disease diagnosis, if available; otherwise, ERα expression was assessed in the archival primary tumour specimen. If primary tumour was used for ERα evaluation, the assessment performed on surgical specimen was adopted if no preoperative treatment was administered; otherwise ERα expression was evaluated in primary tumour biopsy specimens.
8. ERα expression in primary archival tumour: in patients with metachronous advanced breast cancer, the percentage of tumour cells expressing ERα as evaluated through IHC analysis as assessed in the archival primary tumour specimen. The assessment performed on surgical specimen was adopted if no preoperative treatment was administered; otherwise ER expression was evaluated in primary tumour biopsy specimens.
9. ERα expression at advanced disease diagnosis: percentage of tumour cells expressing ERα as evaluated through IHC analysis in a biopsy obtained at advanced disease diagnosis. For patients with metachronous advanced breast cancer, samples were collected from metastatic sites, whereas in patients with *de novo* disease also the primary tumour could have been used
10. PgR expression: percentage of tumour cells expressing PgR, as evaluated through IHC analysis in a tumour biopsy collected at the time of advanced disease diagnosis, if available; otherwise, PgR expression was assessed in the archival primary tumour specimen. If primary tumour was used for PgR evaluation, the assessment performed on surgical specimen was adopted if no preoperative treatment was administered; otherwise PgR expression was evaluated in primary tumour biopsy specimens.
11. PgR expression in primary archival tumour: in patients with metachronous advanced breast cancer, the percentage of tumour cells expressing PgR as evaluated through IHC analysis as assessed in the archival primary tumour specimen. The assessment performed on surgical specimen was adopted if no preoperative treatment was administered; otherwise PgR expression was evaluated in primary tumour biopsy specimens.
12. PgR expression at advanced disease diagnosis: percentage of tumour cells expressing PgR as evaluated through IHC analysis in a biopsy obtained at advanced disease diagnosis. For patients with metachronous advanced breast cancer, samples were

collected from metastatic sites, whereas in patients with *de novo* disease also the primary tumour could have been used

13. HER2 expression: percentage of tumour cells expressing HER2, as evaluated through IHC analysis in a tumour biopsy collected at the time of advanced disease diagnosis, if available; otherwise, HER expression was assessed in the archival primary tumour specimen. If primary tumour was used for HER2 evaluation, the assessment performed on surgical specimen was adopted if no preoperative treatment was administered; otherwise HER2 expression was evaluated in primary tumour biopsy specimens.
14. Ki67 expression: percentage of tumour cells expressing Ki67, as evaluated through IHC analysis in a tumour biopsy collected at the time of advanced disease diagnosis, if available; otherwise, as assessed in the archival primary tumour specimen. If primary tumour was used for KI67 evaluation, the assessment performed on surgical specimen was adopted if no preoperative treatment was administered; otherwise Ki67 expression was evaluated in primary tumour biopsy specimens.
15. Grading: reported by local pathologist in a tumour biopsy collected at the time of advanced disease diagnosis, if available; otherwise, tumour grading was assessed in the archival primary tumour specimen. If the primary tumour specimen was used for grading evaluation, the assessment performed on surgical specimen was adopted if no preoperative treatment was administered; otherwise, tumour grading was evaluated in primary biopsy specimen.
16. Liver metastases.
17. Lung metastases.
18. Bone metastases.
19. Number of metastatic sites: number of metastatic organs (e.g., liver, lung) at the initiation of first line ET plus CDK4/6i therapy.
20. Metastatic site used for ER/PgR assessment

- Treatment-related variables:

21. CDK4/6i drug: type of CDK4/6i administered as first-line treatment (i.e., palbociclib, ribociclib, or abemaciclib).

References

1 Provenzano L, Dieci MV, Curigliano G, *et al.* Real-world effectiveness comparison of first-line palbociclib, ribociclib or abemaciclib plus endocrine therapy in advanced HR-positive/HER2-negative BC patients: results from the multicenter PALMARES-2 study. *Annals of Oncology* 2025; 36: 762–74.

2 Cardoso F, Paluch-Shimon S, Senkus E, *et al.* 5th ESO-ESMO international consensus guidelines for advanced breast cancer (ABC 5). *Annals of Oncology* 2020; 31: 1623–49.

SUPPLEMENTARY FIGURES

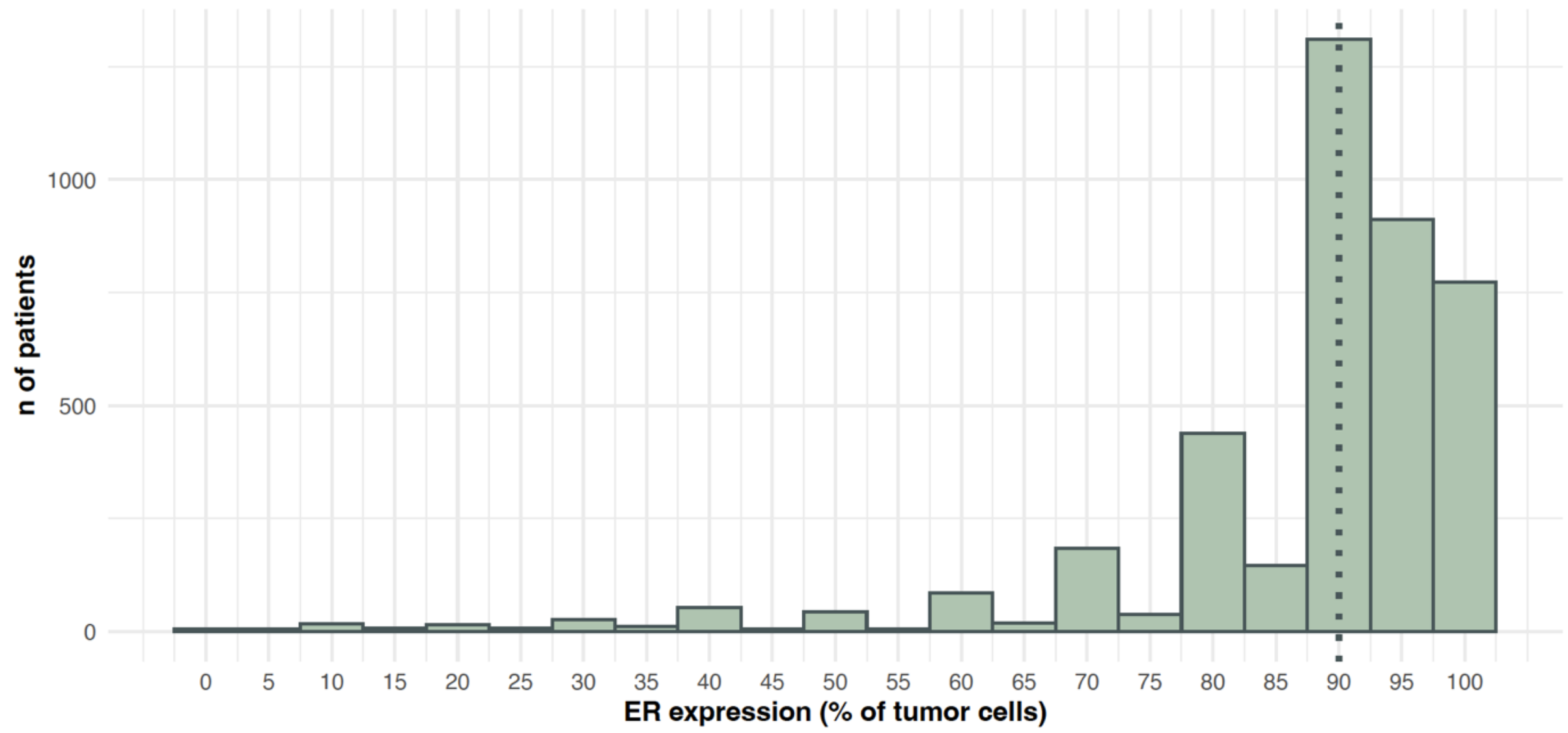

Supplementary Figure S1. ERα expression in the whole PALMARES-2 study cohort.
Bar chart illustrates the absolute number of patients with tumours showing the indicated percentage of ERα-positive cells. The dashed line indicates the median. *ERα: Oestrogen Receptor.*

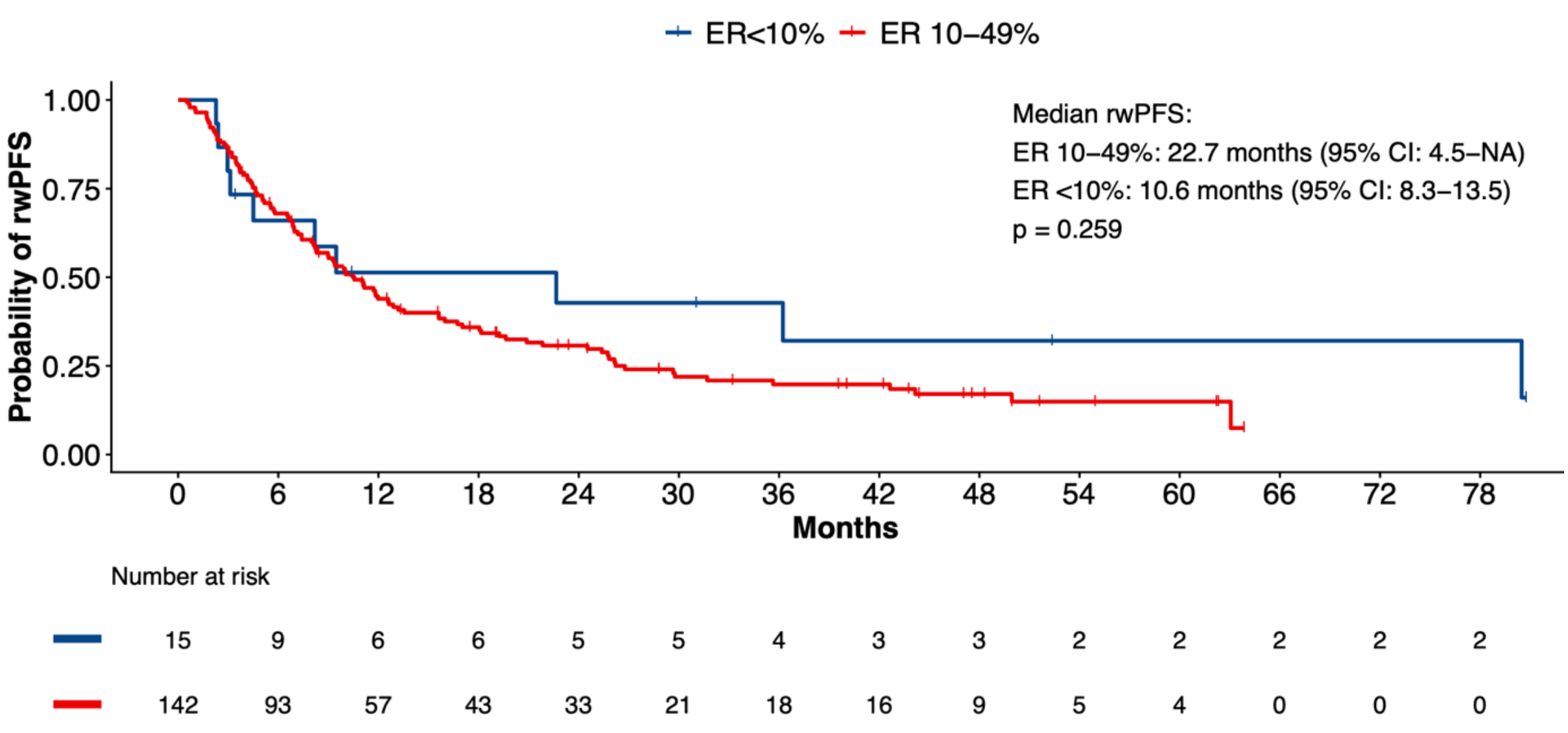

Supplementary Figure S2. rwPFS in patients with ERα-low vs. ERα-intermediate tumours.
Kaplan Meier curves comparing rwPFS in patients with low (1-9%) ERα-expressing tumours vs. patients with tumours expressing intermediate (10-49%) ERα levels. ERα: Oestrogen Receptor; rwPFS: real-world progression-free survival. P value of the log-rank test is reported.

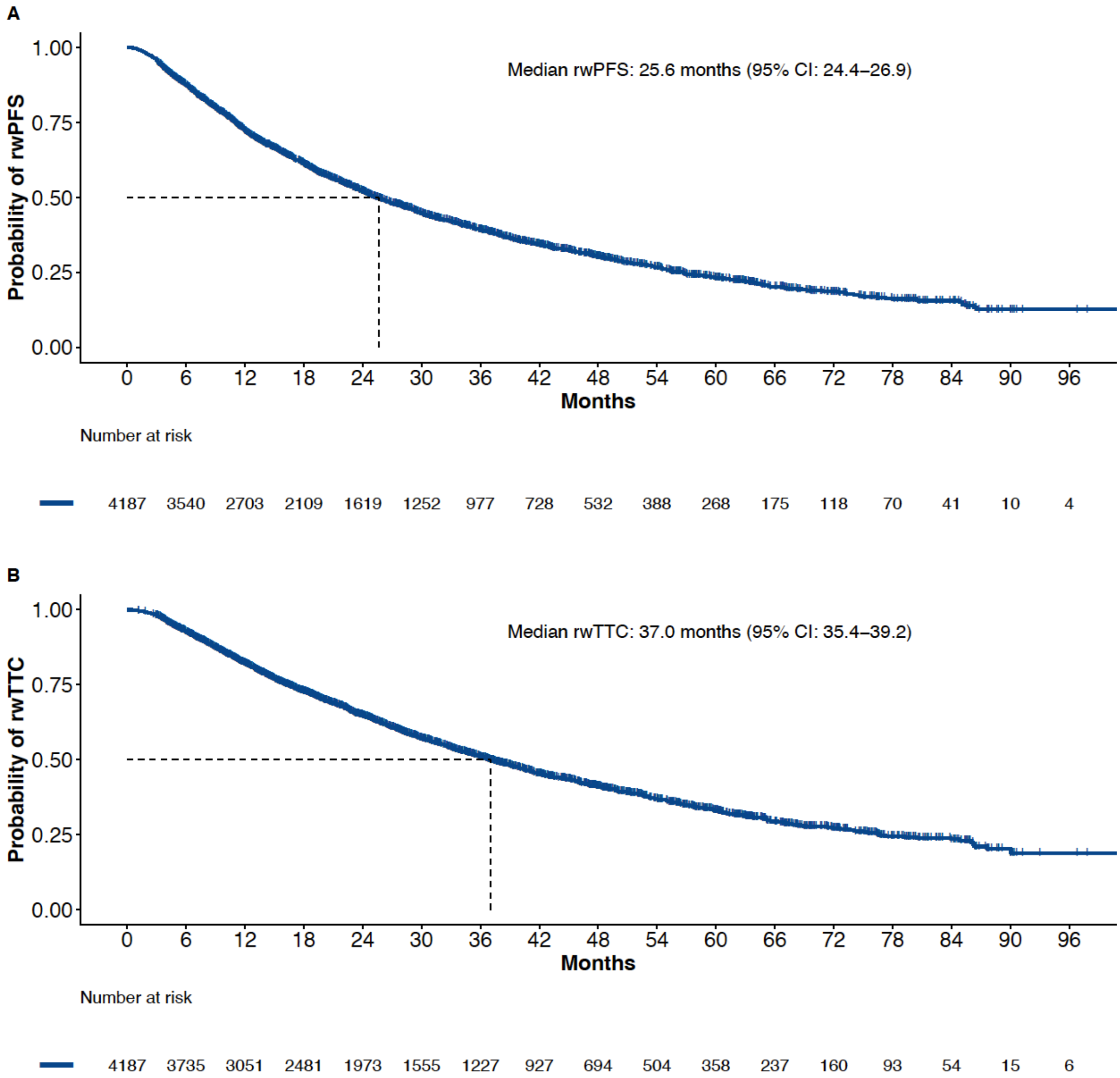

Supplementary Figure S3. rwPFS and rwTTC in the whole PALMARES-2 study cohort.

Kaplan Meier curves for rwPFS (A) and rwTTC (B) in the whole PALMARES 2 study cohort at the second study data-lock (January 2025) are shown. *rwPFS: real-world progression-free survival; rwTTC time to chemotherapy.*

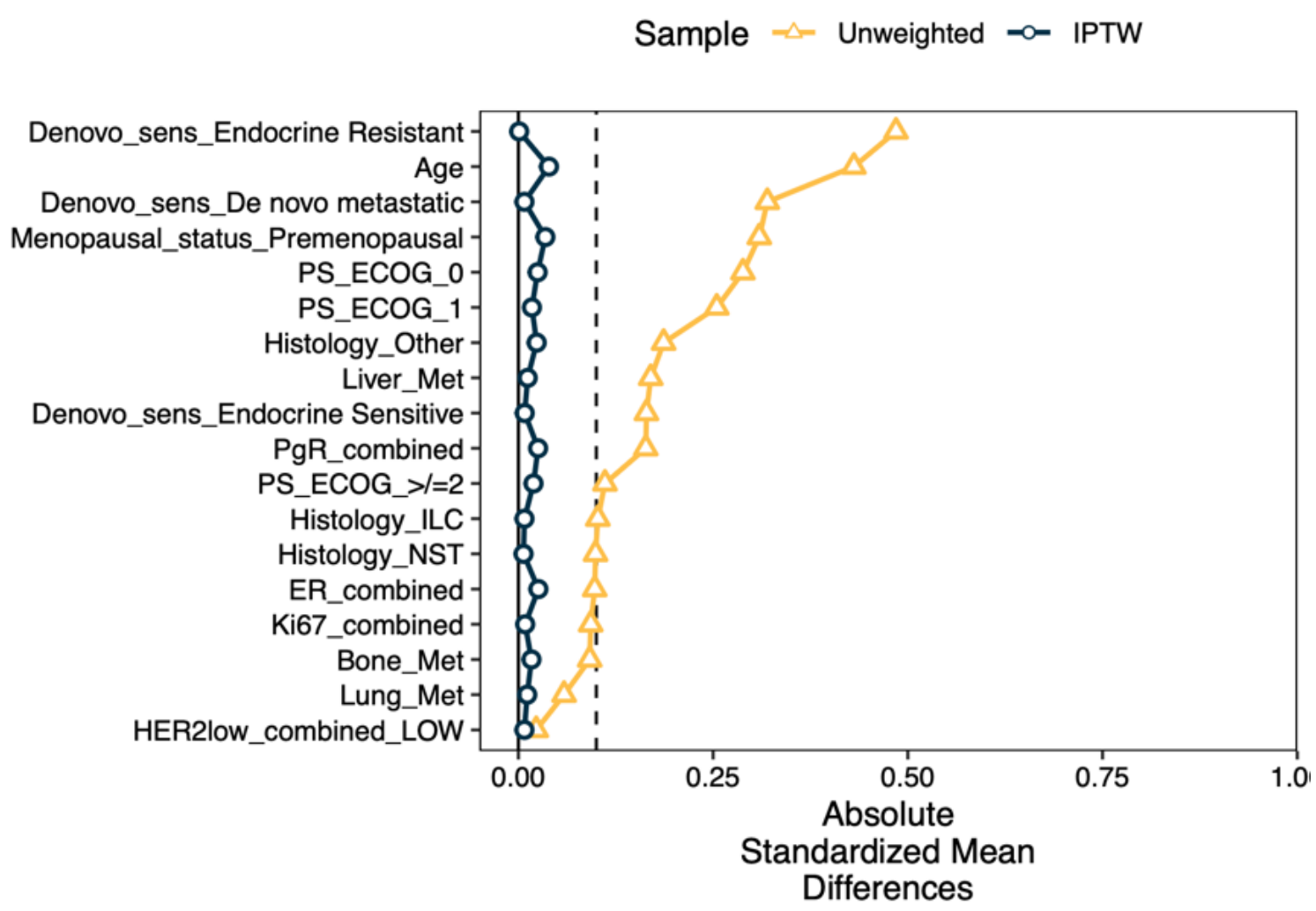

Supplementary Figure S4. Love Plots showing covariate balance plot before and after IPTW balance of treatment groups (palbociclib, ribociclib, abemaciclib).

The yellow and blue connected dots represent the covariates absolute standardized mean difference (d-value) before and after IPTW adjustments; vertical dotted line highlight the cutoffs for negligible (<0.1) d-values.

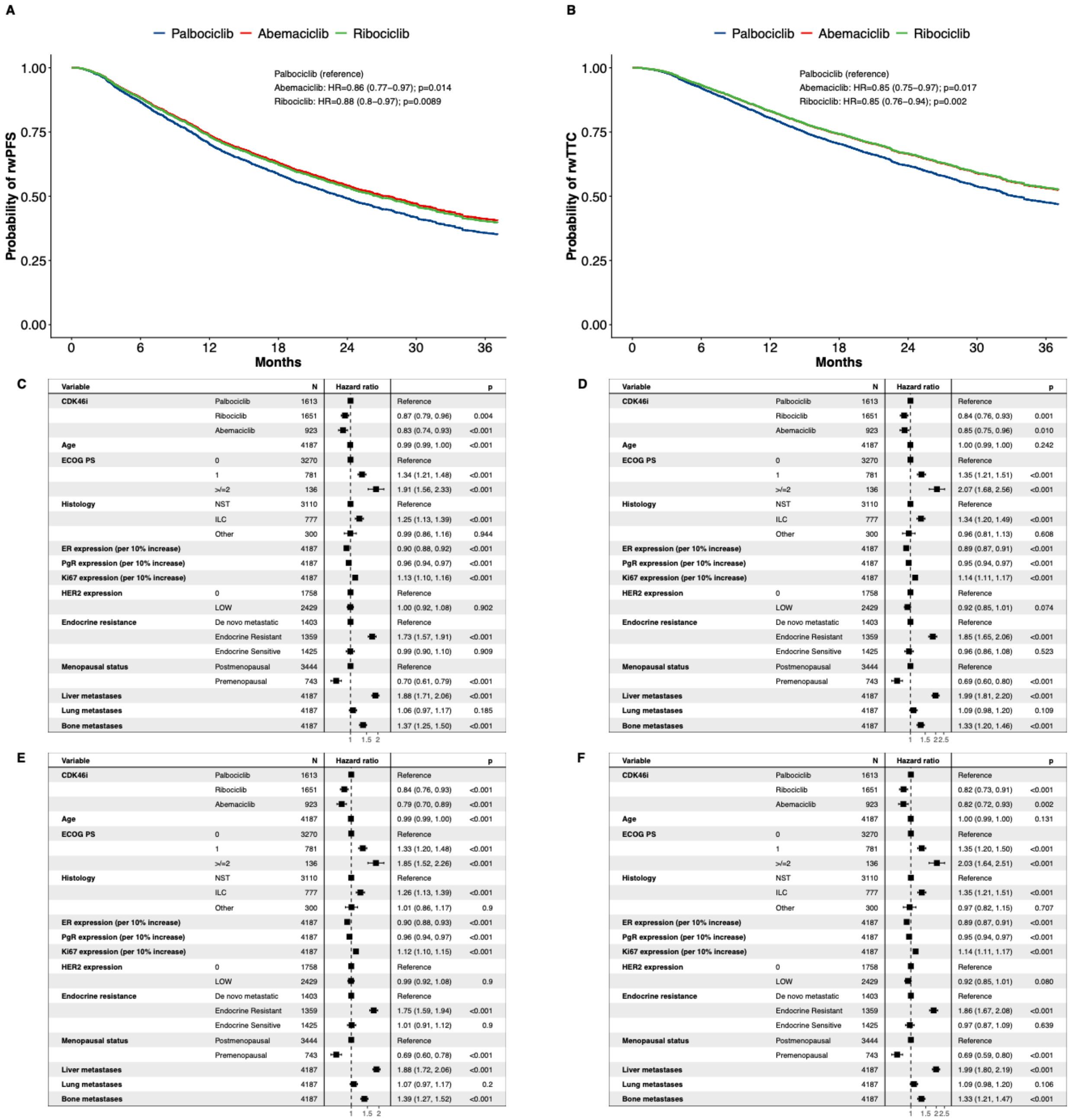

Supplementary Figure S5. rwPFS and rwTTC comparisons of palbociclib, ribociclib and abemaciclib.

Survival curves for rwPFS (A) and rwTTC (B) according to CDK4/6i treatment after IPTW balance. Forest plots illustrating the results of multivariable Cox regression models for rwPFS (C, E) and rwTTC (D, F). In E and F, sensitivity analyses stratifying Cox models by year of prescription as a continuous variable are reported. *CDK4/6i: cyclin-dependent kinase 4/6 inhibitors; ECOG PS: Eastern Cooperative Oncology Group Performance Status; ERα: Oestrogen Receptor; HER2: Human Epidermal growth factor Receptor 2; ILC: Invasive Lobular Carcinoma; NST: Non-Special Type; PgR: Progesterone Receptor, rwPFS: real-world progression-free survival; rwTTC real-world time to chemotherapy. P values refer to the Wald test from the Cox regression models.*

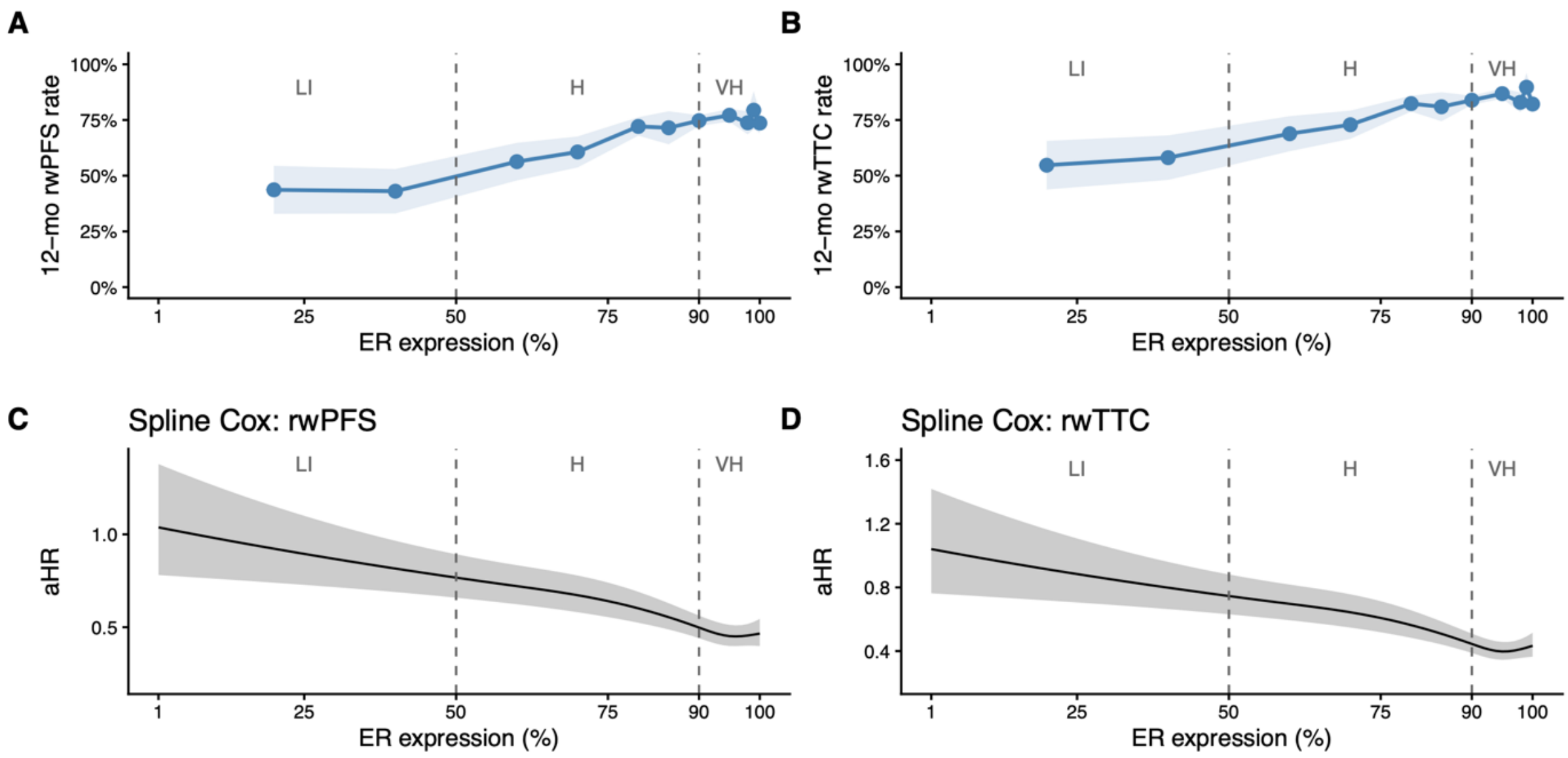

Supplementary Figure S6. Association between continuous ERα expression and rwPFS and rwTTC. (A-B) Sliding window analysis (STEPP framework) estimating 12-month rwPFS (A) and rwTTC (B) probability across overlapping subpopulations defined by continuous ER expression (window size ~2%, 50% overlap). Shaded areas represent 95% confidence intervals. (C-D) Restricted cubic spline analysis of continuous ER expression within a multivariable Cox model adjusted for CDK4/6i, age, ECOG PS, histology, PgR, Ki67, HER2-status, endocrine sensitivity, menopausal status, and presence of liver, lung and bone metastases. aHR, adjusted Hazard Ratio. Vertical dashed lines indicate predefined ER expression thresholds (50% and 90%). LI, low-intermediate (1-49%); H, high (50-89%); VH, very-high (90-100%).

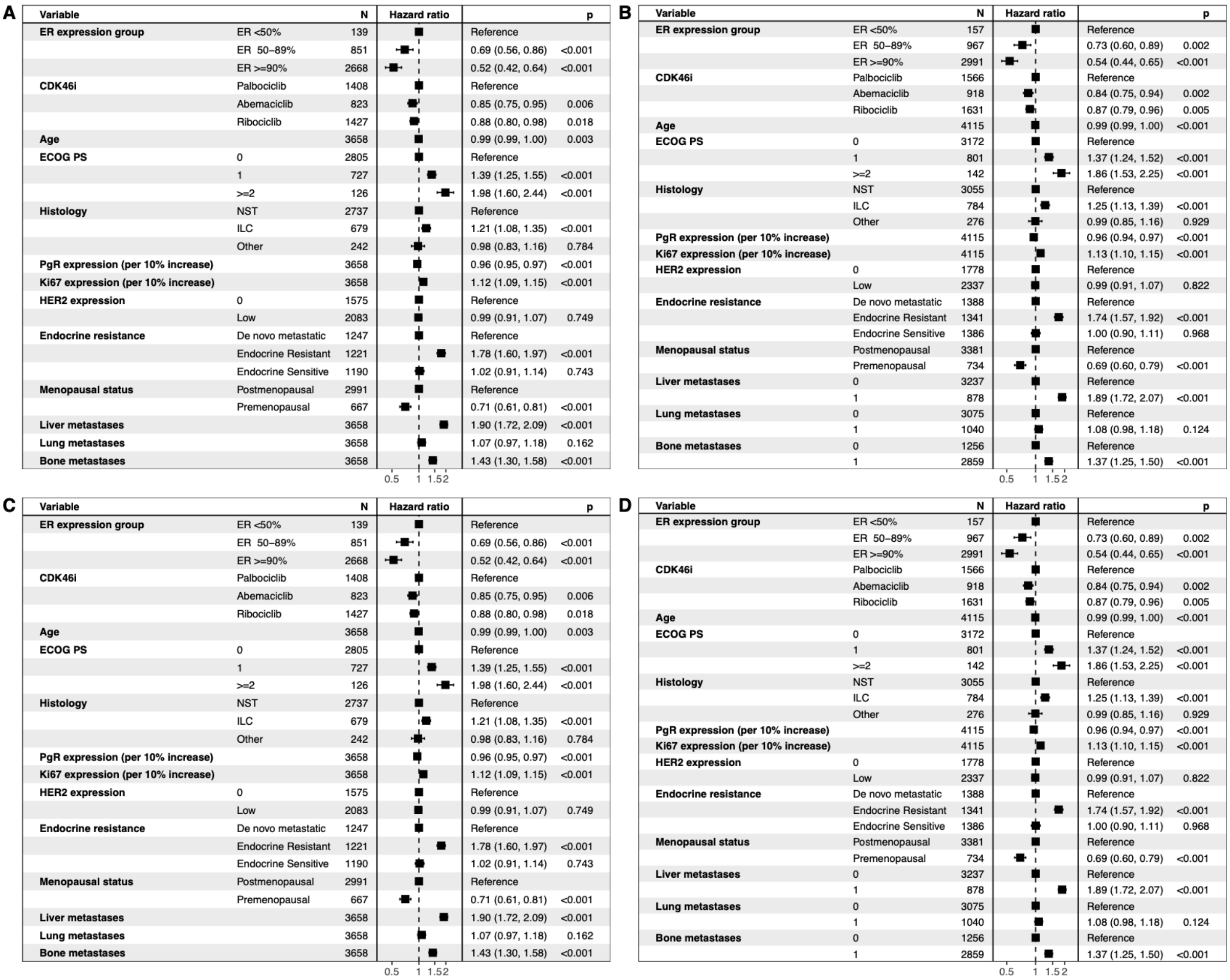

Supplementary Figure S7. Sensitivity analyses employing alternative missing-data handling. Forest plot illustrating the results of multivariable Cox regression model for (A,B) rwPFS and (C,D) rwTTC in the complete-case dataset (A,C) or after multiple imputation of missing covariates (B,D). *CDK4/6i: cyclin-dependent kinase 4/6 inhibitors; ECOG PS: Eastern Cooperative Oncology Group Performance Status; ERa: Estrogen Receptor alpha; HER2: Human Epidermal growth factor Receptor 2; ILC: Invasive Lobular Carcinoma; NST: Non-Special Type; PgR: Progesterone Receptor; rwPFS: real-world progression-free survival; rwTTC: real-world time to chemotherapy. P values refer to the Wald test from multivariable Cox regression models.*

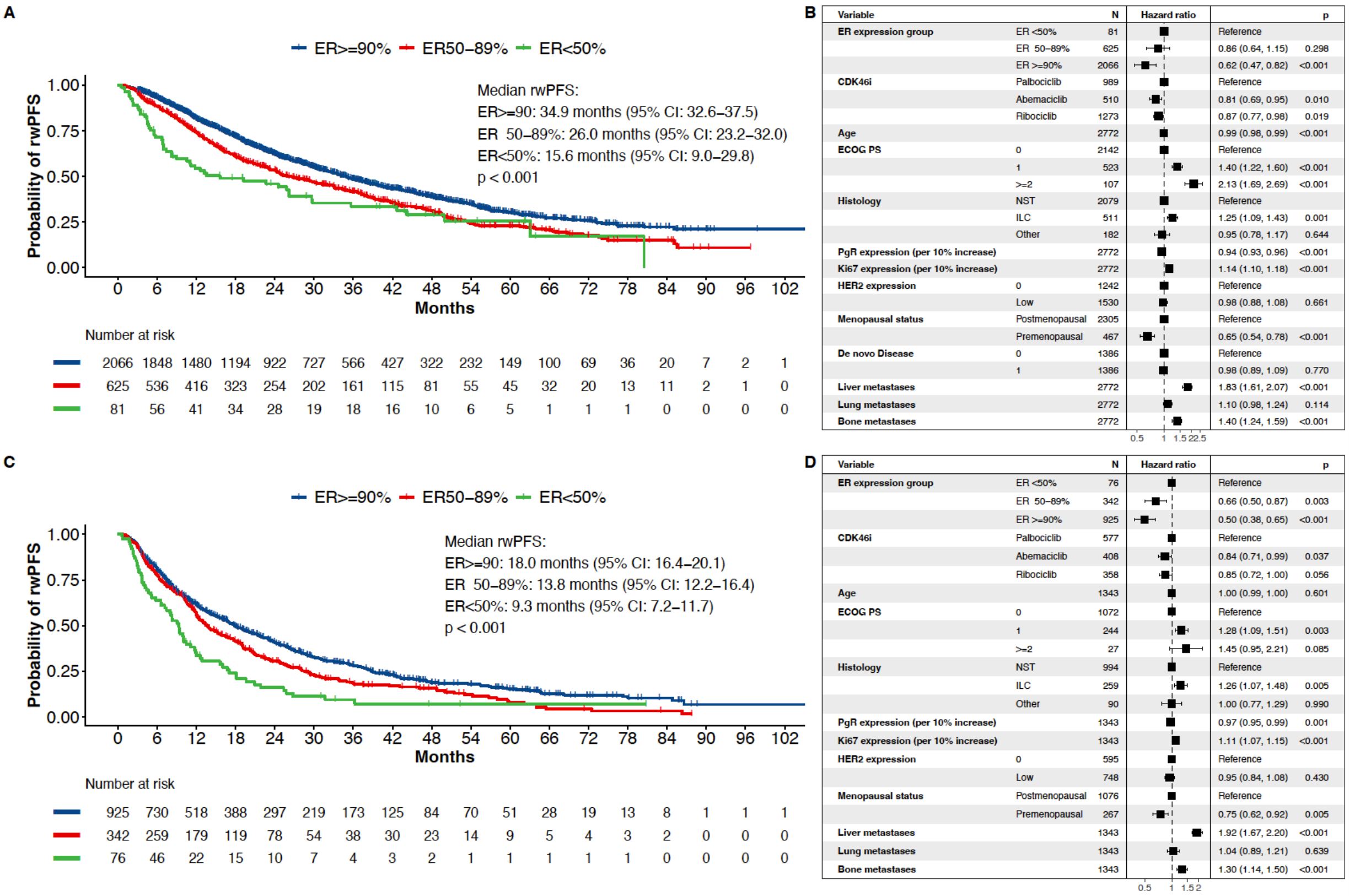

Supplementary Figure S8. Association between ERα expression and rwPFS according to tumour endocrine sensitivity or resistance.

Kaplan Meier curves for rwPFS according to ERα status (A, C) and Forest plot illustrating the results of multivariable Cox regression model (B,D) in the cohorts of patients with endocrine sensitive (A,B) or endocrine resistance disease (C,D). CDK4/6i: cyclin-dependent kinase 4/6 inhibitors; ECOG PS: Eastern Cooperative Oncology Group Performance Status; ERα: Oestrogen Receptor alpha; HER2: Human Epidermal growth factor Receptor 2; ILC: Invasive Lobular Carcinoma; NST: Non-Special Type; PgR: Progesterone Receptor; rwPFS: real-world progression-free survival. P values in panels A and C refer to the log-rank test; P values in panels B and D refer to the Wald test from the multivariable Cox regression models.

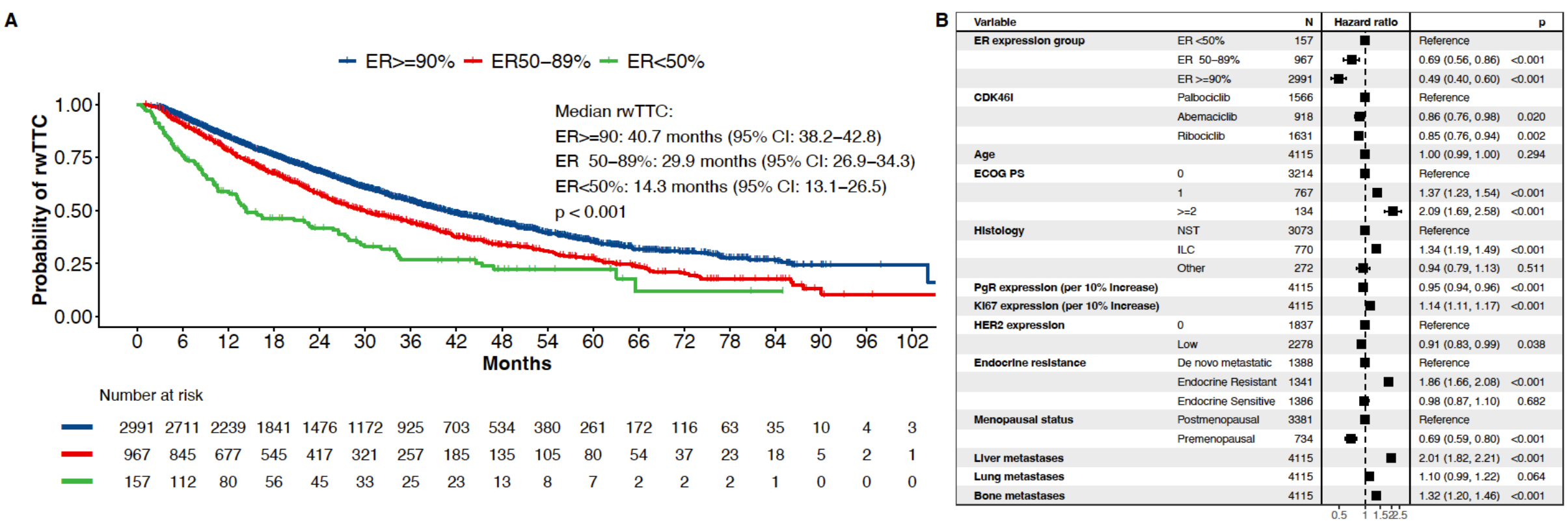

Supplementary Figure S9. Association between ERα expression and rwTTC.

Kaplan Meier curves for rwTTC according to ERα status (A) and Forest plot illustrating the results of multivariable Cox regression model (B) in the whole PALMARES-2 patient cohort. *CDK4/6i: cyclin-dependent kinase 4/6 inhibitors; ECOG PS: Eastern Cooperative Oncology Group Performance Status; ERα: Oestrogen Receptor alpha; HER2: Human Epidermal growth factor Receptor 2; ILC: Invasive Lobular Carcinoma; NST: Non-Special Type; PgR: Progesterone Receptor; rwTTC: real-world time to chemotherapy. P values in panel A refer to the log-rank test; P values in panel B refer to the Wald test from the multivariable Cox regression model.*

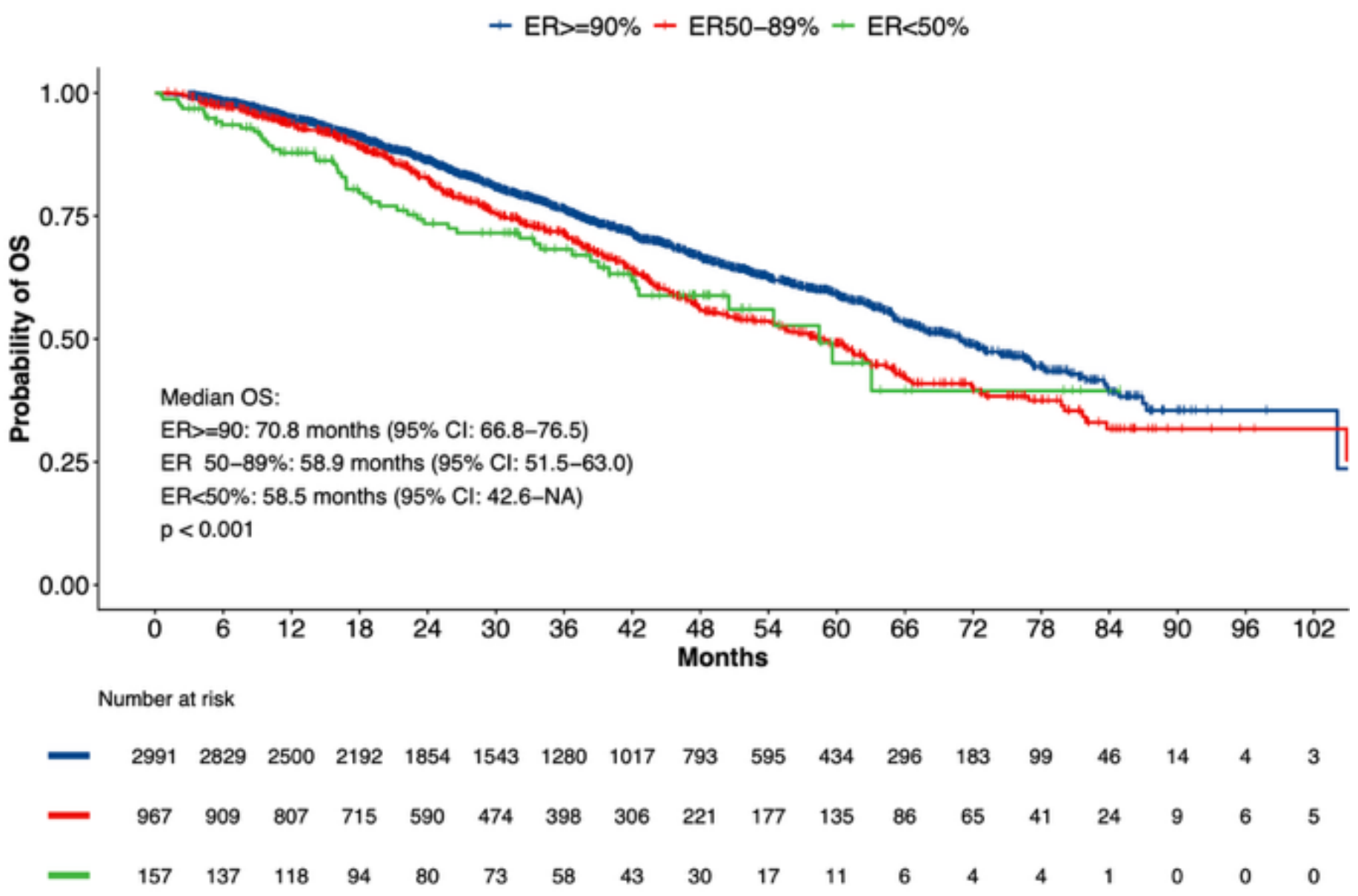

Supplementary Figure S10. Overall survival according to ERα expression category. Kaplan-Meier curves for overall survival (OS) stratified by ERα expression: ER-VH (≥90%, blue), ER-H (50–89%; red), and ER-LI (<50% green). *ERa: Estrogen Receptor; OS: Overall survival.*

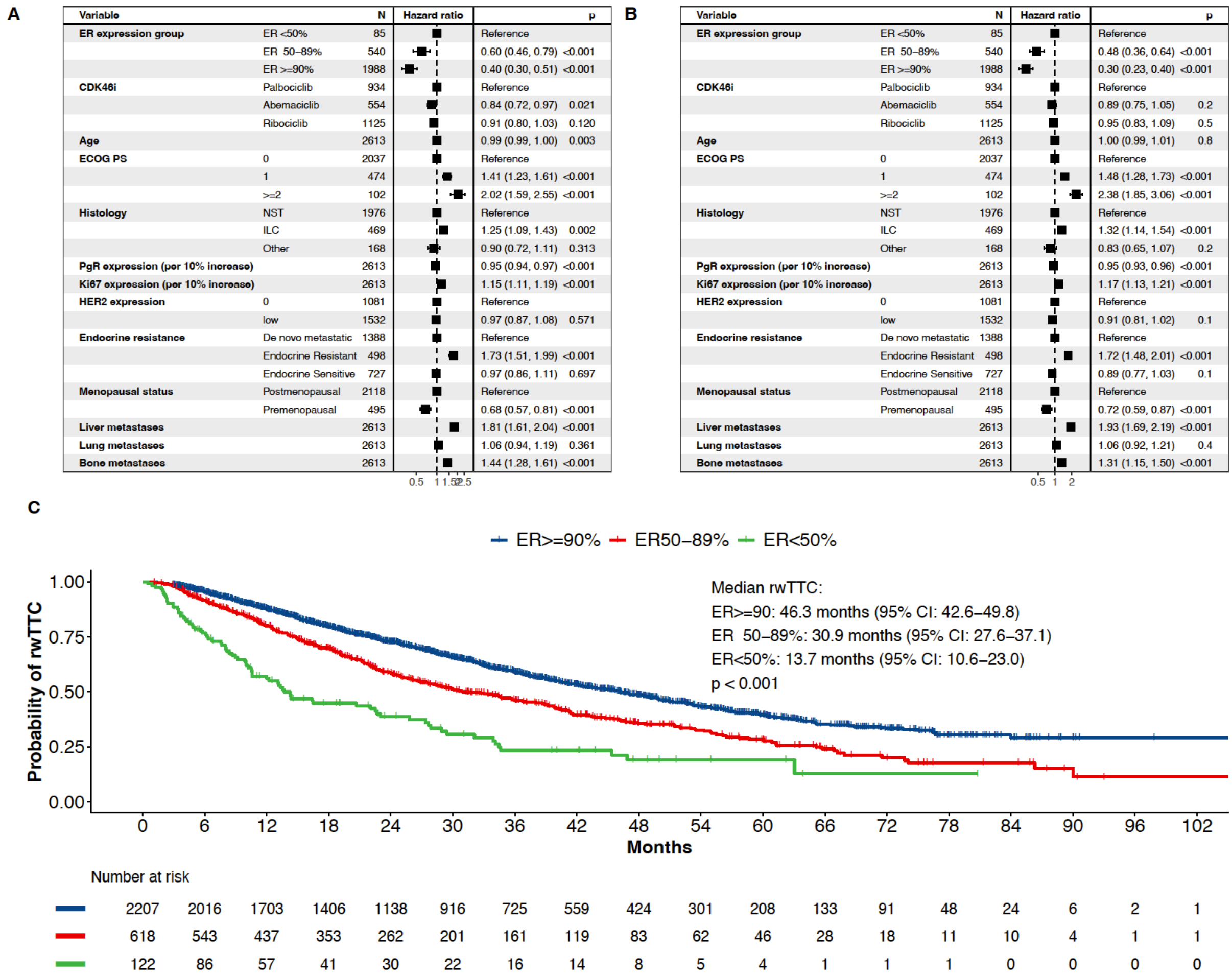

Supplementary Figure S11. Association between ERα expression at advanced disease diagnosis and rwTTC (A, B) Forest plot illustrating the results of multivariable Cox regression model for (A) rwPFS or (B) rwTTC and (C) Kaplan Meier curves for rwTTC according to ERα status in the cohort of patients with ERα expression status assessed in a tumour specimen collected at the time of advanced disease diagnosis. *CDK4/6i: cyclin-dependent kinase 4/6 inhibitors; ECOG PS: Eastern Cooperative Oncology Group Performance Status; ERα: Oestrogen Receptor alpha; HER2: Human Epidermal growth factor Receptor 2; ILC: Invasive Lobular Carcinoma; NST: Non-Special Type; PgR: Progesterone Receptor; rwTTC: real-world time to chemotherapy. P values in panel A and B refer to the Wald test from the multivariable Cox regression models; P value in panel C refer to the log-rank test.*

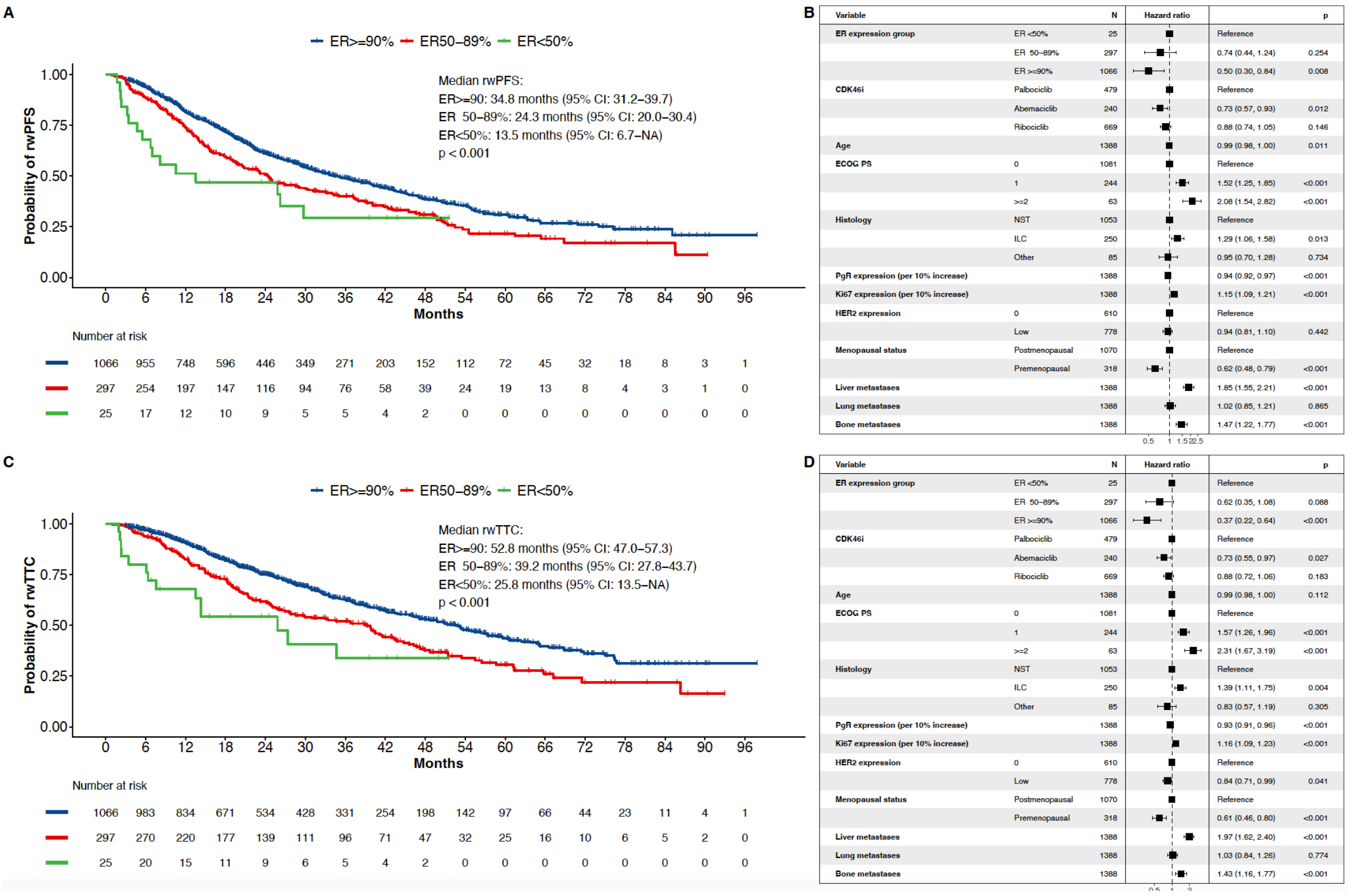

Supplementary Figure S12. Prognostic value of ERα expression in patients with de novo metastatic disease. Kaplan Meier curves for rwPFS (A) and rwTTC (C) according to ERα status and Forest plot illustrating the results of multivariable Cox regression models for rwPFS (B) and rwTTC (D) in patients diagnosed with *de novo* metastatic disease. *CDK4/6i: cyclin-dependent kinase 4/6 inhibitors; ECOG PS: Eastern Cooperative Oncology Group Performance Status; ERα: Oestrogen Receptor alpha; HER2: Human Epidermal growth factor Receptor 2; ILC: Invasive Lobular Carcinoma; NST: Non-Special Type; PgR: Progesterone Receptor; rwPFS: real-world progression-free survival. rwTTC: real-world time to chemotherapy. P values in panel A refer to the log-rank test; P values in panel B refer to the Wald test from the multivariable Cox regression model.*

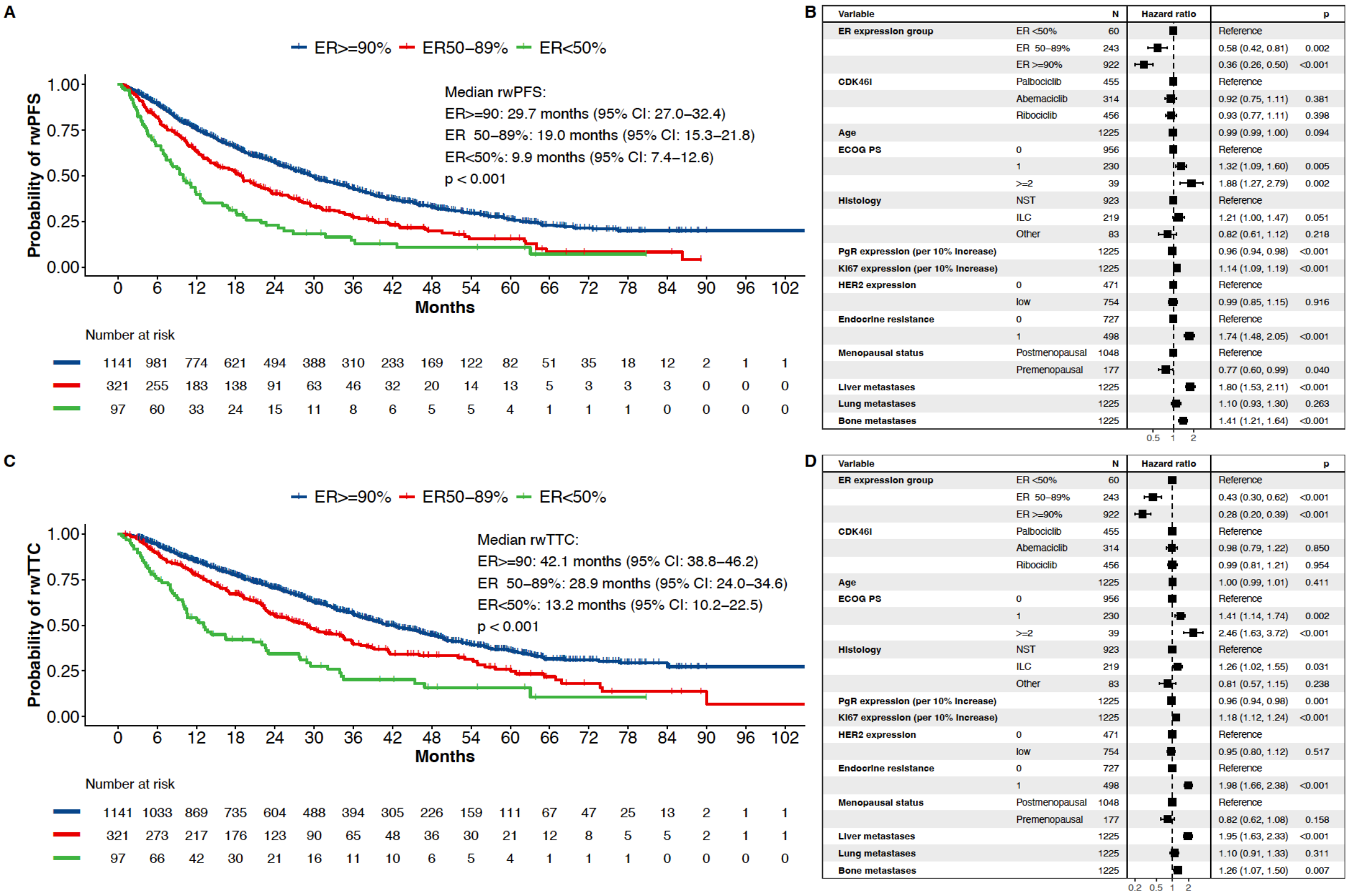

Supplementary Figure S13. Prognostic value of ERα expression in relapsed tumour specimens from patients with metachronous metastases. Kaplan Meier curves for rwPFS (A) and rwTTC (C) according to ERα status and Forest plot illustrating the results of multivariable Cox regression models for rwPFS (B) and rwTTC (D) in the cohort of patients with metachronous aBC and ERα status assessed in a tumour specimen collected at the time of advanced disease diagnosis. *CDK4/6i: cyclin-dependent kinase 4/6 inhibitors; ECOG PS: Eastern Cooperative Oncology Group Performance Status; ERα: Oestrogen Receptor alpha; HER2: Human Epidermal growth factor Receptor 2; ILC: Invasive Lobular Carcinoma; NST: Non-Special Type; PgR: Progesterone Receptor;* rwPFS*: real-world progression-free survival. P values in panels A and C refer to the log-rank test; P values in panels B and D refer to the Wald test from the multivariable Cox regression model.*

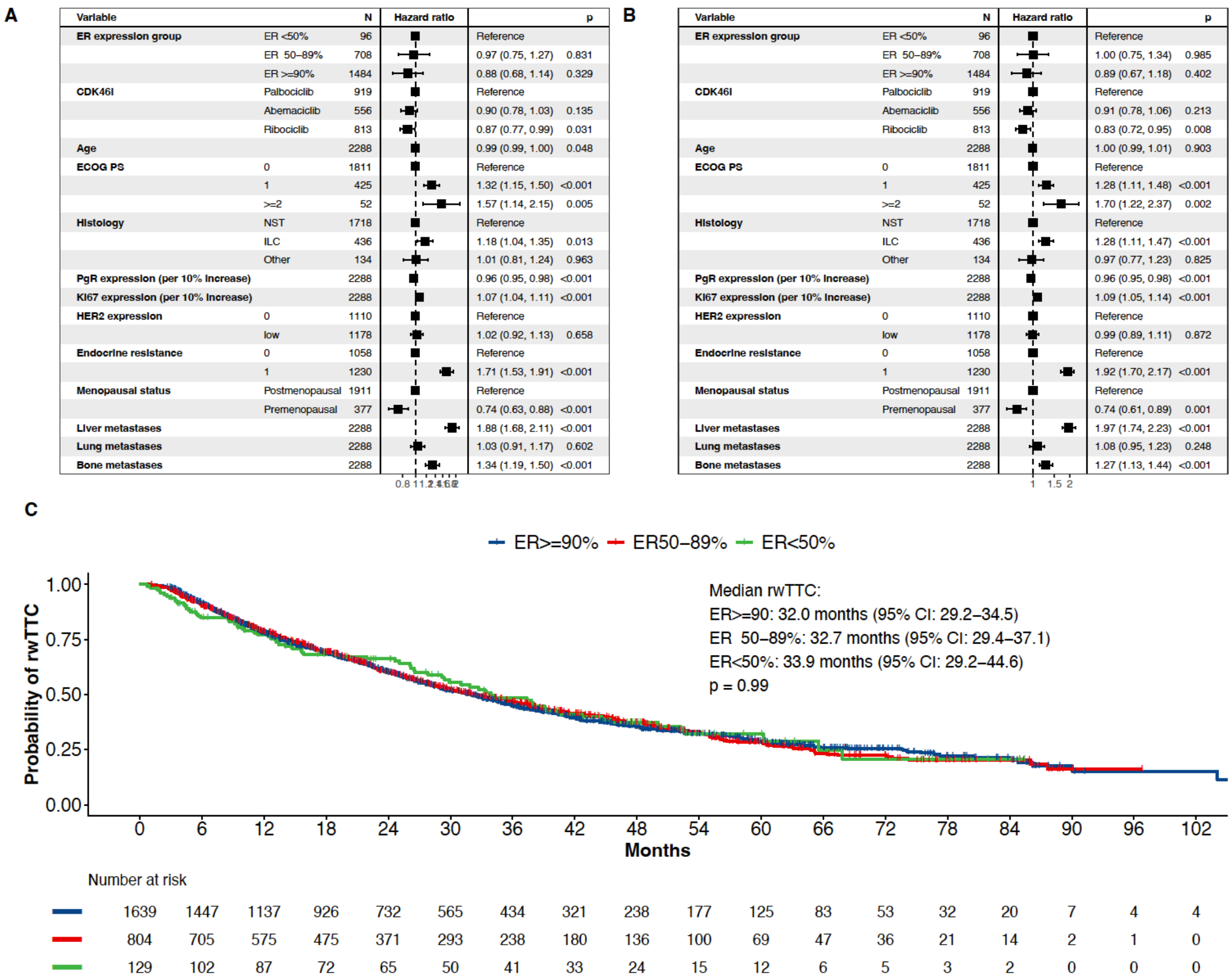

Supplementary Figure S14. Prognostic value of ERα expression in primary tumour specimens from patients with metachronous metastases. (A,B) Forest plot illustrating the results of multivariable Cox regression models for rwPFS (A) and rwTTC (B), and Kaplan Meier curves for rwTTC (C) according to ERα status in the cohort of patients with metachronous aBC and ERα expression status assessed in the primary tumour specimen. *CDK4/6i: cyclin-dependent kinase 4/6 inhibitors; ECOG PS: Eastern Cooperative Oncology Group Performance Status; ERα: Oestrogen Receptor alpha; HER2: Human Epidermal growth factor Receptor 2; ILC: Invasive Lobular Carcinoma; NST: Non-Special Type; PgR: Progesterone Receptor;* rwPFS*: real-world progression-free survival. P values in panels A and B refer to the Wald test from the multivariable Cox regression models.. P value in panel C refer to the log-rank test.*

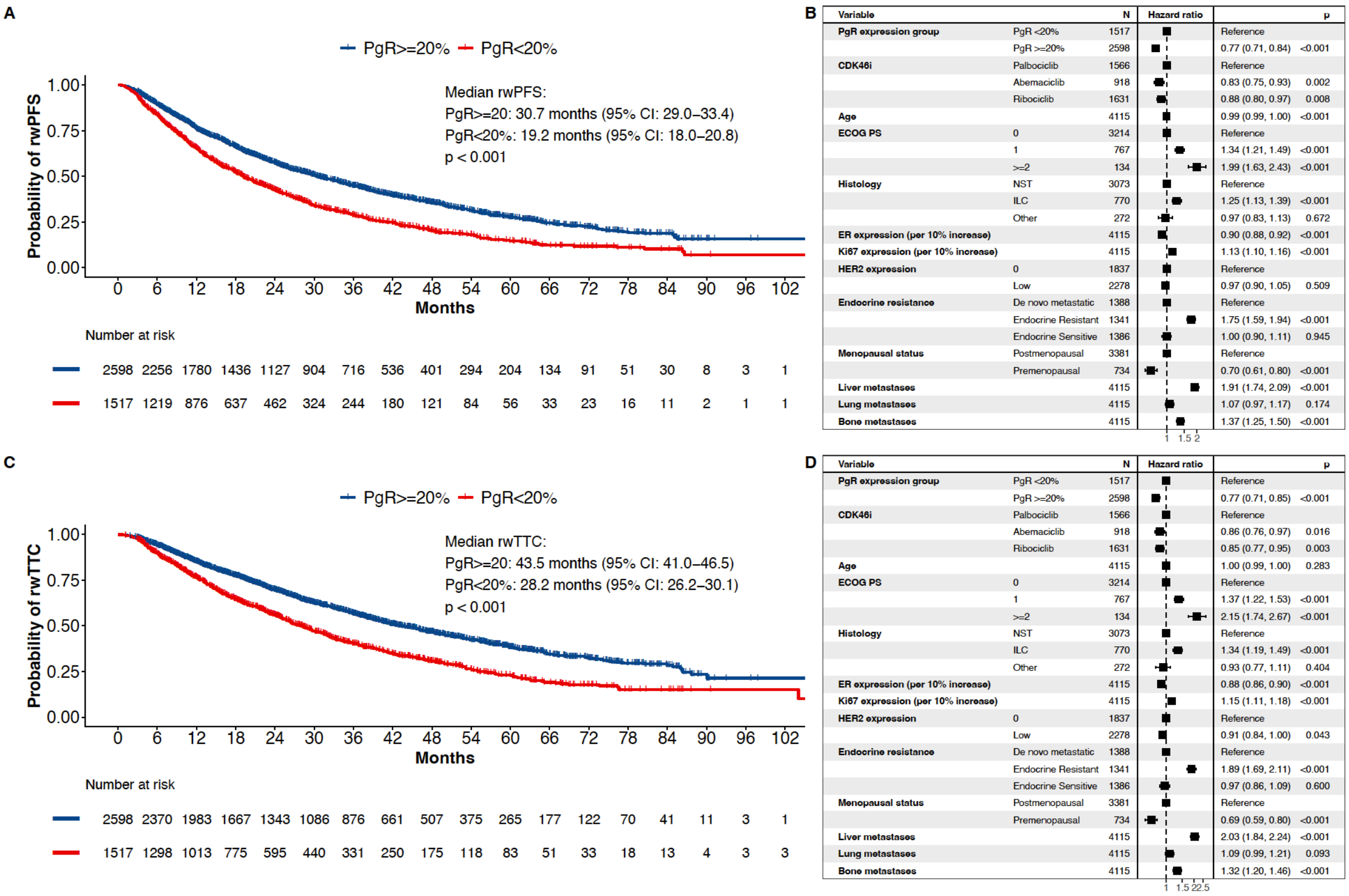

Supplementary Figure S15. Real-world Progression Free Survival according to PgR expression. Kaplan Meier curves for rwPFS (A) and rwTTC (C) according to PgR status (high: ≥20%vs. low: <20%) and Forest plot illustrating the results of multivariable Cox regression model for rwPFS (B) and rwTTC (D) in the whole patient cohort. rwPFS*: real world progression-free survival. rwTTC: real-world time to chemotherapy. CDK4/6i: cyclin-dependent kinase 4/6 inhibitors; ECOG PS: Eastern Cooperative Oncology Group Performance Status; ERα: Oestrogen Receptor alpha; HER2: Human Epidermal growth factor Receptor 2; ILC: Invasive Lobular Carcinoma; NST: Non-Special Type; PgR: Progesterone Receptor. P values in panels A and C refer to the log-rank test; P values in panels B and D refer to the Wald test from the multivariable Cox regression model.*

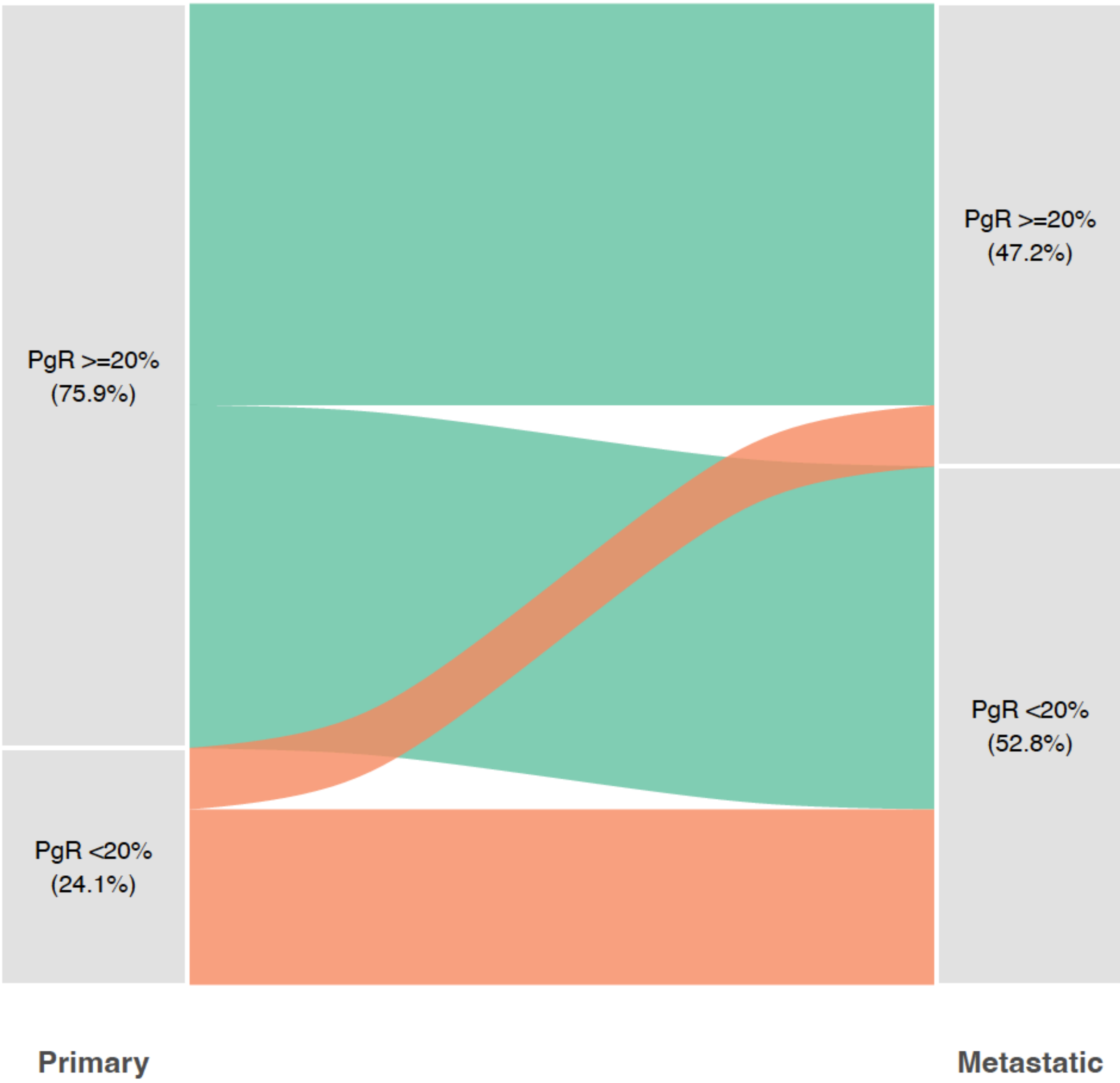

Supplementary Figure S16. Changes in PgR expression in primary vs. relapses tumours
Alluvial plot illustrating PgR status assessed in the primary tumour sample (left) and in matched metastastic samples collected before first CDK4/6i plus ET treatment initiation (right) in the subgroup of 1394 patients in which both assessments were available. *PgR: Progesterone Receptor.*

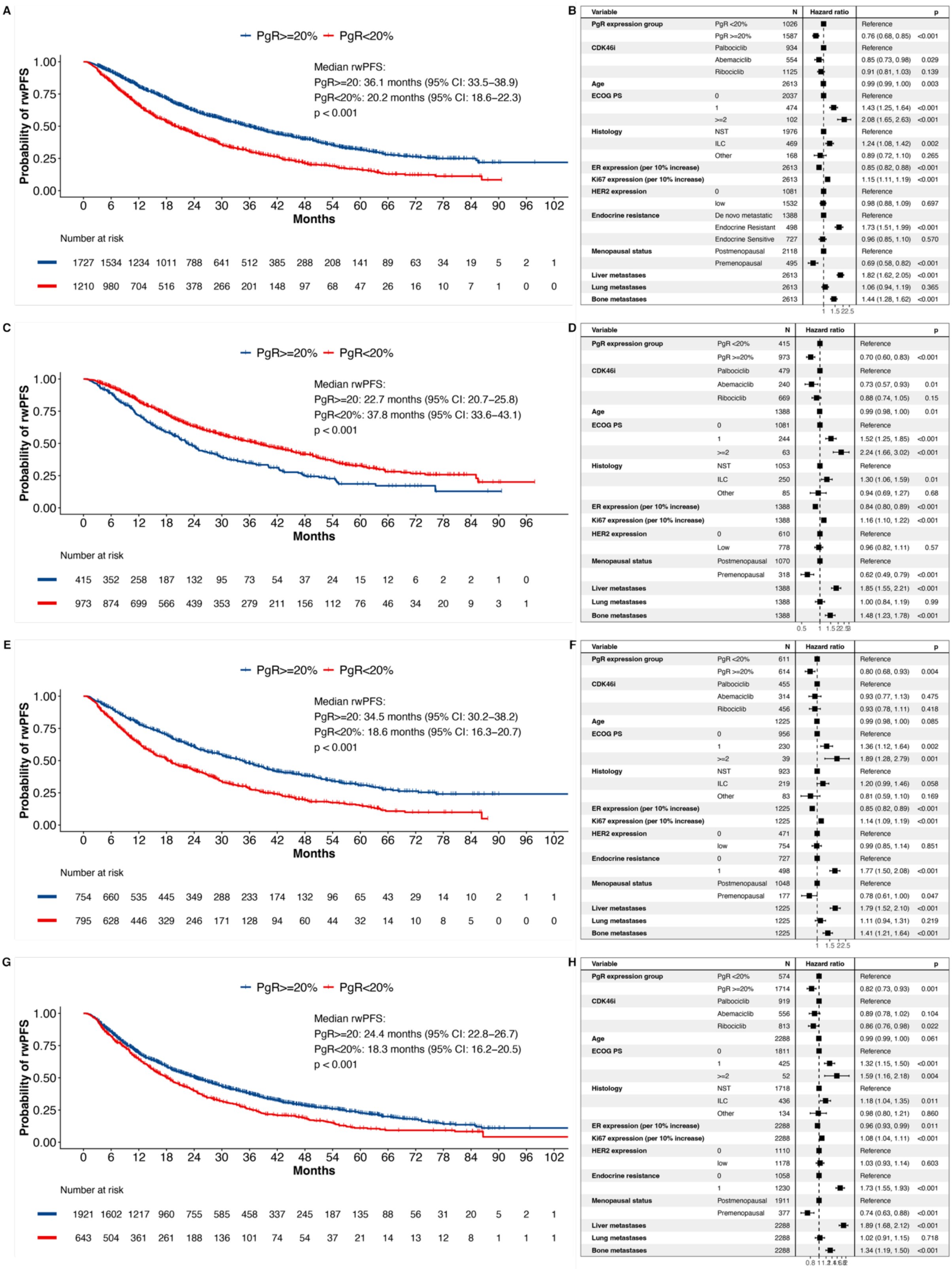

Supplementary Figure S17. Association between PgR expression and real-world Progression Free Survival according to the timing of assessment.

Kaplan Meier curves (A, C, E, G) for rwPFS according to PgR status (high: ≥20% vs. low: <20%) and Forest plots (B, D, F, H) illustrating the results of multivariable Cox regression models for rwPFS: (A, B) in the cohort of patients with PgR expression status assessed in tumour specimens collected at advanced disease diagnosis, (C,D) in the cohort of patients

diagnosed with de novo metastatic disease, (E, F) in the cohort of patients with metachronous advanced breast cancer and ER expression status assessed at advanced disease diagnosis and (G, H) in the cohort of patients with metachronous advanced breast cancer and ERα expression status assessed in the primary tumour. rwPFS*: real-world progression-free survival.; CDK4/6i: cyclin-dependent kinase 4/6 inhibitors; ECOG PS: Eastern Cooperative Oncology Group Performance Status; ERα: Oestrogen Receptor; HER2: Human Epidermal growth factor Receptor 2; ILC: Invasive Lobular Carcinoma; NST: Non-Special Type; PgR: Progesterone Receptor. P values in panels A, C, E, D refer to the log-rank test; P values in panels B,D, F, H refer to the Wald test from the multivariable Cox regression model.*

A

B

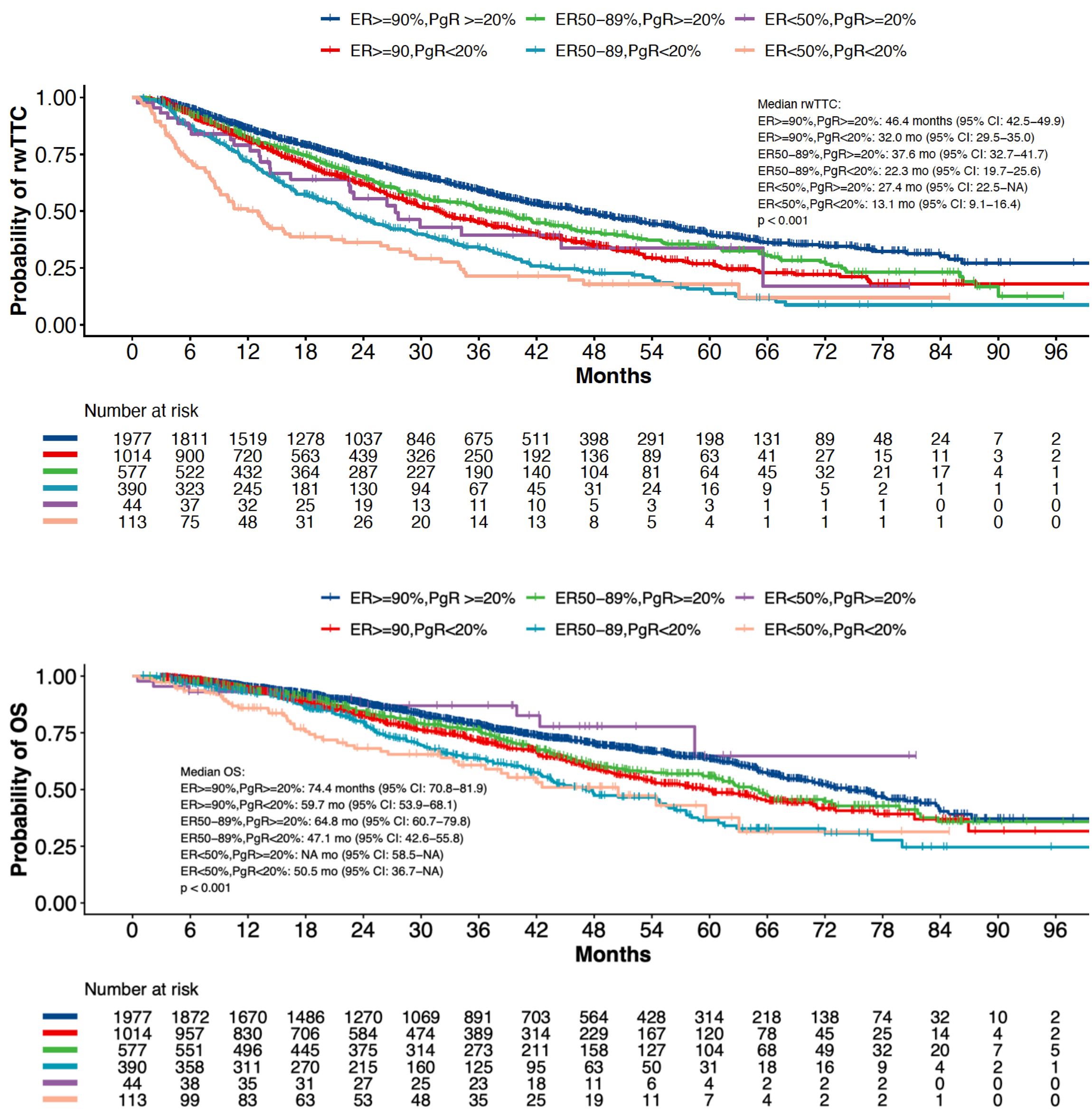

Supplementary Figure S18. Real-world Time To Chemotherapy and Overall Survival according to ERα and PgR expression. Kaplan Meier curves for rwTTC an OS according to ERα status and PgR status in the whole patient cohort. rwTTC*: real-world time to*

chemotherapy; OS: Overall Survival; ERα: Oestrogen Receptor; PgR: Progesterone Receptor. P value refers to the log rank test

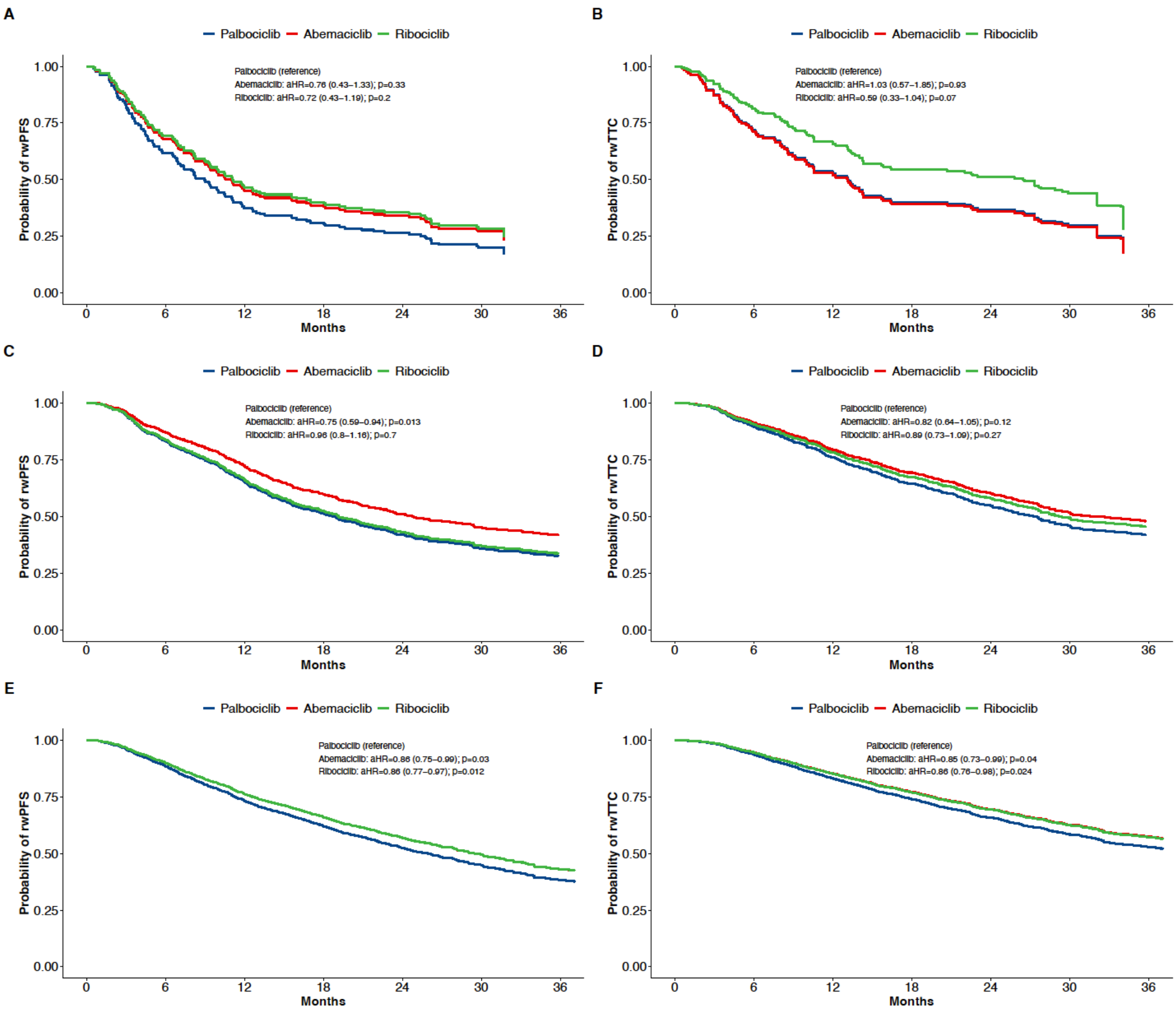

Supplementary Figure S19. Adjusted rwPFS and rwTTC comparisons between palbociclib, ribociclib and abemaciclib in each ERa category. Survival curves for rwPFS (A,C,E) and rwTTC (B,D,F) in ER-LI (A,B), ER-H (C-D) and ER-VH (E,F) cohorts according to CDK4/6i treatment after covariate adjusting (multivariable cox regression models). aHR *adjusted Hazard Ratio*; rwPFS*: real-world progression-free survival; CDK4/6i: cyclin-dependent kinase 4/6 inhibitors;* rwTTC*: real-world time to chemotherapy. ERα: Oestrogen Receptor. P values refer to the Wald test from the multivariable Cox regression model.*

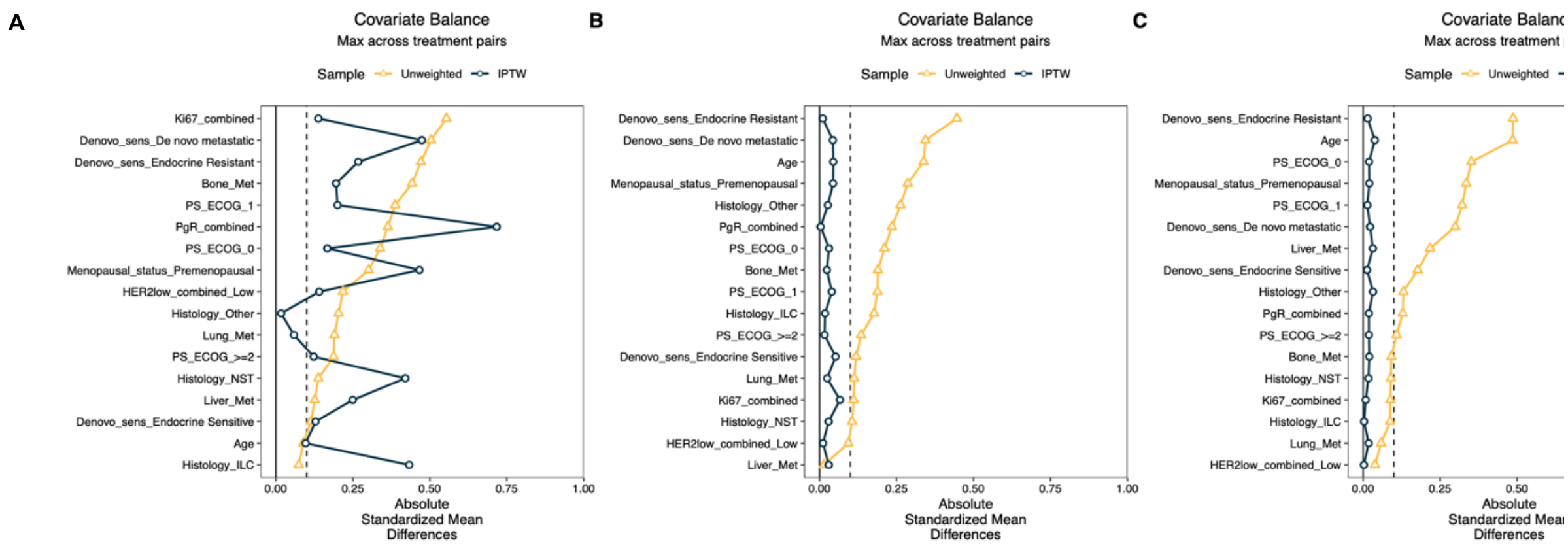

Supplementary Figure S20. Love Plots showing covariate balance plot before and after IPTW balance of treatment groups (palbociclib, ribociclib, abemaciclib) in patients with (A) ER-LI, (B) ER-H, (C) ER-VH tumors. The yellow and blue connected dots represent the covariates absolute standardized mean difference (d-value) before and after IPTW adjustments; vertical dotted line highlight the cutoffs for negligible (<0.1) d-values.

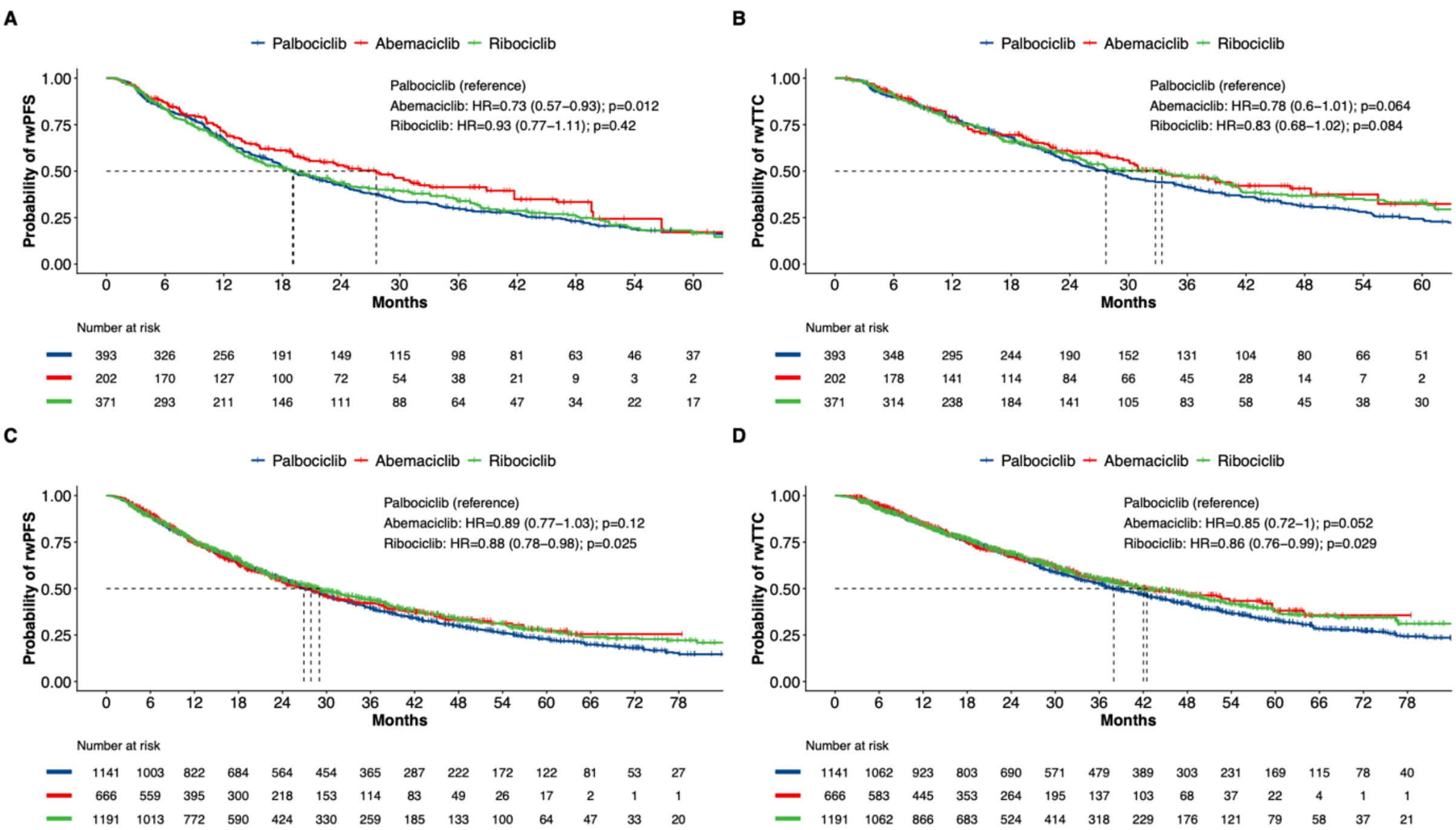

Supplementary Figure S21. rwPFS and rwTTC comparisons between palbociclib, ribociclib and abemaciclib after IPTW in ER-H and ER-VH subgroups. Kaplan Meier curves for rwPFS (A,C) and rwTTC (B,D) ER-H (A-B) and ER-VH (C,D) according to CDK4/6i treatment after IPTW balance. *rwPFS: real-world progression-free survival; CDK4/6i: cyclin-dependent kinase 4/6 inhibitors; rwTTC: real-world time to chemotherapy. ER: Estrogen Receptor. IPTW: Inverse-Probability of Treatment Weighting*

SUPPLEMENTARY TABLES

Supplementary Table S1. Baseline features collected within the PALMARES-2 study. Each feature is listed with corresponding definitions.

**Feature**	**Description**
Age	Patient age at first-line treatment start
Sex	Biological sex
Menopausal_status	Patient menopausal status, as defined by the use of Luteinizing Hormone-Releasing Hormone (LHRH) analogue as first-line endocrine therapy
HRDNot.mutated	Pathogenic germline mutation at *BRCA1*, *BRCA2* or *PALB2* gene
HRDMutated	No pathogenic germline mutation at *BRCA1*, *BRCA2* or *PALB2* gene
PS_ECOG	Eastern Cooperative Oncology Group performance status at first-line treatment baseline
HistologyNST	Non-Special Type carcinoma as histological subtype*
HistologyCLI	Classic Lobular Invasive carcinoma as histological subtype*
ER_combined	Oestrogen receptor expression status*
PgR_combined	Progesterone receptor expression status*
Ki67_combined	Ki-67 proliferation index*
Grading_combined	Tumour grading (combined scale)*
HER2_combined	HER2 expression status*
Luminal	Luminal-like molecular subtype: luminal-A, defined as Ki67 tumour level less than 20% AND presence of progesterone receptor tumour expression (≥1%); luminal-B, defined as Ki67 level more than 20% OR absence of progesterone receptor tumour expression (<1%)*
Endocrine_Resistance	Detection of inoperable/metastatic tumour relapse during the course of adjuvant ET, or within 12 months from the completion of adjuvant ET (Cardoso et al. Ann Onc; 2020;31(12):1623-1649)
Primary_Resistance	Detection of inoperable/metastatic tumour relapse within 24 months after the initiation of adjuvant ET (Cardoso et al. Ann Onc; 2020;31(12):1623-1649)
Secondary_Resistance	Detection of inoperable/metastatic tumour relapse between 24 months after the initiation and within 12 months from the end of adjuvant ET (Cardoso et al. Ann Onc; 2020;31(12):1623-1649)

DFI	Disease-free interval, defined as the time in months between primary tumour surgery and inoperable/metastatic relapse
ChT_neoadj	Prior exposure to neoadjuvant chemotherapy for early-stage breast cancer
ChT_adj	Prior exposure to adjuvant chemotherapy for early-stage breast cancer
ET_adj	Prior exposure to adjuvant endocrine therapy for early-stage breast cancer
ET_adj_typeAI	Aromatase inhibitor as the last adjuvant endocrine therapy for early-stage breast cancer
ET_adj_typeTAM	Tamoxifen as the last adjuvant endocrine therapy for early-stage breast cancer
Adj_ET_duration	Duration in months of the last prior adjuvant endocrine therapy for early-stage breast cancer
Previous_Antra	Prior exposure to anthracycline therapy for early-stage breast cancer
Previous_Tax	Prior exposure to taxane therapy for early-stage breast cancer
aBC_de_novo	De novo presentation of metastatic breast cancer
Liver_Met	Presence of liver metastases
Lung_Met	Presence of lung metastases
Bone_Met	Presence of bone metastases
SNC_Met	Presence of central nervous system metastases
Soft_Tissue_Met	Presence of soft tissue metastases
Node_Met	Presence of lymph node metastases
Pleura_Peritoneum	Presence of pleural or peritoneal metastases
Other_Met	Presence of other metastatic site(s) other than liver, lung, bone, central nervous system, soft tissue, lymph node, pleura or peritoneum
Num_Met_Sites	Number of organs with the presence of at least one metastasis
Bone_Only	Bone-only metastatic disease
Visceral_Met	Presence of at least one metastasic site among the following visceral organs: liver, lung, pleura, peritoneum, central nervous system
CDK46i_DrugPalbociclib	Use of palbociclib as first-line CDK4/6i
CDK46i_DrugRibociclib	Use of ribociclib as first-line CDK4/6i
ET_CDK46i_concomitant_start	Concomitant initiation of endocrine therapy and CDK4/6i as first-line treatment
ET_1L_type	Type of endocrine therapy used as first-line treatment (aromatase inhibitor vs. tamoxifen)
Time_ET_CDK46i	Time in days occurred between endocrine therapy and CDK4/6i start initiation as first-line treatment

HCV	Known history of Hepatitis C infection
HBV	Known history of Hepatitis B infection
Comorbidity_Number	Total number of comorbidities
Comorbidity_Cardiovascular	Known history of cardiovascular comorbidity
Comorbidity_Hematological	Known history of hematologic comorbidity
Comorbidity_Endoreumatoimmunological	Known history of endocrine, rheumatologic or immunologic comorbidity
Comorbidity_Hepatological	Known history of hepatological comorbidity
Comorbidity_Gastroenterological	Known history of gastrointestinal comorbidity
Comorbidity_Infectious	Known history of infectious disease comorbidity
Comorbidity_Metabolic	Known history of metabolic comorbidity
Comorbidity_Nefrourogynecological	Known history of renal or urological comorbidity
Comorbidity_Neuropsychiatric	Known history of neurologic or psychiatric comorbidity
Comorbidity_Oncological	Known history of other malignancy
Comorbidity_Respiratory	Known history of respiratory comorbidity
Comorbidity_previous_surgery	Prior major surgery other than breast surgery
Comorbidity_Hypertension	Known history of hypertension
Comorbidity_Dyslipidemia	Known history of dyslipidemia
Comorbidity_Hypothyroidism	Known history of hypothyroidism
Comorbidity_DM2	Known history of type 2 diabetes mellitus
Comorbidity_Osteoporosis	Known history of osteoporosis
Comorbidity_Depression	Known history of depression
Comorbidity_GERD	Known history of gastroesophageal reflux disease
Comorbidity_Atrial_Fibrillation	Known history of atrial fibrillation
Comorbidity_Anxiety_Depression_Syndrome	Known history of anxiety-depressive disorder
Comorbidity_Hyperthyroidism	Known history of hyperthyroidism
Comorbidity_Gastritis	Known history of gastritis
Comorbidity_Arrythmia_noAF	Known history of cardiac arrhythmia other than atrial fibrillation
Comorbidity_Appendicectomy	Known history of appendectomy
Comorbidity_Asthma	Known history of asthma
Comorbidity_COPD	Known history of chronic obstructive pulmonary disease
Comorbidity_Arthrosis	Known history of osteoarthrosis
Comorbidity_Cholecystectomy	Known history of cholecystectomy
Comorbidity_Anemia	Known history of anemia
Comorbidity_Anxiety	Known history of anxiety
Comorbidity_CKD	Known history of chronic kidney disease
ConcomitantMed_Anticoagulant	Use of anticoagulant drug(s)**
ConcomitantMed_Antibiotics	Use of antibiotic medications**
ConcomitantMed_Neurolectics	Use of neuroleptic drugs**
ConcomitantMed_BZN	Use of benzodiazepines**
ConcomitantMed_FANS	Use of NSAIDs**
ConcomitantMed_PPI	Use of proton pump inhibitor**
ConcomitantMed_Opioid	Use of opioid analgesics**

ConcomitantMed_Hormon_Vit	Use of hormonal or vitamin supplement(s)**
ConcomitantMed_BoneHealth	Use of bisphosphonates or bone health drug(s)**
ConcomitantMed_Steroids	Use of corticosteroids**
ConcomitantMed_Number	Number of concomitant medications
ConcomitantMed_VitD	Use of vitamin D supplementation**
ConcomitantMed_levothyroxine	Levothyroxine therapy**
ConcomitantMed_ASA	Aspirin therapy**
ConcomitantMed_Metformin	Metformin therapy**
ConcomitantMed_Insulin	Insulin therapy**
ConcomitantMed_Bisoprolol	Bisoprolol therapy**
ConcomitantMed_Ramipril	Ramipril therapy**
ConcomitantMed_Amlodipine	Amlodipine therapy**
ConcomitantMed_Nebivolol	Nebivolol therapy**
ConcomitantMed_Paracetamol	Paracetamol therapy**
ConcomitantMed_Furosemide	Furosemide therapy**
ConcomitantMed_Olmesartan	Olmesartan therapy**
ConcomitantMed_Atenolol	Atenolol therapy**
ConcomitantMed_Enoxaparin	Enoxaparin treatment**
ConcomitantMed_Allopurinol	Allopurinol therapy**
Center_sizeHigh_Burden	High-volume enrolling centre, defined as any participating institution falling within the highest tertile of patient accrual in the PALMARES-2 study
Center_sizeLow_Burden	Low-volume enrolling centre, defined as any participating institution falling within the lowest tertile of patient accrual in the PALMARES-2 study
**Final list of laboratory features**	
WBC_Baseline	Baseline white blood cell count (number of cells/mmc)
Neu_Baseline	Baseline neutrophil count (number of cells/mmc)
Lymph_Baseline	Baseline lymphocyte count (number of cells/mmc)
PLT_Baseline	Baseline platelet count (number of cells/mmc)
Mono_Baseline	Baseline monocyte count (number of cells/mmc)
Eosino_Baseline	Baseline eosinophil count (number of cells/mmc)
Baso_Baseline	Baseline basophil count (number of cells/mmc)
NLR	Neutrophil-to-lymphocyte ratio, calculated as following: neutrophil count / lymphocyte count
PLR	Platelet-to-lymphocyte ratio, calculated as following: platelet count / lymphocyte count

PIV	Pan-immune-inflammation value, calculated as following: neutrophil count x platelet count x monocyte count / lymphocyte count
MCV_Baseline	Mean corpuscular volume (fL)
AST_Baseline	Aspartate aminotransferase level (U/L)
ALT_Baseline	Alanine aminotransferase level (U/L)
Bili_Baseline	Total bilirubin level (mg/dl)
Crea_Baseline	Serum creatinine level (mg/dl)
Center_sizeMedium_Burden	Medium-volume enrolling centre
ET_adj_typeSequential	Sequence of tamoxifen and aromatase inhibitor, or vice versa, as the last adjuvant endocrine therapy for early-stage breast cancer
CDK46i_DrugAbemaciclib	Use of abemaciclib as first-line CDK4/6 inhibitor
HistologyOther	Histological subtype other than Classic Lobular Invasive or Non-Special Type
HRDUnknown	Unknown status of germline *BRCA1*, *BRCA2* or *PALB2* gene
BMI	Body Mass Index, computed as following: weight in kilograms divided by the square of height in meters (kg/m²)
Glycemia_Baseline	Baseline blood glucose (mg/dl)
CA153_Baseline	Baseline CA15.3 tumour marker (U/L)
ALP_Baseline	Alkaline phosphatase level (U/L)
LDH_Baseline	Lactate dehydrogenase level (U/L)

**Evaluated on the last tumour metastasis before first-line treatment initiation, if available; otherwise, value from primary tumour was considered.*
***As chronic therapy at the time of first-line treatment initiation, or within 30 days before first-line treatment initiation*

Supplementary Table S2. Characteristics of patients with tumours with ER-low (1-9%) expression vs those of patients with tumours with ER-intermediate expression (10-49%)

Characteristic	Overall<br>N = 157	ER <10%<br>N = 15	ER 10-49%<br>N = 142	p-value
CDK46i drug				0.2
Palbociclib	45 (29%)	7 (47%)	38 (27%)	
Ribociclib	49 (31%)	5 (33%)	44 (31%)	
Abemaciclib	63 (40%)	3 (20%)	60 (42%)	
Age (years; median, IQR)	58 (51, 68)	60 (56, 68)	56 (50, 68)	0.5*
ECOG PS				0.6
0	127 (81%)	11 (73%)	116 (82%)	
1	28 (18%)	4 (27%)	24 (17%)	
≥2	2 (1.3%)	0 (0%)	2 (1.4%)	
Histology				0.7
NST	113 (72%)	10 (67%)	103 (73%)	
ILC	38 (24%)	5 (33%)	33 (23%)	
Other	6 (3.8%)	0 (0%)	6 (4.2%)	
PgR expression	2 (0, 20)	5 (0, 30)	1 (0, 20)	0.3*

(%, median, IQR)				
Ki67 expression (%, median, IQR)	20 (15, 35)	20 (10, 30)	20 (15, 35)	0.3*
HER2 expression				0.8
0	81 (52%)	7 (47%)	74 (52%)	
Low	76 (48%)	8 (53%)	68 (48%)	
De novo aBC	25 (16%)	2 (13%)	23 (16%)	>0.9
Menopausal status				0.2
Postmenopausal	121 (77%)	14 (93%)	107 (75%)	
Premenopausal	36 (23%)	1 (6.7%)	35 (25%)	
Liver Metasteses	45 (29%)	2 (13%)	43 (30%)	0.2
Lung Metasteses	39 (25%)	5 (33%)	34 (24%)	0.5
Bone Metastases	102 (65%)	11 (73%)	91 (64%)	0.6
Bone-only disease				
Endocrine Resistance				0.2
Sensitive	81 (52%)	5 (33%)	76 (54%)	
Resistant	76 (48%)	10 (67%)	66 (46%)	
Luminal B-like^	98 (62%)	7 (47%)	91 (64%)	0.3

Data are presented as n (%) except where otherwise noted. The p value of the chi squared test is indicated in the right column of the table, except where otherwise noted. The p value of the test is indicated in bold numbers when statistically significant. p-values were calculated excluding unknown values. *Wilcoxon rank sum test. ^Defined as PgR expression <1% and/or Ki67 >20%. Abbreviations: CDK4/6i: cyclin-dependent kinase 4/6 inhibitors; ECOG PS: Eastern Cooperative Oncology Group Performance Status; ER: Oestrogen Receptor; HER2: Human Epidermal growth factor Receptor 2; ILC: Invasive Lobular Carcinoma; IQR InterQuartile Range; NST: Non-Special Type; PgR: Progesterone Receptor

Supplementary Table S3. Clinical characteristics of women included at the PALMARES-2 second data cutoff

**Characteristic**	**N = 4,187**
CDK46i drug	
Palbociclib	1,613 (39%)
Ribociclib	1,651 (39%)
Abemaciclib	923 (22%)
Age (years; median, IQR)	62 (53, 71)
ECOG PS	
0	3,270 (78%)
1	781 (19%)
≥2	136 (3.2%)
Histology	
NST	3,110 (74%)
ILC	777 (19%)
Other	300 (7.2%)
ERα expression (%, median, IQR)	90 (85, 95)
PgR expression (%, median, IQR)	40 (5, 80)
Ki67 expression (%, median, IQR)	20 (15, 30)
HER2 expression	
0	1,758 (42%)
Low	2,429 (58%)
De novo aBC	1,403 (34%)
Menopausal status	
Postmenopausal	3,444 (82%)

Premenopausal	743 (18%)
Liver Metasteses	887 (21%)
Lung Metasteses	1,057 (25%)
Bone Metastases	2,918 (70%)
Bone-only disease	1,041 (25%)
Endocrine Resistance	
Sensitive	2,826 (67%)
Resistant	1,361 (33%)
Luminal B-like^	2,351 (56%)

Data are presented as n (%) except where otherwise noted. ^Defined as PgR expression <1% and/or Ki67 >20%. Abbreviations: CDK4/6i: cyclin-dependent kinase 4/6 inhibitors; ECOG PS: Eastern Cooperative Oncology Group Performance Status; ERα: Oestrogen Receptor; HER2: Human Epidermal growth factor Receptor 2; ILC: Invasive Lobular Carcinoma; IQR InterQuartile Range; NST: Non-Special Type; PgR: Progesterone Receptor.

Supplementary Table S4. Unweighted and IPTW-weighted patient and tumor characteristics by CDK4/6 inhibitor group (palbociclib, ribociclib, abemaciclib), with corresponding SMDs before and after weighting.

Variable n (%)	Unweighted Palbociclib	Unweighted Abemaciclib	Unweighted Ribociclib	Weighted Palbociclib	Weighted Abemaciclib	Weighted Ribociclib	SMD Unweighted	SMD Weighted
Age(mean,SD)	64.09 (12.69)	62.15 (10.87)	58.98 (11.93)	61.19 (13.16)	61.66 (11.24)	61.44 (11.89)	0.43076	0.03916
ECOGPS0	1171 (72.6)	704 (76.3)	1395 (84.5)	1281.2 (78.9)	718.6 (78.1)	1300.1 (79.1)	0.28827	0.02464
ECOGPS1	374 (23.2)	188 (20.4)	219 (13.3)	293.1 (18.0)	171.5 (18.6)	295.2 (18.0)	0.25467	0.01727
ECOGPS≥2	68 (4.2)	31 (3.4)	37 (2.2)	50.2 (3.1)	30.5 (3.3)	48.9 (3.0)	0.11112	0.01935
Histology NST	1167 (72.3)	677 (73.3)	1266 (76.7)	1203.5 (74.1)	683.9 (74.3)	1222.7 (74.4)	0.09899	0.00656
Histology ILC	288 (17.9)	199 (21.6)	290 (17.6)	304.3 (18.7)	172.9 (18.8)	313.0 (19.0)	0.10196	0.00774
Histology Other	158 (9.8)	47 (5.1)	95 (5.8)	116.7 (7.2)	63.8 (6.9)	108.4 (6.6)	0.18644	0.02341
ERα (mean,SD)	87.54 (14.16)	86.02 (17.13)	87.04 (15.21)	86.92 (14.82)	87.18 (15.65)	86.78 (15.68)	0.09795	0.02561
PgR(mean,SD)	44.58 (35.75)	39.42 (37.90)	45.40 (36.04)	43.93 (35.77)	44.69 (38.38)	43.76 (36.27)	0.16352	0.02526
Ki67(mean,SD)	23.75 (15.12)	24.36 (14.49)	25.15 (15.49)	24.59 (15.74)	24.45 (14.49)	24.47 (15.30)	0.09283	0.00868
HER2low_	929 (57.6)	542 (58.7)	958 (58.0)	946.1 (58.2)	539.6 (58.6)	961.2 (58.5)	0.02284	0.00764
Denovo	489 (30.3)	240 (26.0)	674 (40.8)	537.0 (33.1)	306.3 (33.3)	541.3 (32.9)	0.31959	0.00768
Endocrine_Res	588 (36.5)	410 (44.4)	361 (21.9)	531.1 (32.7)	301.4 (32.7)	537.9 (32.7)	0.48479	0.00096
Endocrine_Sens	536 (33.2)	273 (29.6)	616 (37.3)	556.4 (34.3)	312.9 (34.0)	565.0 (34.4)	0.16437	0.00804
Menop_status: Premenopausal	213 (13.2)	122 (13.2)	408 (24.7)	299.1 (18.4)	157.7 (17.1)	296.7 (18.0)	0.30925	0.03457
Liver_Met	0.22 (0.42)	0.25 (0.43)	0.18 (0.38)	0.21 (0.41)	0.21 (0.41)	0.22 (0.41)	0.16988	0.01187
Lung_Met	0.25 (0.43)	0.27 (0.44)	0.25 (0.43)	0.25 (0.43)	0.25 (0.43)	0.25 (0.43)	0.05863	0.0113
Bone_Met	0.69 (0.46)	0.67 (0.47)	0.72 (0.45)	0.70 (0.46)	0.70 (0.46)	0.69 (0.46)	0.09154	0.01672

Data are presented as n (%) except where otherwise noted. Abbreviations: CDK4/6i: cyclin-dependent kinase 4/6 inhibitors; ECOG PS: Eastern Cooperative Oncology Group Performance Status; ERα: Oestrogen Receptor; HER2: Human Epidermal growth factor Receptor 2; ILC: Invasive Lobular Carcinoma; SD Standard Deviation; NST: Non-Special Type; PgR: Progesterone Receptor.SMD: Standardized Mean Differenca; IPTW: Inverse Probability of Treatment Weighting

Supplementary Table S5. Characteristic of patients with ERα expression status assessed at advanced disease diagnosis

Characteristic	Overall<br>N = 2947	ER <50%<br>N = 122	ER ≥90%<br>N = 2207	ER 50-89%<br>N = 618	p-value
CDK46i drug					0.053
Palbociclib	1050 (36%)	34 (28%)	798 (36%)	218 (35%)	
Ribociclib	660 (22%)	40 (33%)	476 (22%)	144 (23%)	
Abemaciclib	1,237 (42%)	48 (39%)	933 (42%)	256 (41%)	
Age (years; median, IQR)	62 (53, 71)	56 (51, 68)	63 (54, 72)	60 (50, 70)	<0.001*
ECOG PS					0.041
0	2302 (78%)	98 (80%)	1698 (77%)	506 (82%)	
1	537 (18%)	23 (19%)	423 (19%)	91 (15%)	
≥2	108 (3.7%)	1 (0.8%)	86 (3.9%)	21 (3.4%)	
Histology					0.079
NST	2204 (75%)	84 (69%)	1,649 (75%)	471 (76%)	
ILC	543 (18%)	32 (26%)	397 (18%)	114 (18%)	
Other	200 (6.8%)	6 (4.9%)	161 (7.3%)	33 (5.3%)	
PgR expression (%, median, IQR)	35 (1, 80)	0 (0, 15)	45 (5, 85)	15 (0, 60)	<0.001*
Unknown	16	3	10	3	
Ki67 expression (%, median, IQR)	20 (15, 30)	19 (13, 35)	20 (15, 30)	22 (15, 30)	0.5*
Unknown	246	32	154	60	
HER2 expression					0.020
0	1172 (41%)	53 (46%)	847 (40%)	272 (46%)	
low	1661 (59%)	61 (54%)	1,277 (60%)	323 (54%)	
Unknown	114	8	83	23	
De novo aBC	1388 (47%)	25 (20%)	1,066 (48%)	297 (48%)	<0.001
Menopausal_status					<0.001
Postmenopausal	2403 (82%)	95 (78%)	1851 (84%)	457 (74%)	
Premenopausal	544 (18%)	27 (22%)	356 (16%)	161 (26%)	
Liver Metasteses	658 (22%)	35 (29%)	494 (22%)	129 (21%)	0.2
Lung Metasteses	792 (27%)	29 (24%)	621 (28%)	142 (23%)	0.028
Bone Metastases	1928 (65%)	75 (61%)	1,446 (66%)	407 (66%)	0.6
Bone-only disease	567 (19%)	26 (21%)	409 (19%)	132 (21%)	0.2
Endocrine Resistance					<0.001
Sensitive	2300 (78%)	61 (50%)	1781 (81%)	458 (74%)	
Resistant	647 (22%)	61 (50%)	426 (19%)	160 (26%)	
Luminal B-like^	1702 (61%)	77 (71%)	1238 (59%)	387 (66%)	0.002
Unknown	157	13	115	29	

Data are presented as n (%) except where otherwise noted. The p value of the chi squared test is indicated in the right column of the table, except where otherwise noted. The p value of the test is indicated in bold numbers when statistically significant. p-values were calculated excluding unknown values. *Kruskal Wallis test. ^Defined as PgR expression <1% and/or Ki67 >20%. Abbreviations: CDK4/6i: cyclin-dependent kinase 4/6 inhibitors; ECOG PS: Eastern Cooperative Oncology Group Performance Status; ERα: Oestrogen Receptor; HER2: Human Epidermal growth factor Receptor 2; ILC: Invasive Lobular Carcinoma; IQR InterQuartile Range; NST: Non-Special Type; PgR: Progesterone Receptor

Supplementary Table S6. Sites of biopsies (excluding primary tumour) performed at advanced disease diagnosis

**Biopsy site**	N = 1787
Bone	441 (25%)
CNS	6 (0.3%)
Liver	291 (16%)
Lung	174 (9.7%)
Lymph nodes	360 (20%)
Other visceral site	210 (12%)
Skin/soft tissues	305 (17%)
Abbreviation: CNS: Central Nervous System	

Supplementary Table S7. Patient characteristics according to ERα and PgR expression category

Characteristic	Overall N = 4115	ERα≥90%,PgR ≥20% N = 1977	ERα≥90,PgR<20% N = 1014	ERα50-89%,PgR≥20% N = 577	ERα50-89,PgR<20% N = 390	ERα<50%,PgR≥20% N = 44	ERα<50%,PgR<20 % N = 113	p-value
CDK46i drug								<0.001
Palbociclib	1566 (38%)	762 (39%)	368 (36%)	238 (41%)	153 (39%)	14 (32%)	31 (27%)	
Ribociclib	918 (22%)	394 (20%)	271 (27%)	105 (18%)	99 (25%)	8 (18%)	41 (36%)	
Abemaciclib	1,631 (40%)	821 (42%)	375 (37%)	234 (41%)	138 (35%)	22 (50%)	41 (36%)	
Age (years; median, IQR)	62 (53, 71)	63 (53, 72)	63 (56, 71)	59 (49, 69)	60 (52, 70)	58 (52, 66)	58 (50, 70)	<0.001
ECOG PS								0.053
0	3214 (78%)	1522 (77%)	776 (77%)	477 (83%)	312 (80%)	35 (80%)	92 (81%)	
1	767 (19%)	394 (20%)	194 (19%)	82 (14%)	69 (18%)	9 (20%)	19 (17%)	
≥2	134 (3.3%)	61 (3.1%)	44 (4.3%)	18 (3.1%)	9 (2.3%)	0 (0%)	2 (1.8%)	
Histology								0.13
NST	3073 (75%)	1489 (75%)	747 (74%)	436 (76%)	288 (74%)	35 (80%)	78 (69%)	
ILC	770 (19%)	342 (17%)	201 (20%)	106 (18%)	83 (21%)	9 (20%)	29 (26%)	
Other	272 (6.6%)	146 (7.4%)	66 (6.5%)	35 (6.1%)	19 (4.9%)	0 (0%)	6 (5.3%)	
PgR expression (%, median, IQR)	40 (5, 80)	80 (50, 90)	1 (0, 5)	65 (40, 80)	0 (0, 5)	40 (30, 60)	0 (0, 5)	<0.001
Ki67 expression (%, median, IQR)	20 (15, 30)	20 (15, 30)	20 (15, 30)	20 (13, 30)	21 (15, 34)	15 (9, 33)	20 (15, 35)	0.007
HER2 expression								0.003
0	1837 (45%)	863 (44%)	419 (41%)	293 (51%)	181 (46%)	24 (55%)	57 (50%)	
Low	2278 (55%)	1114 (56%)	595 (59%)	284 (49%)	209 (54%)	20 (45%)	56 (50%)	
De novo aBC	1388 (34%)	762 (39%)	304 (30%)	201 (35%)	96 (25%)	10 (23%)	15 (13%)	<0.001
Menopausal status								<0.001
Postmenopausal	3381 (82%)	1627 (82%)	897 (88%)	419 (73%)	317 (81%)	33 (75%)	88 (78%)	
Premenopausal	734 (18%)	350 (18%)	117 (12%)	158 (27%)	73 (19%)	11 (25%)	25 (22%)	
Liver Metastases	878 (21%)	365 (18%)	279 (28%)	97 (17%)	92 (24%)	12 (27%)	33 (29%)	<0.001
Lung Metastases	1040 (25%)	541 (27%)	248 (24%)	131 (23%)	81 (21%)	12 (27%)	27 (24%)	0.045
Bone Metastases	2860 (70%)	1,386 (70%)	690 (68%)	444 (77%)	238 (61%)	31 (70%)	71 (63%)	<0.001
Bone-only disease	1013 (25%)	485 (25%)	219 (22%)	181 (31%)	91 (23%)	11 (25%)	26 (23%)	0.001
Endocrine Resistance								<0.001
Sensitive	2772 (67%)	1437 (73%)	629 (62%)	412 (71%)	213 (55%)	34 (77%)	47 (42%)	
Resistant	1343 (33%)	540 (27%)	385 (38%)	165 (29%)	177 (45%)	10 (23%)	66 (58%)	
Luminal B-like^	2343 (57%)	961 (49%)	749 (74%)	233 (40%)	302 (77%)	13 (30%)	85 (75%)	<0.001

Data are presented as n (%) except where otherwise noted. The p value of the chi squared test is indicated in the right column of the table, except where otherwise noted. The p value of the test is indicated in bold numbers when statistically significant. p-values were calculated excluding unknown values. *Kruskal Wallis test. ^Defined as PgR expression <1% and/or Ki67 >20%. Abbreviations: CDK4/6i: cyclin-dependent kinase 4/6 inhibitors; ECOG PS: Eastern Cooperative Oncology Group Performance Status; ER: Oestrogen Receptor; HER2: Human Epidermal growth factor Receptor 2; ILC: Invasive Lobular Carcinoma; IQR InterQuartile Range; NST: Non-Special Type; PgR: Progesterone Receptor

Supplementary Table S8. Patient characteristics according to CDK4/6i treatment in each ERα expression group

	ERα ≥90%				ERα 50-89%				ERα <50%			
	Palbociclib N = 1,130	Abemaciclib N = 665	Ribociclib N = 1,196	p-value	Palbociclib N = 391	Abemaciclib N = 204	Ribociclib N = 372	p-value	Palbociclib N = 45	Abemaciclib N = 49	Ribociclib N = 63	p-value
Age (years; median, IQR)	66 (56, 75)	64 (56, 71)	59 (50, 68)	<0.0012	61 (52, 72)	60 (52, 69)	57 (48, 67)	<0.0012	57 (52, 71)	60 (52, 67)	55 (49, 68)	0.82
ECOG PS				<0.0013				0.0713				0.114
0	785 (69%)	506 (76%)	1,007 (84%)		315 (81%)	157 (77%)	317 (85%)		34 (76%)	37 (76%)	56 (89%)	
1	294 (26%)	135 (20%)	159 (13%)		61 (16%)	41 (20%)	49 (13%)		11 (24%)	11 (22%)	6 (9.5%)	
≥2	51 (4.5%)	24 (3.6%)	30 (2.5%)		15 (3.8%)	6 (2.9%)	6 (1.6%)		0 (0%)	1 (2.0%)	1 (1.6%)	
Histology				0.0083				0.0093				0.94
NST	825 (73%)	491 (74%)	920 (77%)		289 (74%)	148 (73%)	287 (77%)		34 (76%)	34 (69%)	45 (71%)	
ILC	203 (18%)	136 (20%)	204 (17%)		70 (18%)	51 (25%)	68 (18%)		10 (22%)	12 (24%)	16 (25%)	
Other	102 (9.0%)	38 (5.7%)	72 (6.0%)		32 (8.2%)	5 (2.5%)	17 (4.6%)		1 (2.2%)	3 (6.1%)	2 (3.2%)	
PgR expression (%, median, IQR)	50 (5, 85)	40 (1, 90)	50 (10, 90)	0.0222	35 (2, 70)	20 (0, 60)	40 (5, 70)	0.0042	10 (0, 30)	0 (0, 5)	5 (0, 30)	0.0162
Ki67 expression (%, median, IQR)	20 (15, 30)	20 (15, 30)	21 (15, 30)	0.32	20 (12, 30)	20 (15, 33)	20 (15, 30)	0.0692	15 (10, 28)	20 (15, 40)	20 (15, 35)	0.0072
HER2 expression				0.63				0.53				0.63
0	495 (44%)	286 (43%)	501 (42%)		194 (50%)	93 (46%)	187 (50%)		26 (58%)	23 (47%)	32 (51%)	
Low	635 (56%)	379 (57%)	695 (58%)		197 (50%)	111 (54%)	185 (50%)		19 (42%)	26 (53%)	31 (49%)	
De novo aBC	374 (33%)	187 (28%)	505 (42%)	<0.0013	98 (25%)	50 (25%)	149 (40%)	<0.0013	7 (16%)	3 (6.1%)	15 (24%)	0.0403
Menopausal status				<0.0013				<0.0013				0.23
Postmenopausal	1,005 (89%)	593 (89%)	926 (77%)		316 (81%)	164 (80%)	256 (69%)		37 (82%)	40 (82%)	44 (70%)	
Premenopausal	125 (11%)	72 (11%)	270 (23%)		75 (19%)	40 (20%)	116 (31%)		8 (18%)	9 (18%)	19 (30%)	
Liver Metasteses	262 (23%)	175 (26%)	207 (17%)	<0.0013		77 (20%)	39 (19%)	73 (20%)	14 (31%)	15 (31%)	16 (25%)	0.83
Lung Metasteses	287 (25%)	186 (28%)	316 (26%)	0.53	91 (23%)	49 (24%)	72 (19%)	0.33	13 (29%)	13 (27%)	13 (21%)	0.63
Bone Metastases	761 (67%)	459 (69%)	856 (72%)	0.0843	277 (71%)	132 (65%)	273 (73%)	0.0903	35 (78%)	28 (57%)	39 (62%)	0.0903
Bone-only disease	279 (25%)	134 (20%)	291 (24%)	0.0633	119 (30%)	41 (20%)	112 (30%)	0.0163	12 (27%)	10 (20%)	15 (24%)	0.83
Endocrine Resistance				<0.0013				<0.0013				0.0523
Sensitive	733 (65%)	380 (57%)	953 (80%)		233 (60%)	111 (54%)	281 (76%)		23 (51%)	19 (39%)	39 (62%)	
Resistant	397 (35%)	285 (43%)	243 (20%)		158 (40%)	93 (46%)	91 (24%)		22 (49%)	30 (61%)	24 (38%)	
Luminal B-like^	622 (55%)	386 (58%)	702 (59%)	0.23	205 (52%)	128 (63%)	202 (54%)	0.0493	25 (56%)	36 (73%)	37 (59%)	0.153

*Data are presented as n (%) except where otherwise noted. The p value of the chi squared test is indicated in the right column of the table, except where otherwise noted. The p value of the test is indicated in bold numbers when statistically significant. p-values were calculated excluding unknown values. *Kruskal Wallis test. ^Defined as PgR expression <1% and/or Ki67 >20%. Abbreviations: CDK4/6i: cyclin-dependent kinase 4/6 inhibitors; ECOG PS: Eastern Cooperative Oncology Group Performance Status; ERα: Oestrogen Receptor; HER2: Human Epidermal growth factor Receptor 2; ILC: Invasive Lobular Carcinoma; IQR InterQuartile Range; NST: Non-Special Type; PgR: Progesterone Receptor*

Supplementary Table S9. Unweighted and IPTW-weighted patient and tumor characteristics by CDK4/6 inhibitor group, with corresponding SMDs before and after weighting in patients with ER-LI tumors

Variable n (%)	Unweighted_ Palbociclib	Unweighted_ Abemaciclib	Unweighted_ Ribociclib	Weighted_ Palbociclib	Weighted_ Abemaciclib	Weighted_ Ribociclib	SMD_ Unweighted	SMD_ Weighted
Age(mean,SD)	59.40 (11.89)	59.36 (10.68)	58.33 (13.46)	59.75 (12.66)	58.58 (9.59)	58.99 (12.54)	0.08895	0.09658
ECOGPS0	34 (75.6)	37 (75.5)	56 (88.9)	32.2 (77.2)	49.3 (83.8)	50.3 (79.8)	0.33859	0.16691
ECOGPS1	11 (24.4)	11 (22.4)	6 (9.5)	9.5 (22.8)	8.9 (15.1)	11.9 (18.9)	0.38743	0.20016
ECOGPS≥2	0 (0.0)	1 (2.0)	1 (1.6)	0.0 (0.0)	0.7 (1.1)	0.8 (1.3)	0.18731	0.12273
Histology NST	34 (75.6)	34 (69.4)	45 (71.4)	30.1 (72.2)	31.9 (54.2)	46.0 (73.0)	0.13778	0.42023
Histology ILC	10 (22.2)	12 (24.5)	16 (25.4)	10.5 (25.2)	25.4 (43.1)	15.5 (24.6)	0.07433	0.43293
Histology Other	1 (2.2)	3 (6.1)	2 (3.2)	1.1 (2.6)	1.6 (2.7)	1.5 (2.4)	0.20374	0.0168
PgR(mean,SD)	16.96 (23.42)	9.35 (20.68)	17.56 (23.51)	13.83 (19.91)	30.01 (36.05)	15.36 (22.13)	0.36363	0.71705
Ki67(mean,SD)	19.56 (13.42)	29.51 (20.19)	28.43 (19.55)	25.20 (16.92)	23.39 (18.07)	25.87 (17.30)	0.55365	0.13793
HER2low_	19 (42.2)	26 (53.1)	31 (49.2)	20.8 (49.8)	25.2 (42.8)	29.8 (47.3)	0.21781	0.1408
Denovo	7 (15.6)	3 (6.1)	15 (23.8)	6.5 (15.7)	19.1 (32.4)	11.4 (18.0)	0.50347	0.47406
Endocrine_Res	22 (48.9)	30 (61.2)	24 (38.1)	19.8 (47.6)	21.8 (37.1)	31.6 (50.2)	0.47111	0.26749
Endocrine_Sens	16 (35.6)	16 (32.7)	24 (38.1)	15.3 (36.7)	18.0 (30.6)	20.0 (31.8)	0.1139	0.12857
Menop_status: Premenopausal	8 (17.8)	9 (18.4)	19 (30.2)	8.2 (19.8)	22.9 (38.9)	14.2 (22.5)	0.30125	0.46602
Liver_Met	14 (31.1)	15 (30.6)	16 (25.4)	13.8 (33.1)	12.8 (21.8)	17.2 (27.3)	0.12608	0.2496
Lung_Met	13 (28.9)	13 (26.5)	13 (20.6)	8.8 (21.0)	10.9 (18.5)	13.1 (20.7)	0.19034	0.05863
Bone_Met	35 (77.8)	28 (57.1)	39 (61.9)	31.6 (75.9)	42.5 (72.1)	42.1 (66.7)	0.4421	0.19542

Data are presented as n (%) except where otherwise noted. Abbreviations: CDK4/6i: cyclin-dependent kinase 4/6 inhibitors; ECOG PS: Eastern Cooperative Oncology Group Performance Status; ERα: Oestrogen Receptor; HER2: Human Epidermal growth factor Receptor 2; ILC: Invasive Lobular Carcinoma; SD Standard Deviation; NST: Non-Special Type; PgR: Progesterone Receptor.SMD: Standardized Mean Differenca; IPTW: Inverse Probability of Treatment Weighting

Supplementary Table S10. Unweighted and IPTW-weighted patient and tumor characteristics by CDK4/6 inhibitor group, with corresponding SMDs before and after weighting in patients with ER-H tumors

Variable n (%)	Unweighted_ Palbociclib	Unweighted_ Abemaciclib	Unweighted_ Ribociclib	Weighted_ Palbociclib	Weighted_ Abemaciclib	Weighted_ Ribociclib	SMD_ Unweighted	SMD_ Weighted
Age(mean,SD)	61.59 (12.74)	60.13 (11.49)	57.52 (11.87)	59.45 (12.90)	59.99 (11.50)	59.53 (11.76)	0.33772	0.04451
ECOGPS0	315 (80.6)	157 (77.0)	317 (85.2)	323.8 (82.3)	164.5 (81.4)	306.7 (82.6)	0.21082	0.03062
ECOGPS1	61 (15.6)	41 (20.1)	49 (13.2)	59.2 (15.0)	32.6 (16.1)	54.5 (14.7)	0.18812	0.03981
ECOGPS≥2	15 (3.8)	6 (2.9)	6 (1.6)	10.4 (2.6)	5.1 (2.5)	10.3 (2.8)	0.13506	0.01621
Histology NST	289 (73.9)	148 (72.5)	287 (77.2)	292.4 (74.3)	152.9 (75.6)	276.5 (74.4)	0.10573	0.02943
Histology ILC	70 (17.9)	51 (25.0)	68 (18.3)	79.5 (20.2)	39.4 (19.5)	74.6 (20.1)	0.17672	0.01782
Histology Other	32 (8.2)	5 (2.5)	17 (4.6)	21.5 (5.5)	9.9 (4.9)	20.3 (5.5)	0.26291	0.0269
PgR(mean,SD)	38.04 (32.95)	31.37 (32.38)	39.03 (32.52)	36.82(32.91)	36.81 (33.30)	36.93 (32.77)	0.23483	0.0035
Ki67(mean,SD)	22.84 (15.43)	24.48 (14.34)	24.23 (14.51)	24.38(16.68)	23.40 (13.96)	24.07 (14.90)	0.11084	0.0662
HER2low_	197 (50.4)	111 (54.4)	185 (49.7)	200.6 (51.0)	102.9 (50.9)	191.2 (51.5)	0.09373	0.01109
Denovo	98 (25.1)	50 (24.5)	149 (40.1)	120.6 (30.7)	58.0 (28.7)	112.6 (30.3)	0.34388	0.04321
Endocrine_Res	158 (40.4)	93 (45.6)	91 (24.5)	138.9 (35.3)	70.4 (34.8)	130.5 (35.1)	0.44582	0.01042

Endocrine_Sens	135 (34.5)	61 (29.9)	132 (35.5)	133.8 (34.0)	73.7 (36.5)	128.4 (34.6)	0.11859	0.05199
Menop_status: Premenopausal	75 (19.2)	40 (19.6)	116 (31.2)	94.0 (23.9)	44.6 (22.1)	87.4 (23.5)	0.28627	0.04382
Liver_Met	77 (19.7)	39 (19.1)	73 (19.6)	77.4 (19.7)	42.2 (20.9)	74.7 (20.1)	0.01453	0.02984
Lung_Met	91 (23.3)	49 (24.0)	72 (19.4)	86.5 (22.0)	43.5 (21.5)	83.8 (22.6)	0.11235	0.02473
Bone_Met	277 (70.8)	132 (64.7)	273 (73.4)	274.1 (69.7)	138.7 (68.6)	258.2 (69.5)	0.1894	0.02366

Data are presented as n (%) except where otherwise noted. Abbreviations: CDK4/6i: cyclin-dependent kinase 4/6 inhibitors; ECOG PS: Eastern Cooperative Oncology Group Performance Status; ERα: Oestrogen Receptor; HER2: Human Epidermal growth factor Receptor 2; ILC: Invasive Lobular Carcinoma; SD Standard Deviation; NST: Non-Special Type; PgR: Progesterone Receptor.SMD: Standardized Mean Differenca; IPTW: Inverse Probability of Treatment Weighting

Supplementary Table 11. Unweighted and IPTW-weighted patient and tumor characteristics by CDK4/6 inhibitor group, with corresponding SMDs before and after weighting in patients with ER-VH tumors

Variable n (%)	Unweighted_ Palbociclib	Unweighted_ Abemaciclib	Unweighted Ribociclib	Weighted Palbociclib	Weighted_ Abemaciclib	Weighted_ Ribociclib	SMD_ Unweighted	SMD_ Weighted
Age(mean,SD)	65.08 (12.56)	62.96 (10.58)	59.40 (11.85)	61.75(13.24)	62.20 (11.21)	62.15(11.84)	0.48564	0.03777
ECOGPS0	785 (69.5)	506 (76.1)	1007 (84.2)	886.7 (77.7)	512.1 (76.9)	924.5 (77.6)	0.35138	0.01909
ECOGPS1	294 (26.0)	135 (20.3)	159 (13.3)	217.2 (19.0)	130.3 (19.6)	228.8 (19.2)	0.3216	0.0136
ECOGPS≥2	51 (4.5)	24 (3.6)	30 (2.5)	37.5 (3.3)	23.6 (3.5)	38.2 (3.2)	0.10855	0.01849
Histology NST	825 (73.0)	491 (73.8)	920 (76.9)	853.6 (74.8)	498.5 (74.8)	899.9 (75.5)	0.08998	0.01726
Histology ILC	203 (18.0)	136 (20.5)	204 (17.1)	206.5 (18.1)	121.2 (18.2)	216.2 (18.1)	0.08749	0.00296
Histology Other	102 (9.0)	38 (5.7)	72 (6.0)	81.4 (7.1)	46.3 (7.0)	75.3 (6.3)	0.13073	0.03205
PgR(mean,SD)	48.12 (37.00)	44.10 (39.18)	48.95 (37.00)	47.93(37.14)	48.56 (39.19)	47.87(37.13)	0.12841	0.01843
Ki67(mean,SD)	24.37 (15.23)	23.98 (14.03)	25.30 (15.63)	24.82(15.37)	24.84 (14.71)	24.72(15.54)	0.08809	0.00816
HER2low_	635 (56.2)	379 (57.0)	695 (58.1)	656.5 (57.5)	383.9 (57.6)	686.0 (57.6)	0.03871	0.00241
Denovo	374 (33.1)	187 (28.1)	505 (42.2)	397.8 (34.9)	239.0 (35.9)	414.9 (34.8)	0.299	0.02237
Endocrine_Res	396 (35.0)	284 (42.7)	243 (20.3)	355.9 (31.2)	204.8 (30.7)	374.3 (31.4)	0.48694	0.01461
Endocrine_Sens	360 (31.9)	194 (29.2)	448 (37.5)	387.7 (34.0)	222.3 (33.4)	402.3 (33.8)	0.17691	0.01249
Menop_status: Premenopausal	125 (11.1)	72 (10.8)	270 (22.6)	187.7 (16.4)	104.9 (15.7)	190.7 (16.0)	0.33465	0.02005
Liver_Met	262 (23.2)	175 (26.3)	207 (17.3)	248.3 (21.7)	142.0 (21.3)	269.6 (22.6)	0.21739	0.03158
Lung_Met	287 (25.4)	186 (28.0)	316 (26.4)	301.4 (26.4)	170.6 (25.6)	311.4 (26.1)	0.05822	0.01791
Bone_Met	761 (67.3)	459 (69.0)	856 (71.6)	797.8 (69.9)	461.4 (69.3)	821.9 (69.0)	0.09171	0.01971

Data are presented as n (%) except where otherwise noted. Abbreviations: CDK4/6i: cyclin-dependent kinase 4/6 inhibitors; ECOG PS: Eastern Cooperative Oncology Group Performance Status; ERα: Oestrogen Receptor; HER2: Human Epidermal growth factor Receptor 2; ILC: Invasive Lobular Carcinoma; SD Standard Deviation; NST: Non-Special Type; PgR: Progesterone Receptor.SMD: Standardized Mean Differenca; IPTW: Inverse Probability of Treatment Weighting
